# Inconsistent Definitions for Intention-To-Treat in Relation to Missing Outcome Data: Systematic Review of the Methods Literature

**DOI:** 10.1371/journal.pone.0049163

**Published:** 2012-11-15

**Authors:** Mohamad Alshurafa, Matthias Briel, Elie A. Akl, Ted Haines, Paul Moayyedi, Stephen J. Gentles, Lorena Rios, Chau Tran, Neera Bhatnagar, Francois Lamontagne, Stephen D. Walter, Gordon H. Guyatt

**Affiliations:** 1 Department of Clinical Epidemiology and Biostatistics, McMaster University, Hamilton, Canada; 2 Basel Institute for Clinical Epidemiology and Biostatistics, University Hospital Basel, Basel, Switzerland; 3 Departments of Medicine and Family Medicine, State University of New York at Buffalo, Buffalo, United States of America; 4 Department of Medicine, McMaster University, Hamilton, Canada; 5 Chau Tran Consulting, Toronto, Canada; 6 Centre de Recherche Clinique Étienne-Le Bel, Université de Sherbrooke, Sherbrooke, Canada; Johns Hopkins Bloomberg School of Public Health, United States of America

## Abstract

**Background:**

Authors of randomized trial reports seem to hold a variety of views regarding the relationship between missing outcome data (MOD) and intention to treat (ITT). The objectives of this study were to systematically investigate how authors of methodology articles define ITT in the presence of MOD, how they recommend handling MOD under ITT, and to make a proposal for potential improvement in the definition and use of ITT in relation to MOD.

**Methods and Findings:**

We systematically searched MEDLINE in February 2009 for methodological articles written in English that devoted at least one paragraph to ITT and two other paragraphs to either ITT or MOD. We excluded original trial reports, observational studies, and clinical systematic reviews. Working in teams of two, we independently extracted relevant information from each eligible article. Of 1007 titles and abstracts reviewed, 66 articles met eligibility criteria. Five (8%) did not provide a definition of ITT; 25 (38%) mentioned MOD but did not discuss its relationship to ITT; and 36 (55%) discussed the relationship of MOD with ITT. These 36 articles described one or more of three statements: complete follow-up is required for ITT (58%); ITT and MOD are separate issues (17%); and ITT requires a specific strategy for handling MOD (78%); 17 (47%) endorsed more than one relationship. The most frequently mentioned strategies for handling MOD within ITT were: using the last outcome carried forward (50%); sensitivity analysis (50%); and use of available data to impute missing data (46%).

**Conclusion:**

We found that there is no consensus on the definition of ITT in relation to MOD. For conceptual clarity, we suggest that both reports of randomized trials and systematic reviews separately consider and describe how they deal with participants with complete data and those with MOD.

## Introduction

Trial methodology experts, systematic review organizations, and authorities including the Consolidated Standards of Reporting Trials (CONSORT), [1] the Cochrane Collaboration, the US Food and Drug Administration, [2] the Nordic Council on Medicine in Europe, [3] and the American Statistical Associations Group [4] have recommended intention to treat (ITT) as the way to analyse randomized controlled trial (RCT) data. The goal of the ITT principle in RCTs is to preserve the prognostic balance between participants in treatment and control groups achieved through randomization and to thereby minimize selection bias and confounding. According to the principle, trial participants should be analyzed within the study group to which they were originally allocated irrespective of non-compliance or deviations from protocol. In superiority trials for which non-compliance would lower the apparent impact of effective interventions, the ITT strategy provides a conservative estimate of the treatment effect.

Trialists have widely adopted the term ITT when reporting RCTs. Surveys of RCT reports, however, suggest that simply stating ‘a study employed ITT’ is potentially misleading because of large variation in its definition and application. [5], [6] Missing outcome data (MOD) mainly due to patients lost to follow-up for the primary analysis was common (up to 75%) in RCTs reporting ITT, and trial investigators used a variety of methods to deal with MOD under ITT in the statistical analysis. [5], [6] Reviews about ITT in RCTs have concluded that ITT is often ‘misused’ or incorrectly applied.[5]–[8] Such a conclusion assumes that there exists a correct definition of ITT in relation to MOD. If there were a ‘correct’ definition of ITT, one would expect that definition to be uniformly or nearly uniformly applied in clinical trials and certainly to be standard among methodological articles. A consensus among clinical trialists and methodologists exists about how ITT applies to participants with available outcomes in superiority trials; they should be analysed in the groups to which they were randomized. However, trialists appear to hold different views regarding the relationship between MOD and ITT and how to address MOD under ITT. One explanation could be that trialists and authors of reporting guidelines usually turn to the ‘methodologic literature’ when seeking guidance about design and analysis issues in randomized trials. We therefore hypothesized that authors of methodology articles would hold a similarly heterogeneous view of ITT in relation to MOD, identifying a potential cause of the varying practice in clinical trials and a problem to be solved. Further, we hypothesized that possible solutions to the problem would emerge from the writings of authors taking various views about MOD in relation to ITT.

The primary objective of this study was to systematically investigate how authors of methodology articles define ITT when outcome data are not available in all participants and how they recommend handling MOD to conduct an analysis according to ITT. The secondary objective was to make a proposal for potential improvement in the definition and use of ITT in relation to MOD.

## Methods

The full details of the protocol are presented in the Methods section of M.A.’s MSc thesis (**Appendix S1**).

### Working Definitions

We understand loss to follow-up as the main reason for MOD, i.e. missing or incomplete ascertainment of the primary outcome for participants in an RCT. We did not take ambiguous terms like ‘drop-out,’ ‘withdrawal,’ and ‘not completing the trial’ (outcome ascertainment still possible) to necessarily mean MOD unless the authors of methodology articles made it clear they referred to loss to follow-up or MOD. Vice versa, we use the term “complete follow-up” to indicate no missing outcome data.

We considered as relevant methodology articles those that assessed how ITT was defined in other trial publications, discussed how ITT should be defined or reported, or examined methods that may be employed to address the issue of MOD when conducting an ITT analysis. These could include journal articles, reviews, editorials, or letters to the editor.

The concept of ITT has two main facets: (1) how to deal with participants for whom outcome data are available, and (2) how to deal with participants for whom outcome data are not available. If all participants had outcome data, all of the three ITT definitions (see below) on our data extraction form would lead to identical terminology and identical practice: analysis in the randomization arm for all participants for whom investigators have recorded the outcome of interest, regardless of protocol deviations and participant compliance. In case of MOD under ITT, we identified three mutually exclusive characterizations of ITT (i.e. the answer to the question “what is an ITT analysis”) and refer to these characterizations as definitions:

#### ‘Complete follow-up required’

This definition requires complete (100%) follow-up under ITT. That is, if any outcome data was missing then ITT is violated.

#### ‘Must or may use specific strategy for MOD’

An ITT analysis can be conducted in the presence of MOD as long as the MOD was handled in a particular, clearly specified manner. In this definition, the author could state that you ‘must’ impute data in a particular manner under ITT. Also under this definition, authors could state that you ‘may’ allow for more than one way to handle MOD by stating several ‘desirable’ strategies of dealing with MOD.

#### ‘ITT and MOD are separate issues’

In this definition, how one deals with MOD is irrelevant to the definition of ITT. In other words, ITT is conducted simply by ‘analyzing as randomized’ irrespective to how the investigator dealt with MOD.

### Eligibility Criteria

We included publications that devoted at least one paragraph to ITT and two other paragraphs to either ITT or MOD. Eligible articles mentioned the terms “intention to treat” or “intent to treat” in the title or abstract and were published in peer-reviewed journals either as articles, editorials, or letters to the editor. Because we were interested in capturing methodological discussions and fundamental concepts of ITT and not a description of how ITT was applied, we excluded original reports of RCTs, observational studies, and clinical systematic reviews. We excluded articles written in languages other than English.

### Search Strategy and Article Selection

A research librarian (N.B.) trained in health research methodology developed an initial pilot search strategy for MEDLINE. The librarian and an investigator (M.A.) subsequently used relevant articles identified through a pilot search to refine the search strategy. **Appendix S2** presents the detailed search strategy for MEDLINE using the Ovid platform to identify methodological papers from 1950 to February 2009.

Working in teams of two, investigators (E.A., M.A., C.T. and L.R.) independently screened titles and abstracts in duplicate. When one investigator deemed an abstract as potentially eligible, the full text document was retrieved. Next, pairs of investigators independently assessed eligibility of full text publications based on eligibility criteria. Disagreements were resolved by discussion and consensus or if necessary by third party arbitration (G.G.).

### Data Extraction

Investigators (M.A., T.H., S.G., L.R., and C.T.) working in teams of two independently extracted the different descriptions of ITT in relation to MOD from each eligible article using standardized, pre-piloted forms (**Appendix S3**). Data extractors chose from the following options to describe an author’s stance for each of the three definitions of ITT in relation to MOD above: ‘sole definition,’ ‘definition desirable,’ ‘definition possible but undesirable,’ ‘definition mentioned but preference unclear,’ ‘definition specifically excluded,’ and ‘definition not mentioned’ (see Table 4 in the thesis of M.A. (Appendix S1) for details about each of these categories). Our categorization system allowed us to capture instances in which an author held multiple definitions as ‘desirable’, but did not comment on a preference.

To accommodate new insights from articles we modified the data extraction form throughout the study to best reflect the data. [9] Three investigators adjudicated discrepancies between data extractors (G.G., T.H., and M.A.).

### Inter-rater Reliability

Agreement was calculated at the title and abstract screening and at the full text screening stage, as well as for categorical/dichotomous variables during data extraction. We used Kappa (κ) to determine the degree of agreement between pairs of reviewers and interpreted it according to Landis and Koch (κ values of 0 to 0.20 represent slight agreement; 0.21 to 0.40, fair agreement; 0.41 to 0.60, moderate agreement; 0.61 to 0.80, substantial agreement; and greater than 0.80 values represent almost perfect agreement). [10].

## Results

**Figure 1:**
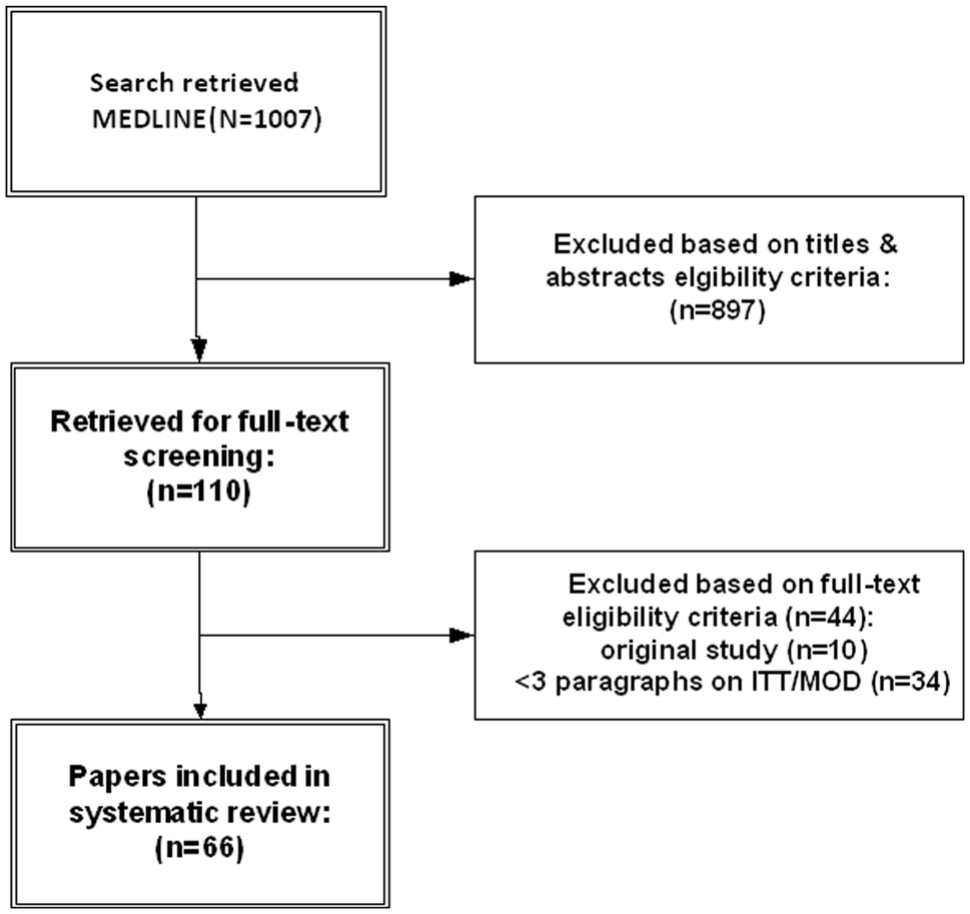
Flow diagram for articles included in this review. ITT, intention to treat; MOD, missing outcome data.

The MEDLINE search identified 1007 articles (**Figure 1**). Based on the relevance of the titles and abstracts, 110 of these articles underwent full text screening. Forty-four articles were excluded either because they had less than three paragraphs addressing ITT and MOD (n = 34) or because they were original studies (n = 10). In total, 66 articles met our eligibility criteria (see Appendix S4 for a complete list of included references).

There was substantial agreement among investigators for title and abstract screening (κ = 0.74; 95% confidence interval [CI], 0.67 to 0.80) and almost perfect agreement for the full text screening (κ = 0.81; 95% CI, 0.70 to 0.92). For duplicate extraction on the definition of ITT, extractors reached almost perfect agreement (κ = 0.87; 95% CI, 0.79 to 0.98) when averaging the kappas for all response categories.

The included articles discussed ITT and MOD on at least half a page and up to 15 pages and were published in journal types distributed among sub-specialty (46%), statistics (24%), methods (21%), and general medical journals (9%). Of the 66 articles, 5 (8%) did not provide a specific definition of ITT, 25 (38%) defined ITT and mentioned MOD but did not discuss its relationship with ITT, and 36 (55%) defined ITT and discussed the relationship of MOD to ITT.

Of the 36 articles that addressed MOD in relation to ITT, 19 (53%) mentioned a sole definition for ITT and 17 (47%) provided multiple possible definitions for ITT. Of the 19 that provided only one definition for ITT, 7 argued that complete follow-up is required under ITT, one that ITT and MOD are separate issues, and 11 that ITT involves specific strategy for MOD. Of the 17 that held more than one definition for ITT as possible, 15 (42%) articles provided two definitions, and two (6%) articles considered all three definitions as possible for ITT (**Table 1**).

**Table 1:** Definitions of intention to treat (ITT) in relation to missing outcome data (MOD).

	No (%) of articles (n = 66)
**Did not provide a definition of ITT**	5 (8)
**Did not discuss relationship between ITT and MOD**	25 (38)
**Addressed MOD in relation to ITT**	36 (55)
Provided one definition of ITT	19
*Complete follow-up is required under ITT*	7
*ITT and MOD are separate issues*	1
*ITT involves a specific strategy for MOD*	11
Provided multiple definitions of ITT *	17
*Complete follow-up is required under ITT*	14
*ITT and MOD are separate issues*	5
*ITT involves a specific strategy for MOD*	17

* For details please see **Appendix S4**, Table 1.

In total, 21 articles considered ‘complete follow-up required’ as a possible definition of ITT in relation to MOD: Seven (33%) concluded that this is the sole definition for ITT, 10 (48%) thought it was a desirable option, none thought it was undesirable, and 4 (19%) had an unclear preference. Seven articles specifically excluded this definition of ITT and 8 did not mention it. Soares and Carneiro, for instance, advocated the ‘complete follow-up required’ definition:

*“Other methods used to solve this problem include… carry forward of last observation response, explicit allocation of poor outcome, implicit assumption of good or poor outcome, and use of the group average… However, no imputation method can provide an unbiased assessment of the treatment effect unless the assumptions about the missing data are valid… Full application of intention-to-treat is possible only when complete outcome data are available for all randomized subjects”.* [11].

In the 28 articles that suggested ITT involves a specific strategy for MOD, complete case analysis (16 articles), last outcome carried forward (14 articles), sensitivity analysis (14 articles), and use of available data to impute missing data (13 articles) were the most frequently mentioned strategies. Less frequently mentioned were the worst case scenario (9 articles), the best case scenario (6 articles), ‘all had outcome event’ (8 articles), ‘all had no outcome event’ (5 articles), and multiple imputation (3 articles); 11 articles mentioned strategies other than these. Of the 16 articles that mentioned complete case analysis, 13 (81%) specifically excluded this strategy under ITT (**Table 2**).

**Table 2:** Strategies to deal with missing outcome data (MOD) under intention to treat (ITT).

ITT involves a specific strategy for MOD*	No (%) of articles (n = 28)
Complete case analysis	3 (11)
Worst case scenario	9 (32)
Best case scenario	6 (21)
Assumption all experienced outcome of interest	8 (29)
Assumption none experienced outcome of interest	5 (18)
Last observation carried forward	14 (50)
Censored at the time lost to follow-up in a survival analysis	12 (43)
Multiple imputation strategy	3 (11)
Sensitivity analysis (2 or more strategies should be used)	14 (50)
Other †	11 (39)

* Most articles suggested several strategies; for details see Appendix S4, Table 2.

† Details about „other strategies“ are summarized in Appendix S4, Table 3.

**Table 3:** Essential components to report in randomized clinical trials with respect to the analysis.

**Statement about intention to treat (ITT) for trial participants with available outcome data**
Claim of ITT: if individuals were analyzed in the groups to which they were randomized with details about any post-randomization exclusions *
No claim of ITT, e.g. if analysis exclusively focused on individuals who complied with the study protocol (‘per protocol’ or ‘as treated’ analysis)
**Statement about the handling of missing outcome data (MOD)** †
A) No MOD (complete follow-up)
B) Individuals with MOD were not considered in the analysis (complete/available case analysis)
C) Imputation with explicit description. Options include individuals with MOD were considered in the analysis:
i) assuming all experienced the outcome of interest, ‡
ii) assuming none experienced the outcome of interest, ‡
iii) assuming a worst case scenario (i.e. individuals with MOD in the experimental group experienced the outcome of interest and those in the control group did not), ‡
iv) assuming a best case scenario (i.e. individuals with MOD in the experimental group did not experienced the outcome of interest and those in the control group did), ‡
v) last observation carried forward,
vi) censored at the time lost to follow-up in a survival analysis,
vii) multiple imputation,
viii) any other imputation/modelling that needs to be specified.
D) Two or more of the options in B & C (sensitivity analysis)

* It may be appropriate to exclude randomized patients in order to achieve efficiencies while preserving prognostic balance between groups if two conditions are met [23]: (1) allocation to treatment or control could not possibly influence whether a particular randomized individual met criteria for post-randomization exclusion, (2) the decision about post-randomization is made without possible bias (commonly achieved through review blinded to allocation).

† There are various ways of handling missing data; we provide illustrative examples for reporting purposes.

‡ For dichotomous outcome data.

Unnebrink and Windeler, for example, list several methods of dealing with missing data that are valid strategies under ITT, but specifically excluded complete case analysis as a valid option: *“We examined a total of 14 ad hoc strategies for dealing with missing values. These can be roughly classified into numerical imputational strategies (last observation carried forward (LOCF), mean and regression based methods) and non-parametric strategies (rank and dichotomization based methods). We included CCA [complete case analysis] as the non-ITT strategy for reference purposes only.”* [12].

Six articles included, as a possible definition of ITT, our third characterization, i.e. ITT and MOD are separate issues. One argued that this was the sole definition for ITT. [13] Montori and Guyatt wrote: *“Intention-to-treat analysis cannot minimize bias introduced by loss to follow-up, that is, patients whose outcome status is unknown… To improve the applicability of study results to individual patients, investigators should improve study design to ensure protocol adherence with minimal loss to follow-up. Finally, loss to follow-up can result in exactly the same sort of bias as a per protocol analysis. Therefore, if there is significant loss to follow-up, statements that investigators conducted an “intention-to-treat analysis” generally provide little reassurance.”* [13] Eighteen articles specifically excluded this definition and 12 did not mention it.

## Discussion

### Summary of Findings

This systematic review of methodology articles found great variation in the proposed definitions of ITT in relation to MOD and in how MOD should be handled in RCTs under the ITT principle. Given the high proportion of RCTs with MOD for the primary outcome (60–75%) and the increasing use of the term ITT in RCT reports (up to 80%), this constitutes a serious problem. [5], [6], [14].

Although others have shown that the concept of ITT is inconsistently applied in clinical trials, [5], [6] the present review is the first to demonstrate that this variability in the definition of ITT extends to methodological articles. Not only did articles provide differing definitions, but 17 out of 36 articles (47%) that addressed the issue of MOD endorsed more than one definition of ITT.

### Limitations and Strengths

A potential limitation of our systematic review is that we focused our search exclusively on MEDLINE, articles in English, and articles that mentioned the terms “intention to treat” or “intent to treat” in the title or abstract potentially leading to less representative findings. Searching multiple databases and broadening our eligibility criteria might have increased the generalizability of our results. However, we would likely have found even more variability in definitions of ITT, suggesting that our findings in this respect are if anything conservative. Strengths of our review include our success in capturing a variety of journal types (methods, statistics, general medicine, and clinical specialty). We modified the data extraction form throughout the study to best reflect the data and to accommodate new insights from articles as they were acquired. [9] Furthermore, all articles were screened and abstracted in duplicate and experienced research methodologists adjudicated discrepancies.

We sought a criterion for a substantive discussion of the concepts of ITT and its relation to MOD and chose a criterion of three paragraphs. The criterion we chose is arbitrary, and one could argue that a criterion based on word rather than paragraph count would have been more appropriate. We found, however, that eligible articles did provide substantive discussion, and we were able to classify these articles according to their suggestions regarding ITT and MOD. Whether a less stringent criterion could have achieved the same goal with more eligible articles remains open to question.

### Suggested Strategies for Dealing with MOD in ITT

Of those who considered ITT and MOD to be related issues, the authors endorsed either imputing data and/or requiring complete follow-up. Of 16 articles that mentioned it, 13 (81%) excluded complete case analysis as a method of dealing with MOD under ITT; the others expressed the view that complete case analysis was a possibility under ITT, although it was an undesirable strategy. The position that MOD should be treated as a separate issue from ITT may implicitly accept complete case analysis as an acceptable alternative. Among clinical trialists, complete case analysis is the most popular approach to handle MOD: a recent review showed that complete case analysis was used in about half of RCTs reporting ITT. [5] These discrepancies highlight the current troubling state of the ITT principle in RCTs.

### Solution to the Problem

Several institutions have acknowledged that varying meanings of the term ITT is problematic. In its updated 2010 statement, CONSORT [15], [16] no longer advocates the use of the term ITT and *“replaced mention of ‘Intention to treat’ analysis, a widely misused term, by a more explicit request for information about retaining participants in their original assigned groups.”* [15] Still, the ITT definition by CONSORT strictly forbids the exclusion of any randomized individuals from the analysis, although a complete case analysis is deemed reasonable in the presence of MOD. [16] The Cochrane handbook has recently expanded its presentation of ITT. The current version of the handbook lists three principles of ITT: [17].

*“1. Keep participants in the intervention groups to which they were randomized, regardless of the intervention they actually received. 2. Measure outcome data on all participants. 3. Include all randomized participants in the analysis.”*

It acknowledges that *“there is no clear consensus on whether all criteria should be applied.* [*6*] *While the first is widely agreed, the second is often impossible and the third is contentious.”* If there are MOD the Cochrane handbook mentions two options, complete case analysis and data imputation, recommending a sensitivity analysis in either case. Both options involve assumptions about missing data and may be problematic, but using imputation achieves the ‘ITT quality label’ and the – in our view equally justified – complete case analysis does not. [17].

We propose a solution to the problem: First, we must separate the issue for which there is consensus, the analysis of those for whom outcome data is available, from the issue in which no consensus exists, how to conduct an analysis according to the ITT principle in the presence of MOD. Both issues are important items for assessing the risk of bias in RCTs. Substantial MOD as well as ‘per protocol’ or ‘as treated’ analyses may introduce bias. [18], [19] If investigators adopt the fundamental strategy of analysing those with complete outcome data in the groups to which they were randomized, they can claim ITT; one can then define four sub-categories of MOD under ITT (**Table 3**).

To date, while authors have suggested reasonable alternatives for handling MOD, [20] no compelling empirical evidence exists to guide the optimal data analysis which most likely depends on the context. Furthermore, investigators will inevitably have to make unverifiable assumptions about the missingness process, because they can never know what would have happened had missing data actually been observed. [21] Therefore, the goal should always be to transparently and clearly report how investigators dealt with MOD in their analysis. This is true both for individual trials and for systematic reviews/meta-analyses of RCTs. Subsequent empirical investigations could address the impact of various imputation approaches on the robustness of trial results. [22].

### Conclusion

We found a large variation in the definition of ITT in relation to MOD among methodology articles and how MOD should be handled in RCTs under ITT. We therefore propose to separate the ITT principle from handling of MOD in order to increase clarity and transparency of reporting trials and systematic reviews. We define four distinct sub-categories of ITT depending on the presence of patients with MOD and, if present, how investigators handle such patients in their analysis (**Table 3**). Use of this taxonomy would clarify the confusion in the current use of the term ‘ITT’.

## Supporting Information

Appendix S1 **MSc thesis of MA including study protocol.**
(PDF)

Appendix S2 **MEDLINE search strategy.**
(DOC)

Appendix S3 **Data extraction forms.**
(DOC)

Appendix S4 **Detailed results and list of included studies**
(DOC)

Checklist S1 **PRISMA Checklist.**
(DOC)
