# Supplementary material for: Inconsistent Definitions for Intention-To-Treat in Relation to Missing Outcome Data: Systematic Review of the Methods Literature
Source: PLoS One. 2012 Nov 15;7(11):e49163. doi: 10.1371/journal.pone.0049163 (PMC3499557; doi:10.1371/journal.pone.0049163)

THE INTENTION-TO-TREAT (ITT)

PROJECT

THE INTENTION-TO-TREAT (ITT) PROJECT: A SYSTEMATIC REVIEW OF THE METHODOLOGICAL LITERATURE

BY:

MOHAMAD ALSHURAFA, BHSC

A Thesis

Submitted to the Faculty of

Health Science at McMaster University

in Partial Fulfillment of the

Requirement for the Degree

MASTER OF SCIENCE

MCMASTER UNIVERSITY

© Mohamad Alshurafa, October, 2010

MASTER OF SCIENCE (2010) McMaster University

Clinical Epidemiology and Biostatistics Hamilton, Ontario

TITLE: The Intention-to-Treat (ITT) Project: A Systematic Review of the Methodological Literature

AUTHOR: Mohamad Alshurafa, BHSC

SUPERVISOR: Gordon Guyatt, M.D.

NUMBER OF PAGES: iv, 74

ABSTRACT

OBJECTIVES: The primary objective of this systematic review was to illustrate the various definitions authors of methodology articles have offered for intention to treat (ITT) in relation to loss to follow up (LTFU).

METHODS: We searched MEDLINE® for publications appearing between 1950 to 2008. Eligible papers devoted at least one paragraph to ITT and two other paragraphs to either ITT or LTFU. Investigators independently extracted relevant information from each eligible article. Discrepancies between data extractors were adjudicated. Data was extracted and variables used during data extraction were analyzed using the Landis and Koch guidelines kappa values.

RESULTS: The MEDLINE® search yielded 1007 articles. One-hundred and ten articles underwent full text screening yielding 66 articles. All kappa's were substantial to near perfect agreement (>0.74). Of the 66 articles, five (8%) did not define ITT, 25 (41%) mentioned LTFU but did not discuss its relationship with ITT, 36 (59%) commented on LTFU in the context of ITT. These 36 articles segregated into three distinctive definitions for ITT: "full-follow-up required" (mentioned 58%), "ITT and LTFU are separate issues" (mentioned 17%), and "ITT involves specific strategy for LTFU" (mentioned 78%). Of the 36 articles, 17 (47%) had multiple definitions for ITT. The most frequent strategies mentioned for handling LTFU were last outcome carried forward (50%), sensitivity analysis (50%), and use of available data to impute (46%). Most articles (81%) specifically excluded complete case analysis under ITT.

CONCLUSION: The most striking finding of our systematic review is that there is no meaningful consensus on the definition of ITT. This review, considered alongside previous reviews of RCTs, demonstrates that simply stating a study employed ITT is useless at best and misleading and dangerous at worst. It is the recommendation of this thesis that trialists replace the term ITT with a clear statements about analytic strategies applied to participants who were followed and with those not followed.

This thesis is dedicated to my mother, **Diana Borno**.

ACKNOWLEDGEMENTS

First and foremost, I thank God, the merciful and compassionate, for His blessings and for giving me the ability to explore the marvelous world of science. In the past few years I have worked with many individuals who have contributed in different ways to this research. This acknowledgment is one small token of my humble appreciation to them all.

I am indebted to my thesis supervisor, Gordon Guyatt, whose expertise in the mechanics of health research methodology has taught and inspired me. His encouragement and support from inception to completion has instilled in me a love for evidence-based medicine and has shaped my understanding of the subject. Gordon's extraordinary experiences and clearness of thought were invaluable and are found throughout this work. I owe my deepest gratitude to Ted Haines who supported me from the beginning of the master's. Much of my growth as a researcher is due to his teaching and his guidance throughout. I am indebted to him more than he knows. This thesis was borne out of the research led by Elie Akl's LOST IT study and for that I am indebted. Elie's work ethic and excellent leadership in the LOST IT study has served as an example of how to properly conduct a systematic review. I would like to acknowledge Stephan Walter for his advice and contributions that laid the foundation of this research. His statistical expertise has triggered many crucial methodological discussions that have fashioned the thesis. It is a pleasure to have worked with Stephen Gentles, Lorena Rios, Chau Tran and Neera Bhavnagar who have advanced the development of the search strategy, selection, data extraction and have contributed critically in committee meetings. I am indebted to the outstanding administrative support of Monica Owen whose willingness to provide her

professional help at any time has not gone unnoticed and is very much appreciated. I would like to express my gratitude to Dr. Paul Moyeddi for providing his unique perspective and his constructive remarks. Despite his many activities he agreed to chair the thesis defense and for that I am indebted.

I am truly thankful to my brothers and sisters Yusef, Dalia, Jihan, Hashem, Ehab, and Karim who have unselfishly passed on crucial life experiences along the way. Their support makes possible any success I ever accomplish. Karim deserves special mention as his friendship and care for others have influenced my life in incredible ways. To my dear friend, Muhammad Zubairi, I would like to thank you for being my inspiration for hard work and intellectual pursuit.

Simply put, this thesis would not have been possible unless my mother, Diana Borno, had encouraged higher education, provided emotional support, and instilled confidence in me. She is the person who inspired my learning character and raised me with her gentle and kind heart. I am forever thankful to my father, Nabil Alshurafa, whose wisdom, optimism and cheerfulness has shaped my personality in many ways. I am indebted to his endless support and caring.

Finally, words cannot convey my heartfelt appreciation for my wife, Rasha Kisswani, whose friendship and love have made me a better person. She selflessly gives her time, patience, and support for which I am eternally grateful and will require a lifetime to repay.

TABLE OF CONTENTS

CHAPTER 1: INTRODUCTION ___ 6

GENESIS OF THE IDEA FOR THIS PROJECT ___ 6

HISTORY OF THE DEFINITION ___ 9

THE PRIMARY PURPOSE OF ITT ___ 10

COMMON UNDERSTANDING: ITT IN CLINICAL TRIALS ___ 12

CHAPTER 2: METHODS ___ 16

OBJECTIVES ___ 16

ELIGIBILITY CRITERIA and INCLUSION CRITERIA ___ 17

EXCLUSION CRITERIA ___ 17

SEARCH STRATEGY ___ 17

STUDY SELECTION ___ 18

DATA EXTRACTION ___ 18

EXTRACTING THE DEFINITIONS OF ITT ___ 22

DEFINITIONAL ISSUES ___ 25

INTER-RATER RELIABILITY ___ 26

STATISTICAL ANALYSIS ___ 27

CHAPTER 3: RESULTS ___ 29

STUDY SELECTION ___ 29

HETEROGENEITY OF METHODOLOGY ARTICLES ___ 29

INTER-RATER RELIABILITY ______ *31*

DATA EXTRACTION ______ *32*

DOMAIN #1: DEFINITION OF ITT ______ *32*

COMMENTING ON LTFU IN THE CONTEXT OF ITT: SOLE VS. MULTIPLE DEFINITION ______ *32*

I. DEFINITION 'FULL-FOLLOW-UP REQUIRED' ______ *36*

II. DEFINITION 'ITT AND LTFU ARE SEPARATE ISSUES' ______ *36*

III. DEFINITION 'ITT INVOLVES SPECIFIC STRATEGY FOR LTFU': ______ *38*

ITT INVOLVES SPECIFIC STRATEGY FOR LTFU – TEN CATEGORIES ______ *38*

ITT INVOLVES SPECIFIC STRATEGY FOR LTFU – SUMMARY INFORMATION ______ *41*

STRATEGIES FOR DEALING WITH LTFU: LAST OUTCOME CARRIED FORWARD ______ *45*

STRATEGIES FOR DEALING WITH LTFU: SENSITIVITY ANALYSIS ______ *47*

STRATEGIES FOR DEALING WITH LTFU: "OTHER STRATEGIES" ______ *48*

AUTHORS HOLDING ITT AS THE GOLD STANDARD ______ *53*

STRNGTHS AND LIMITATIONS ______ *54*

CHAPTER 4: DISCUSSION ______ **56**

A WAY FORWARD ______ *63*

ENDNOTES ______ **67**

LIST OF TABLES AND FIGURES

Table 1: Definitions of various common strategies for handling LTFU 13

Table 2: Rates of reporting ITT in major medical journals.................................. 15

Table 3: Domains in the data extractions form 20

Table 4: Primary categories on the data extraction form.. 24

Figure 1: Flow diagram for articles included in this review................................. 30

Table 5: Distribution of included articles by journal type 31

Table 6 The number of articles that had sole definitions versus multiple definitions. 33

Table 7 The articles which held multiple definitions for ITT............................... 34

Figure 2 Definition of ITT 40

Table 8 The number of specific strategies suggested for handling LTFU. 41

Table 9 The authors who argued “ITT involves a specific strategy for dealing LTFU” definition. 43

Table 10: The ‘other strategies’ mentioned for handling LTFU under ITT. 50

Table 11: The basic definition provided by articles which commented on LTFU in the context of ITT.. 52

CHAPTER 1: INTRODUCTION

GENESIS OF THE IDEA FOR THIS PROJECT

This thesis was sparked by a discussion of a systematic review on the potential impact of loss to follow-up (LTFU).[1] This undertaking required us to define the commonly used term, “Intention-to-Treat” (ITT). Surprisingly, our research team, which included experienced methodologists, trialists, and statisticians, had differing opinions about the definition of ITT. During a discussion between three senior investigators, Stephan Walter quoted the Wiley Encyclopedia of Biostatistics definition to give an example of the argument that imputation of patients LTFU is necessary under ITT:

> *"Use of an ITT analysis requires... that all subjects with valid outcomes be i) included in the analysis, and ii) analysed according to their randomly assigned treatment."*
>
> - The Wiley Encyclopedia of Biostatistics [2]

The Wiley encyclopedia stated that all ‘valid outcomes’ be included in the analysis. However, it was unclear whether ‘valid outcome’ meant outcomes actually observed or outcomes imputed. Other investigators interpreted "valid outcome" as suggesting complete case analysis (excluding those LTFU, interpreted as those without a valid outcome, from analysis).

The Cochrane Handbook's definition of ITT also suggested ambiguity as identified by Dr. Walter:

> *“Cochrane specifies that we require "measured" outcomes on all participants for an ITT analysis. Does that imply directly observed values or admit other possibilities such as estimated, imputed or (even) assumed values? (I guess Cochrane would perhaps say "no" to the latter options?)”*
>
> *- S.W. Personal Communication (02/04/2008)*[3]

Douglas Altman subsequently confirmed that the Cochrane Handbook–at the time – restricted the use of ITT to situations where there is 100% follow-up.

We were surprised to have identified three conflicting definitions for ITT: one definition requiring full follow-up supported by Cochrane, and the other two definitions - either requiring ‘imputation’ of outcomes for those LTFU, or restricting the definition to refer to those with "valid" outcomes – depending on the interpretation, supported by Wiley. Gordon Guyatt expanded on the second interpretation of the Wiley definition: the term ITT should be applied only to those participants for whom outcome information is available:

> *“I think that the ITT principle should be restricted to those whose outcomes have been observed, who should be included in the analysis in the groups to which they are randomized. I don't think the term*

> *"ITT principle" should be used in reference to those not followed… Conceptual clarity is achieved by separating how one deals with those for whom one has outcome information from how one deals with those for whom one does not have follow-up information."*
>
> *– G.G., Personal Communication (02/05/2008)*[4]

The rationale for this definition was that ITT only refers to participants for whom follow-up data is available and how participants' LTFU are handled in the analysis has no bearing on the definition of ITT. This view was also endorsed by David Sackett, a senior investigator.[5] Since there was no agreement on what constitutes an ITT analysis, we decided to refrain from using the term ITT altogether in the protocol of our study examining loss to follow-up. However, since ITT is widely accepted and *required* by some regulatory organizations as a way to treat data from noncompliant participants, discarding the ITT term altogether was undesirable.

This alarming dilemma left us asking several questions. If a survey of our eleven member research team yielded three reasonable definitions for ITT, could there be a clear consensus of ITT in the literature? Is there a procedure for ITT that is consistently applied in the literature? Does the term ITT imply that we must achieve 100% follow-up or does it imply imputation of unknown outcome

data? Can ITT accommodate exclusion of patients such that the exclusion will not create prognostic imbalance?

HISTORY OF THE DEFINITION

'Principles of Medical Statistics (1961)' – Austin Bradford Hill

In their search for the definition of ITT, many were inclined to explore the origins of the term, giving full ownership to the individual who coined it. Although the concept was described in earlier literature by Bell (1941)[6], it appears that the late Austin Bradford Hill was the first to report the term 'intention to treat.' Our search found that Hill first used the term in 1961 in the 7th edition of his textbook entitled "Principles of Medical Statistics." Hill explains:

> *"Unless the losses are very few and therefore unimportant, we may inevitably have to keep such patients in the comparison and thus measure the **intention to treat** in a given way rather than the actual treatment. The question of the introduction of bias through exclusions for any reason (including lost sight of) must, therefore, always be carefully studied, not only at the end of a trial but throughout its progress. This continuous care is essential in order that we may immediately consider the nature of the exclusions and whether they must be retained for enquiry for follow-up, measurement etc. It will be too late to decide that at the end of the trial"* [7]

In this caption, Hill calls upon investigators to avoid bias through exclusion after randomization according to non-receipt of treatment. Hill mentions “losing sight” of patients, who might then be considered for exclusion. Hill explains that “losing sight” should be “carefully studied” as a cause of bias. Thus, Hill does not state that LTFU violates ITT nor does Hill specify a certain method of analysis for handling those with LTFU. This ambiguity may have been responsible, in part, for the differing interpretations of the definition of ITT.

THE PRIMARY PURPOSE OF ITT

The primary purpose of randomization in a controlled study is to create groups with similar characteristics and likelihoods that they will experience the outcome of interest. In other words, randomization establishes prognostic balance between groups. In clinical research, it is often the case that some participants or their caregivers do not adhere to the study protocol. Thus investigators are left with the decision of how to analyze the data obtained from those who dutifully participated but also the data from those who did not. Participants who do not adhere to the intervention often do not adhere for reasons related to the prognosis. In this circumstance, study authors often report using ITT as a method of reducing the potential systematic error which would result in excluding non-adherent individuals.

In a manner analogous to the problem of excluding non-adhering participants, loss to follow-up (LTFU), which we define as incomplete ascertainment of the outcome of interest, can destroy the prognostic balance created by randomization.

For conceptual clarity, it was useful to separate ITT into two parts: 1) what to do with non-compliant participants for whom one has ascertained an outcome and 2) what to do in cases of LTFU. There was no ambiguity, at least among members of our research group, for how to account for participants with an ascertained outcome. Our group agreed that the definition of ITT at least included a 'comparison of all participants for whom investigators have recorded the outcome of interest in the groups to which they have randomized, regardless of protocol deviations and participant compliance.' In other words, participants who did not follow instructions and were not LTFU were still included within the group to which they had been randomized.

The troubling ambiguity arose when we considered the second part of the ITT concept. It was not clear within our research group how to "properly" address the issue of LTFU.

COMMON UNDERSTANDING: ITT IN CLINICAL TRIALS:

The importance of ITT in controlled clinical trials is evidenced by its role in determining the methodological quality of trials and its ubiquitous presence in relevant guidelines and recommendations.[8,9,10] ITT has been recommended nearly universally as the analysis of choice for clinical trials. Governing bodies, such as the US Food and Drug Administration (FDA), specifically required ITT in clinical trials for medication approval. [11,12] Similarly, the Nordic Council on Medicine in Europe endorsed the use of ITT in clinical trials designed to establish the efficacy and safety of new medication.[13] The 2001 CONSORT (Consolidated Standards of Reporting Trials) statement, which encourages more consistent and thorough reporting, promoted a wider adoption of ITT in published reports.[14]

Despite its widespread use, several variations of how to deal with LTFU have been characterized as ITT.[15] Recently, several systematic reviews have addressed randomized controlled trials (RCTs) claiming an ITT analysis. These reviews examined the proportion of RCTs who reported an ITT as well as the strategy used for dealing with LTFU. A highly cited review by Hollis and Campbell (1999) surveyed all RCTs published in BMJ, Lancet, JAMA, and New England Journal of Medicine in 1997 (n=249) and showed 48% of trials reported ITT.[16] Out of those who claimed ITT, 75% of trials had LTFU and 24% had greater than 10% LTFU for their outcome of interest. Complete case analysis (see **Table 1** for definitions of

strategies for handling LTFU) was the most common method of analysis for dealing with missing data.

Strategy for Handling LTFU	Definition of Strategy
a. Complete case analysis (CCA)	Exclude participants LTFU from the analysis.
b. Worst case scenario (WCS)	Assume all participants LTFU in the treatment group had the event and none LTFU in the control had it.
c. Best case scenario (BCS)	Assume all participants LTFU in treatment group did NOT have the event and all LTFU in the control had it.
d. All had outcome	Assume all those LTFU had suffered the outcome of interest.
e. No patients outcome	Assume none of those LTFU had suffered the outcome of interest.
f. Use of available data to impute missing outcome	Available information on participants LTFU is used to assign outcome.
g. Last outcome carried forward (LOCF)	Last observed response in participants LTFU is used to assign the outcome.
h. Multiple imputation techniques	A technique which replaces each missing value with a set of plausible values which represent the uncertainty about the right value to impute.
i. Sensitivity analysis	Testing more than one assumption for those LTFU and looking at implications of alternative assumptions.

Table 1: Definitions of various common strategies for handling LTFU

Recently, Gravel et al. (2007) surveyed RCTs in 10 medical journals (n=249) and showed that 62% of trials reported using ITT.[17] Greater than 60% of the trials that report using ITT had LTFU as their primary outcome. Approximately 20% of trials had greater than 10% LTFU as their primary outcome. The methods for dealing with LTFU varied among trials; 60% used complete case analysis, 12% assumed no event for those LTFU, 8% used last outcome carried forward, 1% used multiple imputations, 2% ran other imputations, and 18% did not clearly identify which LTFU analysis was performed. Only 1% of RCTs conducted a worst case/best case scenario.

On the other hand, Wood et al. (2001) reviewed RCTs published in BMJ, Lancet, JAMA, and New England Journal of Medicine medical journals (n=71) in 2001 and found that 89% had LTFU for their primary outcome and 65% used complete case analysis.[18]

In the past two decades, reviews of clinical trials have seen an increase in the reporting of ITT in major medical journals from just under 50% in 1993-1997 [16-19] to 62% in 2002.[17] Some attributed the increase in reporting of ITT to the publication of the 2001 CONSORT statement. Gravel et al. (2007) review showed the proportion of reporting ITT was higher (84%) for journal adhering to the CONSORT statement (see **Table 2**).

Author, year of publication	Year trials extracted	Trials reporting ITT (%)
Ruiz-Canela et al., 2000	1993-1995	47.7
Hollis and Campbell, 1999	1997	48
Gravel et al., 2007	2002	62

Table 2: Rates of reporting ITT in major medical journals

These systematic reviews noted above, indicate that trialists who claim their analysis as ITT, handle LTFU differently. Given the high proportion of trials with LTFU and the increasing reporting of ITT, this presents a serious problem. As we found in our research team, trialists hold divergent views on the relationship that LTFU has with ITT.[14-17, 19] These data indicate a lack of consensus among trialists on how to best deal with LTFU. The purpose of this study was to investigate if an analogous disagreement exists among the authors of methodology articles since these individuals are presumably the experts in the mechanics of randomized clinical trial analysis.

CHAPTER 2: METHODS

OBJECTIVES

The primary objective of this systematic review was to illustrate the various definitions authors of methodology articles have offered for intention to treat (ITT) in relation to loss to follow up (LTFU).

There were several supplementary data which were also extracted in the course of performing this systematic review. The investigators systematically extracted data on the definition of modified-ITT (mITT), limitations of using ITT, biases which arise from running an analysis other than ITT, and possible differences or appropriateness of the use of ITT in preventative versus therapy treatment trials, management (practical, pragmatic, effectiveness) versus explanatory (mechanistic, efficacy) treatment trials, superiority versus non-inferiority or equivalence treatment trials. Furthermore, we compiled recommendations and comments relating to the following aspects of LTFU and the exclusion of patients: strategies for dealing with LTFU outside the context of ITT, circumstances in which exclusion of participants creates no bias, withdrawal of consent and the ethical imperative to exclude patients despite available data, and recommendation to achieve full follow-up. This thesis will not deal with the results of the extracted supplementary data.

INCLUSION CRITERIA

We included articles which devoted at least one paragraph to ITT and two other paragraphs to either ITT or LTFU. Eligible articles could be published in peer-reviewed journals, editorials and letters to the editor.

EXCLUSION CRITERIA

We excluded original reports of randomized trials, observational studies, or clinical systematic reviews. This is because we were interested in capturing methodological discussions and fundamental concepts of ITT and not a description of how ITT was applied in an RCT. Moreover, original reports often simply used the term ITT without defining the concept or explaining how the term was applied. Non-English studies were also excluded.

SEARCH STRATEGY

A research librarian (N.B.) trained in health research methodology developed an initial pilot search strategy. The librarian and an investigator (M.A.) subsequently used relevant articles identified by the pilot search strategy and independently by the investigators (E.A., G.G., S.G., and M.A.) to refine the search strategy. For peer-reviewed journals we used MEDLINE®® to search for methodological articles from 1950 to December 2008. (See **Appendix A Figure 1** for pilot search strategy and **Figure 2** for search strategy.)

STUDY SELECTION

Four investigators (E.A., M.A., C.T. and L.R.) independently screened articles in duplicate based on the titles and abstracts using the eligibility criteria in **Appendix A Figure 3**. Once the full-text of the article was retrieved, investigators (M.A., C.T., and L.R.) independently screened articles in duplicate based on the eligibility criteria described in **Appendix A Figure 4**.

DATA EXTRACTION

Investigators (C.T., L.R., M.A., S.G., G.G., and T.H.) independently extracted relevant information from each eligible article using standardized, pre-piloted forms. Three investigators adjudicated discrepancies between data extractors (G.G., T.H., and M.A.).

The method for developing the data extraction form was to first identify areas of interest we called 'domains' that were based on our *a priori* research questions (**see Appendix A Table 1** for the *a priori* research questions). For example, domain #1 captured instances in which an author mentioned the definition of ITT (our primary research question). For purposes of categorization, this domain included a list of possible definitions for ITT. Similarly, domain #2 captured information on mITT and domain #4 captured instances in which authors' mentioned the limitations of ITT.

In the first version of the data extraction form, seven domains were developed with senior investigators (S.W. and G.G) in committee meetings. These original domains served as the basis for pilot data extraction. The first version of the extraction form is included in **Appendix B Figure 1 (*Data Extraction Form, Version 1)***. In its final iteration, the data extraction form contained 13 domains as shown in **Table 3**. Although investigators extracted data from studies using all 13 domains, this thesis focused on the results from domains #1 and #13 (see **Appendix B Figure 2 Final Data Extraction Form)** for data extraction forms containing all the domains).

Pilot data extractions were conducted by two investigators (L.R. and M.A.) with the objective of further developing the data extraction form.

Information from the pilot data extractions was used to improve and expand the data extraction form. When a common answer was identified it was added as a check-box or as an item in a pull-down menu in the extraction form. For example, as additional definitions for ITT and mITT were identified through extractions, domains #1 & #2 were modified to capture these definitions.

**Domain Number**	**Question**
Domain #1	What are the different ways of defining ITT in relation to LTFU?
Domain #2	What is the definition of modified ITT?
Domain #3	What are recommendations for dealing with LTFU outside of the context of ITT?
Domain #4	What are the limitations of ITT?
Domain #5	When exclusion of participants does not convey bias?
Domain #6	Biases which arise if an analysis other than an ITT is performed?
Domain #7	Is there an ethical issue with including available participant outcome data for those patients who withdrew their consent to participate in the trial?
Domain #8	What are some strategies or recommendation to achieve full follow-up in a trial?
Domain #9	What are the differences or the implications of using ITT in preventative versus therapy treatment trials?
Domain #10	What are the differences or the implications of using ITT in management (practical, pragmatic, effectiveness) versus explanatory (mechanistic, efficacy) treatment trials?
Domain #11	What are the differences or the implications using ITT in superiority versus non-inferiority or equivalence treatment trials?
Domain #12	What are the differences or the implications of using ITT for data missing completely at random versus missing at random versus missing nonrandomly?
Domain #13	How much of the article was devoted to the definition of ITT?

Table 3: Domains in the data extractions form

In order to capture the richness and complexity of the data, every domain on the form included a section in which to write interesting, unique, or

important qualitative findings pertaining to that domain (e.g. Domain 1, 'Main Points' Section). In addition, text-boxes containing open-ended questions were added in several domains to provide another area in which qualitative data could be added (e.g. Domain 1, question 1j). By the end of the pilot extraction process, 11 domains had been developed, and the basic backbone of the data extraction form had emerged (See **Appendix B Figure 2** Version 9 Data Extraction Form).

The data extraction form continued to evolve throughout the study and new relevant domains were added. At one point in the analysis, for example, we considered an article in which Gross (2004)[20] discussed how ITT should be applied in preventative clinical trials. Thus domain #9 was created to extract this specific information. All investigators were encouraged to suggest modifications to the data extraction form and it was continually improved until no new modifications to the form were suggested.

When an important item was added to the data extraction form, one investigator (M.A.) reviewed all the articles an additional time using the more updated form. Thus, all the data from this review was ultimately derived from the final version of the data extraction form. See **Appendix B Figure 3** for the full and final version of the data extraction form.

EXTRACTING THE DEFINITIONS OF ITT

The first domain of the data extraction form captures how authors define ITT in relation to LTFU, which was our main research question. The concept of ITT has two main facets: how to deal with participants for whom one has an outcome and how to deal with participants who are LTFU. If a participant had an outcome, each of three ITT definitions (identified below) on the data extraction form was identical: comparison of all participants as randomized for which investigators had recorded the outcome of interest, regardless of protocol deviations and participant compliance. In cases of LTFU under ITT, each of the three definitions was unique and is identified below:

Definition 'full-follow-up required':

This definition requires complete (100%) follow-up under ITT. That is, if even one patient was LTFU then ITT is violated.

Definition 'must or may use specific strategy for LTFU':

An ITT analysis can be conducted in the presence of LTFU as long as the LTFU was handled in a particular, clearly specified manner. In this definition, the author could state that you 'must' impute data in a particular manner under ITT. Also under this definition, authors could state that you 'may' allow for more than

one way to handle LTFU by stating several 'desirable' strategies of dealing with LTFU. In the form, we provided a list of several commonly encountered options for handling LTFU for extractors to choose from. This list included various imputation methods or the option of running a complete-case analysis for either binary or continuous variables (See **Table 4**).

Definition "ITT and LTFU are separate issues":

ITT and LTFU are separate issues. In this definition, how one deals with LTFU is irrelevant to the definition of ITT. In other words, ITT is conducted simply by 'analyzing as randomized' irrespective to how the investigator dealt with LTFU.

In the final data extraction form, extractors chose from a drop down menu with the following options to describe an author's stance for each of the three definitions above: 'sole definition,' 'definition desirable,' 'definition possible but undesirable,' 'definition mentioned but preference unclear,' 'definition specifically excluded,' and 'definition not mentioned.' See **Table 4** for the explanation of each of these categories.

**Categories Describing an Authors Stance on ITT Definitions**	**Explanation of Categories**
1) “Definition Possible"	The author thought a particular definition of ITT was possible, subcategories i) to iv) below
i. "Sole Definition”	The author thought only one definition of ITT was possible
ii. “Definition Desirable"	The author thought more than one definition of ITT was possible and endorsed a particular definition of ITT
iii. “Definition Undesirable"	The author thought more than one definition of ITT was possible and thought a particular definition of ITT was undesirable
iv. “Definition Preference Unclear"	The author thought more than one definition of ITT was possible and showed no preference for a particular definition of ITT
2) “Definition Specifically Excluded"	The author specifically excludes a particular definition as ITT or an author holds an opposing definition as the ‘sole’ definition for ITT
3) “Definition Not Mentioned”	The author did not mention a particular definition

Table 4: Primary Categories on the Data Extraction Form. The following categories were available as drop-down menus in the data extraction form and were used to describe an author’s stance on a particular definition of ITT.

Some assumptions were made when presenting the wording shown in Table 4. Particularly, the articles which explicitly mentioned a ‘sole definition’ for ITT were marked as ‘specifically excluding’ the other definitions. For example, if

an article stated that full follow-up was the 'sole' definition then automatically it was flagged as 'specifically excluding' the definitions 'ITT is a separate issue' and 'ITT involves specific strategy for LTFU.' Similarly, when articles stated that ITT is a separate issue from LTFU was the 'sole definition,' then they were flagged as specifically excluding the other two definitions. We also assumed that if articles explicitly stated a specific strategy for dealing with LTFU as a 'sole definition' of ITT then they were flagged as specifically excluding the other two definitions.

DEFINITIONAL ISSUES

During data extraction, definitional issues arose and were resolved on a case-by-case basis at periodic meetings. One important definitional issue which arose was how to summarize an author's stance on any of the definitions of ITT when the author endorsed more than one definition. The first iteration of the form used the word 'preferred definition' as in, the author 'preferred' Definition 'full-follow-up required'. However, we found that the word 'preferred' implied the author held one definition as superior over another definition, which was not always the case. Moreover, the word 'preferred' did not account for the articles which included more than one definition of ITT and made no preference for one definition over the other. To resolve this issue, we substituted the word 'preferred' with 'desirable'. This allowed us to capture instances in which an

author held multiple definitions as ‘desirable’ and did not comment on a preference.

Another example of a definitional issue was the ambiguity in terms like ‘drop-out,’ ‘withdraw,’ and ‘not completing the trial.’ As previously mentioned, we defined LTFU as incomplete ascertainment of the outcome of interest for participants randomized in a trial. However, terms like ‘withdrawal’ did not necessarily mean the primary outcome was incompletely ascertained (examples of this are discussed in the results). Thus, in this review, we did not take ambiguous terms like ‘drop-out,’ ‘withdraw,’ and ‘not completing the trial’ to necessarily refer to LTFU unless the authors stated that it did. Each data extractor made a judgment on whether the author was referring to LTFU. This judgment was based on the definition the author of the methodology article provided (if one was provided) or statements the author made whether primary outcome data was complete. Unresolved discrepancies between extractors resulting from a definitional issue were adjudicated.

INTER-RATER RELIABILITY

Agreement was calculated at the title and abstract screening, the full text screening, and the categorical/dichotomous variables during data extraction. The proportion of agreement and kappa statistic were used to determine the

degree of agreement between pairs of reviewers. All calculations of inter-rater reliability were performed with Microsoft Excel for Windows. Interpretation of the agreement statistics was done using the Landis and Koch[21] guidelines kappa values of 0 to 0.20 represented slight agreement; 0.21 to 0.40, fair agreement; 0.41 to 0.60, moderate agreement; 0.61 to 0.80, substantial agreement; and greater than 0.80 represented almost perfect agreement.

STATISTICAL ANALYSIS

All analyses were conducted by one investigator (M.A) using Microsoft Excel and were repeated for validation.

Considering that it would not be feasible to capture all methodological articles, we excluded those articles which only briefly mention ITT. To be eligible, articles were required to mention the relevant topic in a minimum of three paragraphs. Since we were particularly interested in capturing methodological articles which discussed the ITT principle in relation to LTFU, we included articles which discussed ITT in at least one paragraph, as long as the articles contained at least two additional paragraphs which mentioned either ITT or LTFU. We did this in order to increase representation of articles which treat LTFU as a separate issue from ITT. Eligible articles were published in peer-reviewed journals and we included editorials and letters to the editor.

There is no agreed search strategy on how best to capture ITT methodology papers. Despite using an experienced librarian, eligible papers (even within Medline) may have been missed and this may have impacted the findings. However, because we were particularly interested in the variety of definitions of ITT, using only one database would make our analysis more conservative. As it turned out, MEDLINE® alone proved sufficient because of the wide variety of ITT definitions we found.

CHAPTER 3: RESULTS

STUDY SELECTION

The MEDLINE® search yielded 1007 articles. Based on the relevance of the title and abstracts, 110 of these articles underwent full text screening. Forty-four articles were deemed ineligible either because they had less than three paragraphs addressing ITT and LTFU (n=33) or because they were original studies (n=10). In total, sixty-six articles were included after the full text screening. The study flow diagram is shown in **Figure 1**.

HETEROGENEITY OF METHODOLOGY ARTICLES

On average, included articles were eight pages (interquartile range [IQ], 5-11) in length excluding references and had approximately four pages [IQ, 1-6] of discussion on ITT. The articles ranged from a half a page to 15 pages solely discussing ITT and LTFU. Our sample included a variety of different journal types distributed among specialty, statistics, methods and general medicine journals as shown in **Table 5**. For a list of included studies, year published, and journal name, see **Appendix C Table 1**.

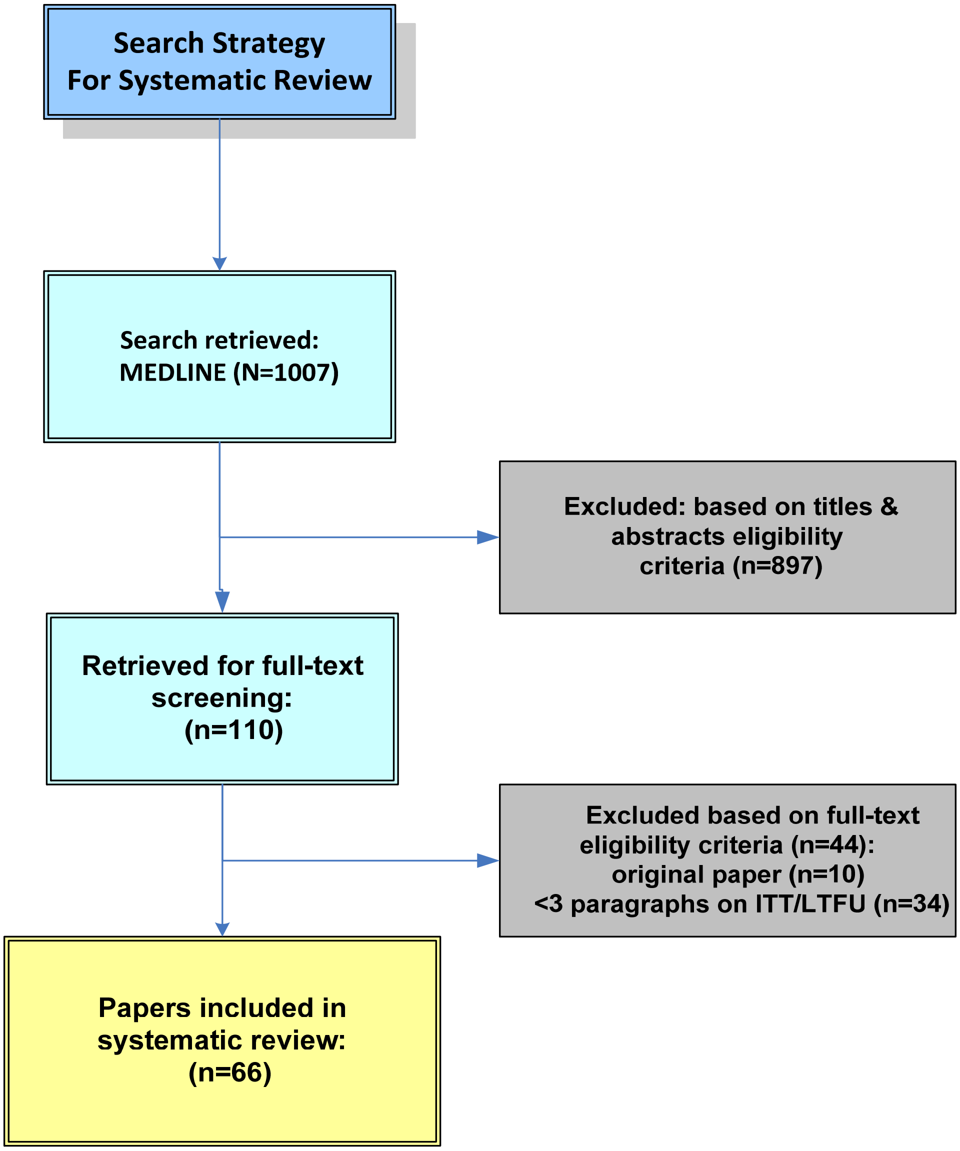

Figure 1: Flow diagram for articles included in this review

Type of Journal	Frequency
Sub-Specialty	30 (45%)
Statistics	16 (24%)
Methods	14 (21%)
General Medicine	6 (9%)
**Total**	**66**

Table 5: Distribution of included articles by journal type

INTER-RATER RELIABILITY

There was substantial agreement for title and abstract screening and almost perfect agreement for the full text screening as the kappa values were 0.74 (95%CI; 0.670, 0.80) and 0.81 (95%CI; 0.70, 0.92), respectively. Duplicate extraction for the first domain (on the definition of ITT) of the data extraction form established almost perfect agreement 0.87 (95%CI; 0.79, 0.98) when averaging the kappas for all drop-boxes and subcategories in the domain. When only averaging the drop boxes for the three main definitions in domain #1, the kappa was also almost perfect at 0.84 (95%CI; 0.74, 0.98).

DATA EXTRACTION

DOMAIN #1: DEFINITION OF ITT

Five of the 66 articles which met eligibility criteria (8%) did not define ITT and therefore were not considered further.[23-26] Of the 61 articles which defined ITT, 25 (41%) mentioned LTFU but did not discuss its relationship with ITT (see **Appendix E**).[27-51] The remaining 36 (59%) commented on LTFU in the context of ITT.[52-87] These 36 articles fall under three definitions: 'full follow-up required,' 'ITT and LTFU are separate issues,' and 'ITT involves specific strategy for LTFU.'

ARTICLES COMMENTING ON LTFU IN THE CONTEXT OF ITT: SOLE VS. MULTIPLE DEFINITION

Of the 36 articles which mentioned LTFU in relation to ITT, 19 (53%) mentioned a sole definition for ITT and 17 (47%) had multiple definitions for ITT. Of the 19 which mentioned a sole definition for ITT, seven argued 'full follow-up required' under ITT, one argued 'ITT and LTFU are separate issues,' and 11 argued 'ITT involves specific strategy for LTFU' (see **Table 6**). Of the 17 which had more than one definition for ITT, 15 (42%) articles mentioned two definitions, and two (6%) articles mentioned all three definitions for ITT as possible. In total, the 'full follow-up required' definition was given by 21 articles, 'ITT and LTFU are separate issues' was given by six articles, and the 'ITT involves specific strategy

for LTFU' was given by 28 articles. **Table 7** presents the 17 articles which provide multiple definitions for ITT.

Definitions	No. Articles (n=36)
Sole Definition: 'Full follow up required under ITT'	19% (7)
Sole Definition: 'ITT and LTFU are separate issues'	3% (1)
Sole Definition: 'ITT involves specific strategy for LTFU'	31% (11)
Multiple definitions mentioned	47% (17)

Table 6 The number of articles that had sole definitions versus multiple definitions. Of the 36 articles which mentioned LTFU in the context of ITT, 19 had a sole definition and 17 had more one definition.

Author, year	Definition 'Full follow up required under ITT'	Definition 'ITT and LTFU are separate issues'	Definition 'ITT involves specific strategy for LTFU'
[52]Altman, 2001	X	X	X
[53]Bailey, 1994	X		X
[55]Bubbar, 2006		X	X
[56]D'Agostino, 2004		X	X
[60]Gravel, 2007	X		X
[61]Green, 2000	X		X
[62]Hollis, 2002	X		X
[63]Hollis, 1999	X		X
[67]Kruse, 2002	X		X
[68]Lewis, 1993	X	X	X
[71]Nich, 2002	X		X
[74]Sato, 2001	X		X
[76]Soares, 2002	X		X
[84]Wiens, 2007	X		X
[82]White, 2005		X	X
[83]Whittaker, 2006	X		X
[85]Wright, 2003	X		X

Table 7 The articles (n=17) which held more than one possible definition of ITT listed by author

As shown in **Table 7**, Wright et al.[52] is an example of an author providing more than one possible definition for ITT:

> *"Although imputation may be regarded as the appropriate method of dealing with missing values in an ITT analysis, the optimum way of dealing with missing data is to ensure, through appropriate design and conduct, that as few data as possible are missing in the first place"* – pg 840

Here, the author mentioned two definitions for ITT; endorsing full follow-up as the 'optimum' definition, and also acknowledging imputation as an ‘appropriate’ way of dealing with missing data in an ITT analysis.

As mentioned previously, the 36 articles which commented on LTFU in relation to ITT fall into three categories: Definition ‘full-follow-up required,’ Definition ‘ITT and LTFU are separate issues,’ and Definition ‘ITT involves specific strategy for LTFU.’ Categories are not mutually exclusive, as one author can mention more than one definition of ITT, thus the totals may be greater than 100%.

I. DEFINITION 'FULL-FOLLOW-UP REQUIRED':

ITT is restricted to situations in which 100% of the participants are followed up for the outcome of interest. Of the 36 articles which comment on LTFU in the context of ITT, authors of 21 articles (58%) stated that the 'full follow-up required' definition was a possible definition for ITT. Of these 21 articles, seven (33%) articles concluded that this definition is the sole definition for ITT, 10 (48%) thought it was a desirable option, none thought it was undesirable and four (19%) had an unclear preference. Seven specifically excluded this definition of ITT and eight did not mention the definition.

II. DEFINITION 'ITT AND LTFU ARE SEPARATE ISSUES':

Participants who have the outcome of interest are analyzed as randomized and the strategy for dealing with patients LTFU has no bearing on the definition of ITT. Of the 36 articles, six (17%) mentioned this position as possible. Out of the six articles, one (17%) article by Montori and Guyatt[69] argued that this was the sole definition for ITT. No articles took the position that this definition was either desirable or undesirable. Of the six articles that mentioned this position as possible, five (83%) did not list a clear preference regarding this definition. Finally, of the 36 articles which commented on this definition, 18 (50%) specifically excluded this definition and 12 did not mention the definition.

In the following excerpts, Montori and Guyatt, explained their stance:

> *"If randomized controlled trials are to provide unbiased assessments of treatment efficacy, investigators must apply the intention-to-treat principle. To improve the applicability of study results to individual patients, investigators should improve study design to ensure protocol adherence with minimal loss to follow-up. Finally, loss to follow-up can result in exactly the same sort of bias as a per protocol analysis. Therefore, if there is significant loss to follow-up, statements that investigators conducted an "intention-to-treat analysis" generally provide little reassurance."* – pg 1341

Montori and Guyatt warned that applying the principle of ITT to those for whom follow-up information is available does not prevent bias introduced by large proportions of LTFU. Moreover, they advised that imputation strategies also can not protect trials from the bias that result from LTFU. Bubbar and Kreder[55] hold a similar position:

> *"As can be seen, an intention-to-treat approach is not a remedy for unsound design or incomplete follow-up… intention-to-treat analysis compares study groups in terms of the treatment to which they were randomly allocated, regardless of the treatment they actually received… non compliance should be kept to a minimum through the study design, as intention-to-treat analysis cannot redeem poor*

quality data resulting from inadequate design or implementation” – pg 2099

III. DEFINITION ‘ITT INVOLVES SPECIFIC STRATEGY FOR LTFU’:

ITT implies a specific way of handling participants who were LTFU. Twenty-eight (78%) out of 36 articles discussed this definition of ITT. Specific strategies mentioned for handling LTFU were divided into 10 categories.

‘ITT INVOLVES SPECIFIC STRATEGY FOR LTFU’ – TEN CATEGORIES

The section which follows will present comments and summary statistics from the 28 articles which mentioned the definition ‘ITT involves specific strategy for LTFU’. Specific strategy(ies) mentioned for dealing with LTFU fall under 10 categories (as shown in **Figure 2)**. Nine of these categories represent imputation strategies for LTFU and one strategy excludes those LTFU from the analysis (CCA). The three most frequently mentioned strategies for dealing with LTFU were CCA (mentioned in 16 articles), last outcome carried forward (LOCF) (mentioned in 14 articles), and sensitivity analysis (mentioned in 14 articles). Comments from these three strategies will be the focus of this section. Another six imputation strategies were categorized as: worst case scenario (mentioned in nine articles), best case scenario (mentioned in six articles), all had outcome

(mentioned in eight articles), all had no outcome (mentioned in five articles), use of available data to impute (mentioned in 13 articles), and multiple imputation (mentioned in three articles). Although authors frequently mentioned these six strategies, this thesis will not focus on comments from these categories. The tenth category, 'other strategies' (mentioned in 11 articles) contained imputation methods which did not fall under any of the nine previously mentioned categories. Approaches mentioned in the 'other strategies' category were unique but relatively infrequently mentioned. Out of interest for novel imputation strategies, this thesis also summarizes the strategies mentioned in these 11 articles.

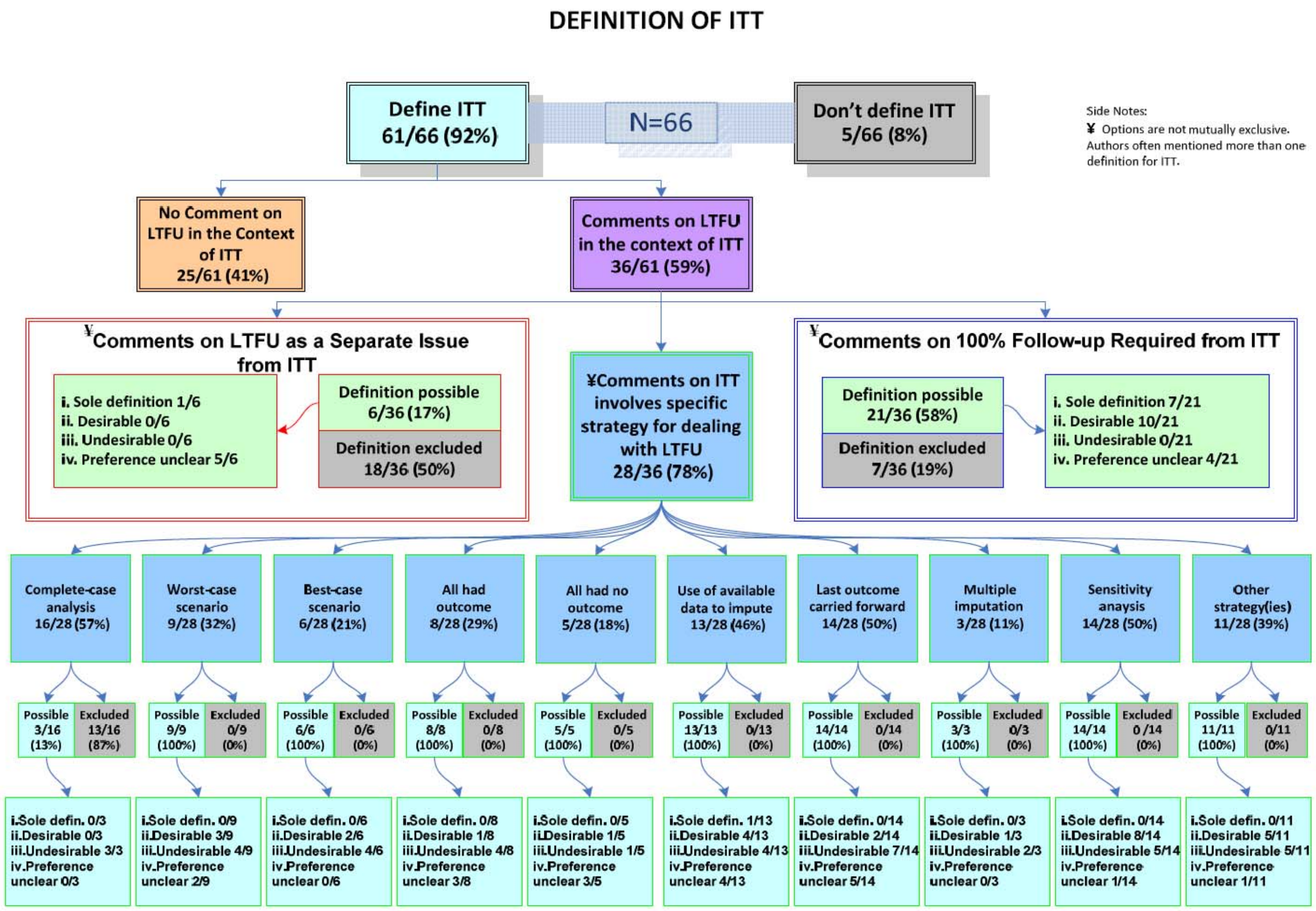

Figure 2 Definition of ITT (See Appendix G for the full size of Figure 2)

‘ITT INVOLVES SPECIFIC STRATEGY FOR LTFU’ – SUMMARY INFORMATION

The 28 authors who mentioned a specific strategy for dealing with LTFU which was acceptable under ITT, on average mentioned three [IQ, 2-4] separate strategies. Of the 28 articles, four articles suggested imputation but mentioned no specific strategy, three (11%) articles mentioned one strategy, four (14%) articles mentioned two, eight (29%) articles mentioned three, two (7%) articles mentioned four, and seven (25%) articles mentioned five or more strategies for handling LTFU under ITT (See **Table 8**).

No. of Specific Strategies for LTFU Mentioned Under ITT	No. of Articles (%)
0	14% (4)
1	11% (3)
2	14% (4)
3	29% (8)
4	7% (2)
5 or more	25% (7)
Total	28

Table 8 The articles (n=28) which mentioned the definition ‘ITT involves specific strategy for LTFU’ and the number of specific strategies suggested for handling LTFU

Of the 28 articles which endorsed the 'ITT involves a specific strategy for dealing LTFU' definition, 21 had more than one specific strategy as 'possible' under ITT. **Table 9** shows the method of handling LTFU that each of the 28 articles chose with some articles suggesting more than ten strategies. Unnebrink and Windeler (2001)[80], lists several methods of dealing with missing data that are valid ITT strategies:

> *"We examined a total of 14 ad hoc strategies for dealing with missing values. These can be roughly classified into numerical imputational strategies (last observation carried forward (LOCF), mean and regression based methods) and non-parametric strategies (rank and dichotomization based methods).* – Pg 3936

Although they mentioned more imputation strategies than any other article in this review, they did not mention either the 'ITT and LTFU are separate issues' or the 'full-follow-up required' definition. In other words, 'ITT involves specific strategy for LTFU' was their sole definition.

Author	Specific Strategy for Dealing with LTFU									
	CCA	WC	BC	All Had Outcome	All No Outcome	Using Available Data	LOCF	MI	SA	*Other
[52]Altman	X									
[53]Bailey[+]										
[54]Borm						X				
[55]Bubbar		X	X						X	
[56]D'Agostino							X	X	X	
[58]Furukawa				X	X				X	
[59]Gibaldi[+]										
[60]Gravel		X	X	X	X	X	X	X	X	X
[61]Green[+]										
[62]Hollis		X	X				X		X	X
[63]Hollis		X	X	X	X	X	X		X	
[64]Irvine				X			X		X	
[65]Kleinman	X					X	X			X
[67]Kruse	X	X	X			X			X	
[68]Lewis						X				X
[71]Nich				X		X	X			
[72]Porta									X	
[73]Sabin				X		X	X		X	
[74]Sato	X								X	X
[75]Shao							X			X
[76]Soares		X		X		X	X			X
[77]Streiner		X					X			X
[80]Unnebrink		X	X	X	X	X	X		X	X
[82]White[+]										
[83]Whittaker							X		X	X
[84]Wiens						X	X			
[85]Winnock		X			X					
[86]Wright						X		X	X	X

Table 9 The authors (n=28) who argued 'ITT involves specific strategy for LTFU'

[+] Four articles explicitly endorsed the "ITT involves a specific strategy for dealing LTFU" definition but did not mention a particular imputation

* Authors could suggest more than strategy under the 'Other' strategies category

- CCA = complete case analysis, WC = worst case scenario, BC = best case scenario, LOCF = last outcome carried forward, MI = multiple imputation strategy, and SA = sensitivity analysis

ITT was the complete case analysis (CCA). As seen in **Figure 2**, of the 16 articles

which mentioned CCA, three articles (19%) thought it was a possible definition for ITT. Of those three articles, none believed CCA was the sole definition, none argued it was desirable, three argued it was undesirable, and none had no preference. Out of the 16 articles which commented on CCA, 13 (81%) articles specifically excluded CCA as a valid strategy under ITT. A good example of an article which specifically excluded CCA is Unnebrink and Windeler (2001)[80]. These authors explicitly stated CCA is “the Non-ITT strategy” and listed several methods of dealing with missing data which are valid ITT strategies:

> *“We examined a total of 14 ad hoc strategies for dealing with missing values... We included CCA as the non-ITT strategy for reference purposes only.”* – Pg 3936

Soares also excluded CCA[76]:

> *“This method violates the principle of intention to treat and leads to bias unless data is missing at random, that is, unless absence of a response is independent of the outcome”* – pg 1196

Three articles argued CCA was possible but an undesirable method of handling LTFU under ITT. One article which held this argument was conducted by Kruse et al.[67] This article systematically reviewed RCTs (n=100) which reported use of ITT for their primary analysis. More than half of these RCTs excluded

patients from their analysis. Although Kruse et al. article endorsed full follow-up as the 'hallmark of ITT', it admitted that this is not how ITT is commonly understood by trialists. In their sample, more than one quarter of RCTs excluded randomized patients from the analysis due to LTFU. And in more than 10% of RCTs they could not determine who was in the ITT group.

Similar to Kruse, the 2001 CONSORT statement mentions CCA as possible under ITT. Altman et al.[52] states:

> *"It is common for some patients not to complete a study – they may drop out or be withdrawn from active treatment – and thus are not assessed at the end. Although those participants cannot be included in the analysis, it is customary still to refer to analysis of all available participants as an intention-to-treat analysis."* – pg 681

In addition to allowing for a CCA, the CONSORT statement mentioned all three definitions as possible under ITT.

As shown in **Figure 2**, of the 28 articles which mentioned 'ITT involves specific strategy for LTFU' definition, 14 (56%) mentioned last outcome carried forward (LOCF). All 14 argued that a LOCF for LTFU was a possible definition for ITT. This made LOCF the most commonly mentioned 'possible' imputation

strategy for continuous data in this review. Of the 14, none argued it was the sole, two (14%) argued desirable, seven (50%) argued undesirable, five (36%) had unclear preference, and none argued to exclude this definition.

An article by Shao and Zhong (2003)[75] is an example of an article which endorsed the LOCF under ITT. Shao and Zhong examined in detail the statistical implications of imputation of missing data using LOCF under ITT. Definition 'ITT involves specific strategy for LTFU' was their sole definition. Unnebrink and Windeler[8] was the only other article which endorsed LOCF.

Soares and Carneiro (2002)[76] argued that a LOCF approach (and other imputation strategies) was acceptable but undesirable under ITT. Instead, they advocated for the 'full-follow-up required' definition:

> *"Other methods used to solve this problem include… carry forward of last observation response, explicit allocation of poor outcome, implicit assumption of good or poor outcome, and use of the group average… However, no imputation method can provide an unbiased assessment of the treatment effect unless the assumptions about the missing data are valid… Full application of intention-to-treat is possible only when complete outcome data are available for all randomized subjects"* – pg 1196-1197

Asserting that imputation using LOCF was undesirable while supporting the 'full follow-up required' definition was a position held by five other authors: Gravel et al. (2007)[60], Hollis et al. (2002)[63], Nich and Carroll (2002)[71], Whittaker et al. (2006)[82] and Weins et al. (2007).[84]

We defined sensitivity analysis as running more than one of any imputation strategy for LTFU (see **Table 1** for some of the possible imputation strategies). Of the 14 articles which mentioned a sensitivity analysis, all 14 argued that a sensitivity analysis for those LTFU was a possible definition for ITT. Of the 14, none argued it was the sole definition, eight (57%) argued it was a desirable definition, five (36%) argued it was an undesirable definition, and one (7%) had unclear preferences. None argued to exclude this definition. More articles deemed sensitivity analysis a 'desirable' method of handling those LTFU under ITT than any other imputation strategy which we identified. Bubbar and Kreder[55], was one of these eight articles recommending its use and identified an advantage of performing a sensitivity analysis:

> *"If a sensitivity analysis was performed in a trial, clinicians can decide for themselves if the number of subjects lost to follow-up is excessive".* – pg 2099

Both Sato[74] and Porta[72] advocated for a sensitivity analysis as a way to handle nonrandom missing data. Porta stated:

> *"To manage nonrandom missingness, sensitivity analysis considering the missing data mechanism should be routinely performed to explore influence of nonignorable missing data in the validity of the results and also to make explicit the underlying assumption behind any missing data approach"* – pg 667

Of the 28 authors who commented on a specific strategy for handling LTFU in relation to ITT, 11 (39%) suggested and created 'other' unique imputation strategies (See **Table 6**). For example, Streiner[77] favored 'growth curve' analysis as a novel and advanced approach of handling LTFU:

> *"Within the last decade, a new approach has emerged, usually called Growth Curve analysis. As the name implies, it calculates the trajectory of change over time for each person based on whatever data are available (as long as there are at least 2 measurements), and then estimates (or "imputes") what the missing data would be if the person followed on the same trajectory. This also means that it can estimate a value that is missing in the middle because the person skipped 1 or 2 evaluation sessions, but actually completed the study. This method requires considerable statistical sophistication…"* – pg 70

The 'ITT involves specific strategy for LTFU' was Streiner's sole definition. Other than specifically recommending growth curve analysis, the author mentioned LOCF and worst case scenario to address missing data.

In addition to suggesting LOCF and worst case/best case scenario, Unnebrink and Windeler described and recommended some distinct imputation strategies. For example, the authors recommended imputation of the mean of ‘other’ groups and regression based on observed patients on placebo. In total, the authors suggest 14 ‘ad hoc’ strategies for dealing with missing values, six of which were captured in our identified categories (See **Figure 2**) and eight were captured under the ‘other strategies’ category (See **Table 10)**.

Author, Year	'Other strategies' for dealing with LTFU under ITT
[60]Gravel, 2007	i. Regression models
[62]Hollis, 2002	i. Pattern mixture approach: special case of stratified analysis which stratifies the data by pattern of missing data is performed.
[65]Kleinman, 1998	i. Bayesian Framework to fit linear model
[68]Lewis, 1993	i. Define a new outcome measure not dependant on partially missing values.
[74]Sato, 2001	i. Pattern mixture approach
[75]Shao, 2003	i. Last observation analysis (different from LOCF)
[76]Soares, 2002	i. Implicit assumptions of good or poor outcome
[77]Streiner, 2001	i. Growth curve:
[83]Whittaker, 2006	i. Group mean
[86]Wright, 2003	i. Mean Imputation<br>ii. Likelihood based strategies<br>iii. Regression analysis<br>iv. Hot-deck imputation<br>v. Applying standard statistics tests to rank data
[80]Unnebrink, 2001	i. Imputation of the mean of own group<br>ii. Imputation of mean of other group<br>iii. Regression based on observed patients of placebo group<br>iv. Regression based on observed patients of own group<br>v. Regression based on observed patients of other group<br>vi. Minimax-regression<br>vii. Ranking strategy according to Gould<br>viii. Ranking strategy according to Senn

Table 10: Summary of the 'other strategies' mentioned by authors for handling LTFU under ITT

Of the 61 authors who gave a basic definition for ITT, approximately two-thirds (38) used the word '*All*' such as in the phrase 'all participants randomized were analyzed' and one-third (23) did not use the term 'All' such as in the phrase 'participants were analyzed as randomized'. On the surface, use of the term 'All' could be interpreted as requiring full follow-up. Of the 36 articles which commented on LTFU in the context of ITT, 26 (72%) used the term 'all' and 10 (28%) did not use 'All' in their definition of ITT. Since these articles discussed LTFU in relation to ITT, we were able to determine how these authors actually defined ITT.

As it turns out, of the 26 articles which used the term 'all', 16 (62%) mentioned the definition 'full follow-up required', three (12%) mentioned the definition 'ITT is a separate issue from LTFU', and 21 (81%) mentioned the definition 'ITT involves specific strategy for LTFU'. These proportions were very similar to the proportions of the 10 articles that did not use the term 'All'.

As shown in **Figure 2**, 25 of the 61 articles defined ITT but did not comment on LTFU. Of these 25 articles, 12 (48%) mentioned the term 'all'. Based on data from the 36 articles, one could make the assumption that most of these 12 articles defined ITT as involving a specific strategy for LTFU or requiring

full follow up. See **Table 11** for examples of using 'All' in the basic definition of ITT.

**Author**	**Basic definition of ITT provided**	**Comments on LTFU in the Context of ITT: Definition Endorsed**
Wright86	"…data are analyzed according to the group to which participants were randomized…" – pg 833	Endorsed full follow up required definition, mentioned nine possible but undesirable imputation strategies for handling LTFU, and regarded CCA as invalid.
Winnock85	"…patients must be analyzed as part of the treatment group to which they were originally assigned even if they failed to receive the intended treatment" – pg 1014	Endorsed the 'worst-case scenario' strategy under the "ITT involves specific strategy for LTFU" definition, and mentioned the 'all had outcome' strategy as a possible but undesirable imputation.
Yusuf87	"Analysis that preserves the benefits of randomization by including all randomized patients based on their original allocation" - pg 2078	Endorses full follow-up as the only valid definition for ITT

Table 11: Examples of the basic definition provided by articles which commented on LTFU in the context of ITT. Yusuf uses 'All' in the basic definition while Wright and Winnock do not.

AUTHORS HOLDING ITT AS THE GOLD STANDARD

Many articles declared ITT the 'gold standard' such as the following quote from D'Agostino[56]:

> *"Present-day thinking favors the ITT sample for the primary data analysis. It is the only data set that preserves randomization and, some argue, the only one that prevents biases and justifies statistical analysis"* – pg 22

Nich[71] states:

> *"Although intent-to-treat analysis is the undisputed 'gold standard' in reporting results of clinical trials, true intent-to-treat analyses are rare."* – pg 122

Another example is this quote from Streiner[77]:

> *"It is generally accepted, therefore, that the most clinically informative, as well as the most statistically robust, method of analysis is an intention to treat (ITT) analysis…"*– pg 70

There were a number of articles that acknowledged the ambiguity in the ITT definition. Soares and Carneiro[76] expressed this viewpoint in the following statement:

> *"There is considerable ambiguity in the concept of "intention-to-treat analysis", which can mean different things to different authors"* – pg 1195

Altman et al.,[52] in the 2001 CONSORT statement also suggests the following:

> *"This intention to treat strategy is not always straightforward to implement."*- pg 681

STRENGTHS AND LIMITATIONS

Our study is limited in that we selected articles from within MEDLINE®, which mainly focuses on disease and treatment and may not necessarily be a 'methods database'. This might have resulted in a lower proportion of statistical and methods articles compared to other databases. Using multiple databases would have increased the generalizability of our results. If multiple databases were used and more papers were extracted there is a possibility that a clearer picture may have emerged with more data. On the other hand, if more papers were identified and more databases searched there would likely be even more disagreement on what is meant by ITT, suggesting our findings are if anything conservative.

One strength of this review is that about half of our articles were derived from methods and statistics journals and on average, articles contained

approximately four pages [IQ, 1-6] discussing ITT. Thus, our review did capture a variety of different journal types for external validity. It was beneficial that the extracted articles contained an in-depth discussion of ITT in order to discover relevant and unique perspectives. Moreover, with only one database it was more difficult to find variations in the ITT definitions making our findings more conservative.

Another strength of this review is the method in which the data extraction form was developed. As mentioned above, in contrast to approaches of systematically applying a set of pre-existing questions, our approach was data-derived so that the data extraction was adjusted iteratively in response to the data itself, and systematically applied as the study progressed. This meant that modifications were made to the data extraction form throughout the study to best ‘fit the data’ and to accommodate new insights from the data. This method is often called qualitative content analysis and is the strategy of choice in qualitative descriptive studies.[91] Furthermore, all articles were screened and abstracted in duplicate and discrepancies were adjudicated by experienced research methodologist.

CHAPTER 4: DISCUSSION

RCTs are considered the top of the hierarchy of study designs addressing causal issues because they eliminate many sources of bias which befall other experimental designs. Therefore the design, execution, and analysis of RCTs should be methodologically sound. Just as there are inherent expectations that come from the use of the term RCT, there are also some expectations that readers of these articles may justifiably make about the terms contained within, such as ITT. The most striking finding of our systematic review is that there is <u>no</u> meaningful consensus among authors of methodology articles on the definition of ITT.

This finding is of great concern because the ITT principle has been nearly universally recommended as the analysis of choice – the "gold standard" – for clinical trials by health research methodology experts and authorities. Research methodologists, statisticians, governing bodies and recognized research groups such as the 2001 CONSORT (Consolidated Standards of Reporting Trials) guidelines, Cochrane Reviews, the FDA, the Nordic Council on Medicine in Europe, and the ASA recommend using the ITT analysis as the primary analysis in RCTs.[11-14, 88-90]

Of the articles reviewed, six did not define ITT despite devoting numerous pages discussing it. There are several possible reasons for this, but one likely reason is that the authors simply assumed a certain definition of ITT. Similarly, not all articles which defined ITT discussed the issue of LTFU. More specifically, over one-third of articles (25 of 61) did not explicitly comment on LTFU in the context of ITT – yet they discussed ITT for an average of 2 pages. It should be noted that some of these 25 articles may have considered ITT and LTFU are separate issues without explicitly saying so.

Despite a lack of consensus regarding LTFU among authors of methods articles, many authors defined or even advocated certain strategies. Of the authors who considered ITT and LTFU to be related issues, authors endorsed either imputing (i.e. making up data) and/or requiring full follow-up.

CCA, interestingly, was almost unanimously disliked as a method of handling LTFU under ITT by authors of methodology articles. Sixteen out of 28 articles mentioned CCA as a method of dealing with LTFU of which 13 specifically exclude it. The three articles which thought CCA was a possibility under ITT also thought it was an undesirable strategy. Clinical trialists, on the other hand, often use CCA as a method of conducting what they describe as an ITT. A recent review by Gravel et al. showed that CCA was used in about half of RCTs reporting ITT.[60]

It should be noted that in our study, 81% of methods articles which used CCA to impute data describe it as a “non-ITT analysis”. Nevertheless, this discrepancy between the analyses that methodologists recommend and the strategies that trialists actually use highlights the current troubling state of the ITT principle in RCTs.

Previous reviews of the use of ITT in RCTs have concluded that ITT is often ‘misused’ or incorrectly applied.[16,17,19,67,76] Such a conclusion assumes that there exists a correct definition of ITT. If there were a ‘correct’ definition of ITT, one would expect that definition to be uniformly or nearly uniformly applied in clinical trials and certainly standard among methodological articles. We have found that the assumption of a ‘correct’ definition of ITT is invalid.

While other reviewers have shown that the concept of ITT is inconsistently applied in clinical trials, the current review is the first to demonstrate that this ambiguity in the ITT definition extends to methods articles as well. Not only did certain articles provide differing definitions, but 17 out of 36 articles (47%) provided more than one definition of ITT within the same article.

Similar to our research group, authors of methodology articles generally agreed on the ‘basic’ definition of ITT. This definition essentially states

that for those participants in whom the outcome is known, one should perform analyses using the groups to which the participants were originally randomized. Clinical trialists, on the other hand, do not hold to this same 'basic' definition quite as uniformly. The latest systematic reviews showed that authors of RCTs clearly violated this major component of ITT more than 7% of the time[16,17,19,67,76]. In at least another 7%, it was not clear whether or not they followed the basic principle. Thus, several reviews have concluded that trials reporting use of 'ITT analysis' provide little reassurance that participants were actually 'analyzed as randomized'. Similarly, this review shows that use of phrases like 'all participants were analyzed in the groups to which they were randomized' in the basic definition of ITT cannot be taken to mean that full follow-up was achieved for every participants. In fact, in our review, 80% of authors that used such phrases suggested imputing data for those LTFU was possible under ITT.

While discrepancies between methodologists and trialists have a potentially profound negative impact on the way LTFU is managed in RCTs, the lack of agreement between methodologists and statisticians on this subject is perhaps an even greater problem. It could be argued that if a 'correct' definition of ITT exists, than trialists are simply guilty of 'incorrect application' of a principle. This could be remedied by strong methodology position articles. However, it must be emphasized that since the "experts" in this area, namely the

methodologists and the statisticians, do not agree on a single ITT definition, the problem is considerably more insidious.

A WAY FORWARD

The results of the current research, including a systematic review of the use of ITT in methodology articles, revealed that there is significant ambiguity in the field as a whole. If ITT is to remain among the terms used in clinical trials, it seems that one of the following adjustments must be made to the definition and to its practical application.

The **first option** is to keep the term ITT, but for editors to insist that authors define it clearly. This approach seems reasonable initially, since scientific articles often define terms within the context of the study presented. However, we have identified three different ways in which methodologists may define ITT. This solution also opens the door for new definitions of ITT to emerge with each new published article. The sheer number of possible ITT definitions makes this solution to the problem too cumbersome for readers. How can one compare trials when the definition of ITT is so malleable and varied—and so dependent on the individual researcher's conception of ITT?

Perhaps more importantly, adopting this approach will make future meta-analyses of ITT virtually impossible. Any review using the search term ITT

must separate and characterize each ITT definition individually. This necessarily limits any meaningful, statistical comparisons that one could make with that data. By extension, conclusions drawn from those analyses would be severely limited or flawed. Incidentally, this approach is very similar to the current state of ITT except that some clinical trialists do not define ITT at all.

The **second option** is to keep the term ITT but look to the research community to stipulate one of the three ITT definitions – assuming they can agree on one. This option assumes the research community can reach a consensus on which definition of ITT to implement. This also raises the issue of how to deal with RCTs that report ITT yet do not apply the newly agreed upon definition. Would these results now be rendered invalid?

Even so, there are certainly instances in which some groups have attempted to do this by calling 100% follow up the 'True', 'Classic', or 'Ideal' ITT.[62,63,67,71,81] This definition requires that a study labeled ITT must have 100% follow up. These authors see ITT as an ideal only achieved by superlative design where not a single randomized participants had an unknown outcome. Approximately 18% (7 out of 39) of methodologists who commented on LTFU held this version of ITT as the sole definition. Clinical trialists are likely to balk at this definition since it is quite difficult to achieve in common practice. When one

considers that over 50% of RCTs claiming they conducted ITT experienced LTFU, this solution would exclude most trialists from being able to claim conduct of an ITT.[16,17,19,67,76].

A second definition authors held was that ITT is a completely separate issue from LTFU. Only a fraction of articles advocated this definition (6 of the 36 who mentioned the separation as possible; 6 of a total of the 66 articles reviewed). However, this stance, endorsed as the sole definition by Montori and Guyatt,[69] does clear up several methodological issues. The first issue which it solves is that it preserves the generally agreed upon definition of ITT which states if outcome information is available for study participants, they remain in their randomized group. Since approximately 7% of RCTs violate the basic principle of ITT (don't analyze as randomized for participants followed), applying ITT in this way would encompass over 90% of published studies.[16,17,19,67,76] Participants with an unknown outcome, i.e. patients LTFU, would be considered separately.

By separating the two issues, the focus is shifted away from terminology. LTFU threatens the quality of an RCT no matter what imputation method is used. However, the authors that advocate for the full follow up definition would argue that one drawback of separating ITT and LTFU is a decreased incentive for

trialists to attain full follow-up. In other words, forcing RCTs to have full follow-up in order to be considered 'true' ITT, encourages better follow-up in trials. Separation of the terms provides an 'out' for studies with large amounts of lost data.

The third definition that could be advocated is that ITT requires a specific strategy for handling LTFU. In recent literature reviews, at least 60% of RCTs apply ITT in this way.[16,17,19,67,76] In our review, of the 36 articles that commented on LTFU most authors (78%) thought this definition was a possible definition. The two imputation strategies most mentioned for dealing with LTFU were the LOCF method (50%) and sensitivity analysis (50%). More methodologists recommended a sensitivity analysis as a method of handling those LTFU under ITT. Of the 14 that mentioned sensitivity analysis, more than half endorsed its use in ITT. Yet, in reviews of RCTs, use of a sensitivity analysis was almost nonexistent (1%).

In our review, we noted various other approaches to deal with missing data, but all are imperfect. No imputation method can remedy a poorly designed trial with a large number of LTFU, especially when LTFU is associated with the outcome of interest. Thus, if the research community were to agree on this definition of ITT, it would be important for RCTs to perform a sensitivity

analysis. Readers deserve to know the extent to which different assumptions about LTFU alter a trial's conclusion.

If the trialists applying ITT, the methodological experts on research design, the 'authorities' on ITT such as Bradford Hill (see Appendix F), and research organizations all have differing definitions, what is the future for ITT? Can the term ITT be saved?

Some organizations like Cochrane and CONSORT have made changes recently to their definition of ITT suggesting that the field is beginning to acknowledge the problem. Specifically, the most recent CONSORT statement no longer advocates for the use of ITT.[92] This review, considered alongside previous reviews of RCTs, demonstrates that simply stating a study employed ITT is useless at best and misleading and dangerous at worst. ITT is a term trialists use, and methodologists advocate, as an indicator of the validity of RCTs. Moreover, the term's status as a 'gold standard' in RCTs risks inaccurate inferences about the validity of the studies on which clinicians base their clinical practice.

Thus, a **third option** is for the research community to replace the term ITT with two clear statements about 1) What was done with participants who were followed, and 2) What was done with participants LTFU. This option provides an elegant solution to the problems examined in this thesis.

Furthermore, this review proposes the term *analysis without bias* in which full follow-up is achieved and one analyzes all participants in the group to which they were originally randomized. Under an analysis without bias no participants are LTFU. However, an *analysis without bias* would allow for post-randomization exclusions of certain groups only if they introduce no bias. An example of this is the exclusion of ineligible patients defined a priori and based on information obtained at baseline. The term *analysis without bias* acknowledges and distinguishes RCTs that have accomplished full follow-up and provides an alternative to the ambiguous term ITT.

Our study has showed ambiguity in the traditional ways of describing ITT. Given this inconsistency, ITT is falling short of achieving the objective of explicit research communication. This is not the first time a popular research term has been shown to potentially do more harm than benefit. Devereaux et al., (2000)[93] showed that physician interpretations and textbook definitions of 'blinding' terminology (single, double, and triple) varied greatly. Devereaux as well as CONSORT subsequently called for the replacement of the ambiguous 'blinding' terminology with use of explicit statements to describe the 'blinding' status of specific groups. Similarly, the current 2010 CONSORT statement recommended dropping the term ITT in favour of a clear description of exactly who was included in each analysis. This thesis advocates replacing the current

term ITT with transparent and explicit reporting of the handling of participants followed and participants LTFU. This stance addresses the various methodological issues described in this thesis.

Endnotes

[1] Akl EA, Briel M, You JJ, Lamontagne F, *et al*;" LOST to follow-up Information in Trials (LOST-IT): a protocol on the potential impact."; Trials;10, (2009); 10-40
[2] http://dx.doi.org/10.1002/0470011815.b2a01030 (accessed 9/10) (as per Personal Communication, S. Walter, 2/18/2008)
[3] Personal communication, S.W., 02/04/2008 email
[4] Personal communication, G.G., 02/05/2008 email
[5] Personal Communication with D Sackett (add date)
[6] Bell JA (1941). Pertussis prophylaxis with two doses of alum-precipitated vaccine. Public Health Reports 56:1535-1546.
[7] A. Bradford Hill, Principles of Medical Statistics, 7th edition, Oxford University Press, 1961, page 258.
[8] Unnebrink K, Windeler J. "Intention-to-treat: methods for dealing with missing values in clinical trials of progressively deteriorating diseases." Statistics in Medicine (2001);20(24):3931-46.
[9] The European Agency for the Evaluation of Medicinal Products. ICH E9 – Statistical Principles for Clinical Trials: Note for guidance on statistical principles for clinical trials. CPMP=ICH=363=96, Step 5, 1998.
[10] http://www.cochrane-net.org/openlearning/HTML/mod14-4.htm Cochrane Collaboration Handbook (accessed 9/10)
[11] Food and Drug Administration (1988). Guideline for the format and content of the clinical and statistical sections of new drug applications. FDA, US Department of Health and Human Services; Rockville, MA, USA
[12] Saadatmand R, Stinson FS, Grant BF, Dufour MC. "Surveillance report 45: liver cirrhosis mortality in the United States, 1970-94." Washington, DC: US Department of Health and Human Services, National Institutes of Health, 1997.
[13] Nordic Council on Medicine (1989). Good clinical trial practice; Nordic Guidelines, NLN Publication No. 28, Uppsala, Sweden.
[14] Altman,DG, Schulz, KF, Moher, D., Egger, M., Davidoff, F. *et al;* "The Revised CONSORT Statement for Reporting Randomized Trials: Explanation and Elaboration", Annals of Internal Medicine, 2001, 134:663-694.
[15] Sabin CA, Lepri AC, Phillips AN." A practical guide to applying the intention-to-treat principle to clinical trials in HIV infection." HIV Clinical Trials (2000)**;1**(2):31-8.
[16] Hollis S, Campbell F: "What is meant by intention to treat analysis? Survey of published randomised controlled trials". BMJ (1999), 319:670-674.
[17] Gravel, J., L. Opatrny, *et al*. "The intention-to-treat approach in randomized controlled trials: are authors saying what they do and doing what they say? " Clinical Trials,. (2007), 4(4): 350-6.

---

[18] Wood AM, White IR, Thompson SG: “Are missing outcome data adequately handled? A review of published randomized controlled trials in major medical journals.” Clinical Trials (2004), **1**:368-376.
[19] Ruiz-Canela M. Martinez-Gonzalez MA. de Irala-Estevez J. “Intention to treat analysis is related to methodological quality”.[comment]. [Comment. Letter] BMJ(2000). 320(7240):1007-8.
[20] Gross D, Fogg L. “A critical analysis of the intent-to-treat principle in prevention research.” The Journal of Primary Prevention (2004) 25:475–489
[21] Landis JR, Koch GG, Landis JR, Koch GG: “The measurement of observer agreement for categorical data.” Biometrics (1977) 33:159-17
[22] Branson M, Whitehead J. “A score test for binary data with patient non-compliance.” Statistics in Medicine (2003);22(20):3115-32.
[23] Farrington CP. “Intention-to-treat analyses in clinical trials and cohort studies.”[comment]. International Journal of Epidemiology (1993);22(3):566
[24] Mittlbock M, Whitehead J. “The interpretation of clinical trials of immediate versus delayed therapy. Lifetime Data Analysis “(1998);4(3):253-63.
[25] Ruiz-Canela M. Martinez-Gonzalez MA. de Irala-Estevez J.” Intention to treat analysis is related to methodological quality.”[comment]. [Comment. Letter] BMJ (2000) 320(7240):1007-8.
[26] Witte S., Victor N., Some Problems with the Investigation of Noninferiority in Meta-analysis, Methods Information in Statistics, (2004),43:470-4.
[27] Akobeng AK. Understanding randomised controlled trials. Archives of Disease in Childhood 2005;90(8):840-4
[28] Bentzen SM. Towards evidence based radiation oncology: improving the design, analysis, and reporting of clinical outcome studies in radiotherapy.[see comment].
[29] Bentzen SM. Towards evidence based radiation oncology: improving the design, analysis, and reporting of clinical outcome studies in radiotherapy.[see comment].
[30] Blackwelder WC. Current issues in clinical equivalence trials. Journal of Dental Research 2004;83 Spec No C:C113-5
[31] Blume J, Peipert JF. Randomization in controlled clinical trials: why the flip of a coin is so important. Journal of the American Association of Gynecologic Laparoscopists 2004;11(3):320-5
[32] Chene G, Morlat P, Leport C, Hafner R, Dequae L, Charreau I, et al. Intention-to-treat vs. on-treatment analyses of clinical trial data: experience from a study of pyrimethamine in the primary prophylaxis of toxoplasmosis in HIV-infected patients. ANRS 005/ACTG 154 Trial Group. Controlled Clinical Trials 1998;19(3):233-48
[33] Daya S. Methodological issues in infertility research. Best Practice & Research in Clinical Obstetrics & Gynaecology 2006;20(6):779-97.
[34] Fergusson D, Aaron SD, Guyatt G, Hebert P. Post-randomisation exclusions: the intention to treat principle and excluding patients from analysis. BMJ 2002;325(7365):652-4.

---

[35] Freedman DA. Statistical models for causation: what inferential leverage do they provide? Evaluation Review 2006;30(6):691-713
[36] Glasziou PP. Meta-analysis adjusting for compliance: the example of screening for breast cancer. Journal of Clinical Epidemiology 1992;45(11):1251-6
[37] Goetghebeur E, Loeys T. Beyond intention to treat. Epidemiologic Reviews 2002;24(1):85-90.
[38] Sorensen HT, Lash TL, Rothman KJ. Beyond randomized controlled trials: a critical comparison of trials with nonrandomized studies. Hepatology 2006;44(5):1075-82.
[39] Jones B, Jarvis P, Lewis JA, Ebbutt AF. Trials to assess equivalence: the importance of rigorous methods.[see comment][erratum appears in BMJ 1996 Aug 31;313(7056):550]. BMJ 1996;313(7048):36-9
[40] Law Matthew G., Kaldor John M., Survival Analyses of Randomized Clinical Trials Adjusted For Patients Who Switch Treatments, Statistics In Medicine, (1996),15, 2069-2076.
[41] Loeys T, Goetghebeur E. A causal proportional hazards estimator for the effect of treatment actually received in a randomized trial with all-or-nothing compliance. Biometrics 2003;59(1):100-5
[42] Lui K-J. Interval estimation of the risk difference in non-compliance randomized trials with repeated binary measurements. Statistics in Medicine 2007;26(16):3140-56.
[43] Mahaniah KJ. Rao G. Intention-to-treat analysis: protecting the integrity of randomization. [Journal Article] Journal of Family Practice. 53(8):644, 2004 Aug.
[44] Nagelkerke N, Fidler V, Bernsen R, Borgdorff M. Estimating treatment effects in randomized clinical trials in the presence of non-compliance.[erratum appears in Stat Med 2001 Mar 30;20(6):982]. Statistics in Medicine 2000;19(14):1849-64.
[45] Newcombe R.G., Explanatory and Pragmatic Estimates of the Treatment Effect When Deviations From Allocated Treatment Occur, Statistics In Medicine, (1988), 7. 1179-1186
[46] Peace Karl E., Carter Hans Jr., Exposure Analysis of Dichotomous Response Measures in Long-Term Studies, Journal of Biopharmaceutical Statistics, (1993), 3(1), 129-140
[47] Sheiner LB, Rubin DB. Intention-to-treat analysis and the goals of clinical trials. Clinical Pharmacology & Therapeutics 1995;57(1):6-15
[48] Schoenfeld PS. Evidence-based medicine in practice: applying intention-to-treat analysis and perprotocol analysis. [Case Reports. Journal Article] American Journal of Gastroenterology. 100(1):3-4, 2005 Jan
[49] Thoma A. Challenges in creating a good randomized controlled trial in hand surgery. Clinics in Plastic Surgery 2005;32(4):563-73
[50] Viscoli C, Bruzzi P, Glauser M. An approach to the design and implementation of clinical trials of empirical antibiotic therapy in febrile and neutropenic cancer patients. European Journal of Cancer 1995;31A(12):2013-22.
[51] Weinstein, Gerald S., Levin, Bruce, Effect of Crossover on the Statistical Power of randomized studies, Annals of Thorac Surgery, 1998 48, 190-3.

52. Douglas G. Altman, DSc; Kenneth F. Schulz, PhD; David Moher, MSc; Matthias Egger, MD; Frank Davidoff, MD; Diana Elbourne, PhD; Peter C. Gøtzsche, MD; and Thomas Lang, MA, The Revised CONSORT Statement for Reporting Randomized Trials: Explanation and Elaboration, Annals of Internal Medicine, 2001, 134:663-694.
53. Bailey A, Crook A, Machin D. Statistical methods for clinical trials. Blood Reviews 1994;8(2):105-12
54. Borm GF, Houben RMGJ, Welsing PMJ, Zielhuis GA. An investigation of clinical studies suggests those with multiple objectives should have at least 90% power for each endpoint. Journal of Clinical Epidemiology 2006;59(1):1-6.
55. Bubbar VK, Kreder HJ. The intention-to-treat principle: a primer for the orthopaedic surgeon. Journal of Bone & Joint Surgery - American Volume 2006;88(9):2097-9
56. D'Agostino RB, Sr., Massaro JM. New developments in medical clinical trials. Journal of Dental Research 2004;83 Spec No C:C18-24.
57. Fleiss JL. General design issues in efficacy, equivalency and superiority trials.[see comment]. Journal of Periodontal Research 1992;27(4 Pt 2):306-13; discussion 323-7.
58. Furukawa TA, Cipriani A, Barbui C, Brambilla P, Watanabe N. Imputing response rates from means and standard deviations in meta-analyses. International Clinical Psychopharmacology 2005;20(1):49-52.
59. Gibaldi Milo, Sullivan Sean, Intention-to-Treat Analysis in Randomized Trials: Who Gets Counted?, Issues in Clinical Pharmacology, 1997;37:667-672
60. Gravel J, Opatrny L, Shapiro S. The intention-to-treat approach in randomized controlled trials: are authors saying what they do and doing what they say? Clinical Trials 2007;4(4):350-6.
61. Green SB. Hypothesis testing in clinical trials. Hematology - Oncology Clinics of North America 2000;14(4):785-95.
62. Hollis S. A graphical sensitivity analysis for clinical trials with non-ignorable missing binary outcome. Statistics in Medicine 2002;21(24):3823-34.
63. Hollis S. Campbell F. What is meant by intention to treat analysis? Survey of published randomised controlled trials.[see comment]. [Review] [39 refs] [Journal Article. Review] BMJ. 319(7211):670-4, 1999 Sep 11.
64. Irvine EJ, Whitehead WE, Chey WD, Matsueda K, Shaw M, et al. Design of treatment trials for functional gastrointestinal disorders. Gastroenterology 2006;130(5):1538-51
65. Kleinman KP, Ibrahim JG, Laird NM. A Bayesian framework for intent-to-treat analysis with missing data. Biometrics 1998;54(1):265-78
66. Korhonen PA, Laird NM, Palmgren J. Correcting for non-compliance in randomized trials: an application to the ATBC Study. Statistics in Medicine 1999;18(21):2879-97
67. Kruse RL. Alper BS. Reust C. Stevermer JJ. Shannon S. Williams RH. Intention-to-treat analysis: who is in? Who is out?[erratum appears in J Fam Pract. 2002 Dec;51(12):1079.]. [Review] [12 refs] [Comparative Study. Journal Article. Review] Journal of Family Practice. 51(11):969-71, 2002 Nov.
68. Lewis J.A. Machin D. Intention to treat - who should use ITT?. British Journal of Cancer. 68:647-650, 1993.

69. Montori VM. Guyatt GH. Intention-to-treat principle. [Review] [8 refs] [Journal Article. Research Support, Non-U.S. Gov't. Review] CMAJ Canadian Medical Association Journal. 165(10):1339-41, 2001 Nov 13.
70. Newell DJ. Intention-to-treat analysis: implications for quantitative and qualitative research.[see comment]. International Journal of Epidemiology 1992;21(5):837-41
71. Nich C. Carroll KM. Intention-to-treat meets missing data: implications of alternate strategies for analyzing clinical trials data. [Comparative Study. Journal Article. Research Support, U.S. Gov't, P.H.S.] Drug & Alcohol Dependence. 68(2):121-30, 2002 Oct 1.
72. Porta N. Bonet C. Cobo E. Discordance between reported intention-to-treat and per protocol analyses. [Review] [37 refs] [Comparative Study. Journal Article. Research Support, Non-U.S. Gov't. Review] Journal of Clinical Epidemiology. 60(7):663-9, 2007 Jul.
73. Sabin CA, Lepri AC, Phillips AN. A practical guide to applying the intention-to-treat principle to clinical trials in HIV infection. HIV Clinical Trials 2000;1(2):31-8.
74. Sato T. A method for the analysis of repeated binary outcomes in randomized clinical trials with non-compliance. Statistics in Medicine 2001;20(17-18):2761-74.
75. Shao J, Zhong B. Last observation carry-forward and last observation analysis.[see comment]. Statistics in Medicine 2003;22(15):2429-41
76. Soares I. Carneiro AV. Intention-to-treat analysis in clinical trials: principles and practical importance. [Review] [14 refs] [Journal Article. Review] Revista Portuguesa de Cardiologia. 21(10):1191-8, 2002 Oct.
77. Streiner D, Geddes J. Intention to treat analysis in clinical trials when there are missing data. Evidence-Based Mental Health 2001;4(3):70-1
78. Tillmann H.C., Sharpe N., Sponer G. and Wehling M., Does Intention-to-treat analysis answer all questions in long-term mortality trials? Considerations on the basis of the ANZ trial, International Journal of Clinical Pharmacology and Therapeutics, 2001, 39, 205-212.
79. Tierney JF, Stewart LA. Investigating patient exclusion bias in meta-analysis.[see comment]. International Journal of Epidemiology 2005;34(1):79-87
80. Unnebrink K, Windeler J. Intention-to-treat: methods for dealing with missing values in clinical trials of progressively deteriorating diseases. Statistics in Medicine 2001;20(24):3931-46
81. Walter SD, Guyatt G, Montori VM, Cook R, Prasad K. A new preference-based analysis for randomized trials can estimate treatment acceptability and effect in compliant patients.[erratum appears in J Clin Epidemiol. 2007 Nov;60(11):1203]. Journal of Clinical Epidemiology 2006;59(7):685-96
82. White IR. Uses and limitations of randomization-based efficacy estimators. Statistical Methods in Medical Research 2005;14(4):327-47
83. Whittaker K, Sutton C, Burton C. Pragmatic randomised controlled trials in parenting research: the issue of intention to treat. Journal of Epidemiology & Community Health 2006;60(10):858-64

[84.] Wiens BL, Zhao W. The role of intention to treat in analysis of noninferiority studies. Clinical Trials 2007;4(3):286-91.
[85.] Winnock M. Rancinan C. De Ledinghen V. Couzigou P. Chene G. What hides behind an intention-to-treat analysis?[comment]. [Comment. Letter] Hepatology. 33(4):1014-5, 2001 Apr.
[86.] Wright CC, Sim J. Intention-to-treat approach to data from randomized controlled trials: a sensitivity analysis. Journal of Clinical Epidemiology 2003;56(9):833-42.
[87.] Yusuf S, Garg R, Zucker D. Analyses by the intention-to-treat principle in randomized trials and databases. Pacing & Clinical Electrophysiology 1991;14(12):2078-82.
[88.] http://www.cochrane-net.org/openlearning/HTML/mod14-4.htm Cochrane Collaboration open learning (accessed 9/10)
[89.] http://books.google.ca/books?id=s11gCx4LS_EC&pg=PT510&lpg=PT510&dq=cochrane+intention+to+treat&source=bl&ots=FeOvt0ru- Cochrane 2008 handbook (accessed 01/10)
[90.] Fisher L.D., Dixon, D.O., Herson, J., Frankowski, R.K., Hearron, M.S and Peace, K.E. Intention to treat in clinical trials. In Statistical Issues in Drug Research and Development (American Statistical Associations Group). Peace, K.E. (ed). 1990. pp. 331-350. Marcel Dekker. New York p.341
[91.] Margarete Sandelowski, Whatever Happened to Qualitative Description? Research in Nursing & Health, 2000, 23, 334±340
[92.] Moher D, Hopewell S, Schulz KF, Montori V, Gøtzsche PC, Devereaux PJ, Elbourne D, Egger M, Altman DG; Consolidated Standards of Reporting Trials Group. "CONSORT 2010 Explanation and Elaboration: Updated guidelines for reporting parallel group randomised trials." J Clin Epidemiol. 2010 Aug;63(8):e1-37. Epub 2010 Mar 25.
[93.] Devereaux PJ, Manns BJ, Ghali WA, Quan H, Lacchetti C, Montori VM, et al. Physician interpretations and textbook definitions of blinding terminology in randomized controlled trials. JAMA. 2001;285:2000-3.

APPENDIX A – SEARCH STRATEGY AND ELIGIBILITY CRITERIA

SEARCH STRATEGY

Figure 1 Pilot search strategy

Pilot Search Strategy

MEDLINE from 1950 to May Week 2 2008 search strategy:

1. Intention to treat.mp.
2. Exp Randomized Controlled Trials as Topic/
3. Exp clinical trials as topic/
4. 2 or 3
5. 1 and 4
6. Research design/
7. Exp Data Interpretation, Statistical/
8. 6 or 7
9. 1 and 8
10. 5 or 9

Figure 2 Search strategy used in systematic review

Search Strategy Used in Review

Medline from 1950 to December 2008 search strategy:

1. Intention to treat.mp.
2. Intent to treat.mp.
3. 1 or 2
4. Exp Randomized Controlled Trials as Topic/
5. Exp clinical trials as topic/
6. 4 or 5
7. 3 and 6
8. Research design/
9. Exp Data Interpretation, Statistical/
10. Sensitivity Analysis/
11. 8 or 9 or 10
12. 3 and 11
13. 7 or 12
14. Intention to treat.ti
15. Intent to treat.ti
16. 14 or 15
17. 13 or 16

Figure 3 Title and Abstract Eligibility Criteria

Title and Abstract Eligibility Criteria

1. **Is the article an original RCT or observational study?**
   ***If yes = Exclude, if no = move to question 2**
2. **Is the article a systematic review on a clinical topic with no discussion on methodological topics?**
   ***If yes = Exclude, if no = move to question 3**
3. **Does the article mention 'intention-to-treat' or 'intent-to-treat' in the title or abstract?**
   ***If yes = included, if no = go to question 4**
4. **Is there a good likelihood the article will discuss or define ITT (see below for instructions)?**
   *** If yes = Include, if no = exclude**

Instructions for question 4 - How to determine that there is a good likelihood the study will discuss or define ITT for 3 or more paragraphs:

i. **Does the study discuss terms like 'randomized as analyzed' or similar terminology?**
   ***if yes = include, if no exclude**.

ii. **Does the study discuss other forms of analysis such as:**
   a) "Complete case analysis"
   b) "Worst case scenario"
   c) "Best case scenario"
   d) "Last outcome carried forward"
   e) "Multiple imputation techniques"
   f) "per protocol analysis",
   g) "on treatment analysis"
   h) "analysis by treatment",
   i) "efficacy analysis",
   j) "explanatory analysis"
   k) other similar analysis

*if yes = include, if no= exclude

Figure 4 Full-Text Eligibility Criteria

Full-Text Eligibility Criteria

1. **Is the article an original RCT?**
   ***If yes = Exclude, if no = move to question 2**
2. **Is the article available in English?**
   ***If yes = continue to question 3, if no = exclude**
3. **Does the article comment on ITT AND (see a, b, and c):**
   a) comments on ITT for three or more paragraphs:
      ***If yes = included**
   b) comments on LTFU for two or more paragraphs (so that the total number of paragraphs discussing ITT and LTFU are three or more):
      ***If yes = included**
   c) comments on LTFU for one paragraph and ITT for at least two paragraphs (so that the total number of paragraphs discussing ITT and LTFU are three or more):
      ***If yes = included**

****If no to all (a, b and c) = excluded**
***if yes = include, if no= exclude**

Table 1 A Priori Research Questions

A Priori Research Questions
**1.** What is the definition of ITT in the context of LTFU?
**2.** What is the definition of modified ITT?
**3.** What recommendations for dealing with LTFU.
**4.** What are the biases that arise due to running an analysis other than an ITT? What is the direction of bias?
**5.** What are the circumstances when exclusion of participants conveys no bias?
**6.** What are the recommendations to achieve full follow-up?
**7.** What are the possible differences or the implications or the appropriateness of the use of ITT in preventative versus therapy treatment trials?
**8.** What are the possible differences or the implications or the appropriateness of the use of ITT in effectiveness versus efficacy treatment trials?
**9.** What are the possible differences or the implications or the appropriateness of the use of ITT in superiority versus non-inferiority or equivalence treatment trials?
**10.** What are the Limitations of using an ITT analysis?

APPENDIX B - DATA EXTRACTION FORMS

Below are the first, ninth and final versions for the data extraction forms:

FIGURE 1 VERSION 1 OF THE EXTRACTION FORM B1

FIGURE 2 VERSION 9 OF THE EXTRACTION FORM B6

FIGURE 3 FINAL DATA EXTRACTION FORM B23

FIGURE 1 FIRST VERSION OF THE DATA EXTRACTION FORM

INTENTION TO TREAT (ITT) Study
Data Abstraction Form *(version 1)*

Domain #1: Definition of ITT provided by the paper

The paper defines ITT as one of the following:

1. Comparison of ALL participants as randomized for which you have an outcome, regardless of protocol deviations, participant compliance, withdrawal. For those for which you do not have an outcome who are LTFU you must (check one or more analysis that apply):
    a. Complete case analysis: exclude from the analysis
    b. Worst case scenario: assume all patients in the treatment group had the event and none in the control had it.
    c. Best case scenario: assume all patients in treatment group did NOT have the even and all in the control had it.
    d. Poor outcomes are explicitly allocated in subjects LTFU
    e. Available information on subjects LTFU is used to assign outcome
    f. Last outcome carried forward: Last observed response in subjects LTFU is used to assign the outcome
    g. Multiple imputation techniques
    h. Prescribe an ‘appropriate’ analysis
    i. Other (Please specify): ____

2. Comparison of ALL participants as randomized for which you have an outcome, regardless of protocol deviations, participant compliance, withdrawal. No LTFU is acceptable.

Domain #2: Definition of modified ITT provided by the paper

The paper defines modified-ITT as one of the following:

1. Subjects are analyzed as randomized but some subjects are excluded for the following reasons:
    a. Post-randomization exclusions: Mistakenly randomized patients who failed to fulfill the study inclusion criteria
    b. Patients randomized but never received the allocated therapy.
    c. Patients lost to follow-up (complete case analysis)
    d. Patients who withdrew the study (the treatment) but are not lost to follow -up

Domain #3: Recommendation for dealing with LFUP

The paper recommends that one deals with LTFU assuming the following:

a. Complete case analysis: exclude from the analysis
b. Worst case scenario: assume all patients in the treatment group had the event and none in the control had it.
c. Best case scenario: assume all patients in treatment group did NOT have the even and all in the control had it.
d. Poor outcomes are explicitly allocated in subjects LTFU
e. Available information on subjects LTFU is used to assign outcome
f. Last outcome carried forward: Last observed response in subjects LTFU is used to assign the outcome
g. Multiple imputation techniques
h. Prescribe an ‘appropriate’ analysis
i. Other (Please specify): ____

Domain #4: Other types of analysis

a. Full compliance analysis
b. Per-protocol analysis ("on treatment analysis"): refers to an analysis which uses results from only those participants who completed the trial and who complied with their allocated intervention.
c. Treatment received (TR) analysis: when an analysis is run based on the intervention the participants received without considering their randomization
d. As treated analysis: counts everyone who received a treatment irrespective of the intervention originally assigned
e. Compliers only analysis: count only those individuals assigned to a treatment who actually received the treatment.
f. Available case analysis is one in which the data is analyzed for all the participants whose outcome is observed.

Domain #5: Biases if not ITT (For the main analysis?)

The bias that arises due running analysis other than ITT:
a. Overestimates the benefit (risk of False positive)
b. Underestimate the benefit (risk of False negative)
c. Nonconservative estimate of treatment effect

Domain #6: When exclusion does not convey bias

Decision to excluding patient conveys no bias if:

a. The decision is made by researchers (or independent adjudicator committee) blinded to treatment allocation and outcome.
b. Decision of excluding should be made on the basis of information not related to either the allocated treatment neither the outcomes.
c. Ineligible patients are mistakenly randomized into a trial.
d. Patients who never received the intervention.
e. Decisions are made after evaluating all randomized patients.
f. Decisions are based solely on pre-randomization conditions.

Domain #7: Recommendation to achieve ITT ideal (design and conduct of RCT)

1. Avoid eligibility errors
2. Minimize dropouts from treatment,
3. Minimize crossover of participants between groups
4. Minimize LTFU by:
    a. using an active run- in phase,
    b. thorough consent process,
    c. education of investigators
    d. ongoing clinical support during the trial.
    e. having an investigator dedicated to checking follow-up
    f. using simple outcomes (e.g. death)
    g. getting detailed contact information of participant and relatives
    h. increase the planned sample size if a dilution effect is expected to occur.
    i. minimize the post-randomization exclusions.

FIGURE 2 VERSION 9 OF THE EXTRACTION FORM

INTENTION TO TREAT (ITT) Study
Data Abstraction Form *(version 9)*

ID information

**Reviewer initials**	
**Study ID**	
**Author (Lastname, Initials)**	
**Year**	
**Journal**	

Domain #1: Definition of ITT provided by the paper.

**The paper provides one or more of the following 4 definitions for ITT (yes=1, no=0)**<br><br>***If no, go to Domain #2. If yes, select all the that apply from the following definitions.**	
**Definition 1**:<br>Comparison of ALL participants as randomized for which investigators have recorded the outcome of interest, regardless of protocol deviations and participant compliance: **For participants for whom outcome data is unavailable (i.e. LTFU) investigators <u>MUST</u> do the following (check all that apply):**	**Select one option (i-v)**<br><br>*If option v "Definition not mentioned by author" is selected go to definition 2 in domain #1. Otherwise answer a-i.
a. **Complete case analysis**: exclude from the analysis.	**Select one option (i-v)**
b. **Worst case scenario**: assume all participants in the treatment group had the event and none in the control had it.	**Select one option (i-v)**
c. **Best case scenario**: assume all participants in treatment group did NOT have the event and all in the control had it.	**Select one option (i-v)**
d. **All had outcome**: assume all those LTFU had	**Select one option (i-v)**

suffered the outcome of interest.	
e. **All had no outcome**: assume none of those LTFU had suffered the outcome of interest.	**Select one option (i-v)**
f. **Use of available data to impute missing outcome**: available information on participants LTFU is used to assign outcome.	**Select one option (i-v)**
g. **Last outcome carried forward**: last observed response in participants LTFU is used to assign the outcome.	**Select one option (i-v)**
h. **Multiple imputation techniques**	**Select one option (i-v)**
i. Other (please specify):	**Select one option (i-v)**

2. **Definition 2:** Comparison of ALL participants as randomized for which investigators have recorded the outcome of interest, regardless of protocol deviations and participant compliance. **For participants for whom outcome data is unavailable (i.e. LTFU) investigators <u>MAY</u> do the following (check all that apply):**	**Select one option (i-v)**<br><br>*If option v "Definition not mentioned by author" is selected go to definition 3 in domain #1. Otherwise answer a-i.
a. **Complete case analysis**: exclude from the analysis.	**Select one option (i-v):**
b. **Worst case scenario**: assume all participants in the treatment group had the event and none in the control had it.	**Select one option (i-v):**
c. **Best case scenario**: assume all participants in treatment group did NOT have the event and all in the control had it.	**Select one option (i-v):**
d. **All had outcome**: assume all those LTFU had suffered the outcome of interest.	**Select one option (i-v):**
e. **All had no outcome**: assume none of those LTFU had suffered the outcome of interest.	**Select one option (i-v):**
f. **Use of available data to impute missing outcome**: available information on participants LTFU is used to assign outcome.	**Select one option (i-v):**
g. **Last outcome carried forward**: last observed response in participants LTFU is used to assign the outcome.	**Select one option (i-v):**
h. **Multiple imputation techniques**	**Select one option (i-v):**

i. Other (please specify):	**Select one option (i-v):**

3.**Definition 3:**<br>Comparison of ALL participants as randomized for which investigators have recorded the outcome of interest, regardless of protocol deviations and participant compliance: **ITT cannot be done if LTFU occurred (i.e. FULL FOLLOW-UP REQUIRED).**	**Select one option (i-v):**
4**. Definition 4:**<br>Comparison of ALL participants as randomized for which investigators have recorded the outcome of interest, regardless of protocol deviations and participant compliance: **ITT is a SEPARATE ISSUE from LTFU.**	**Select one option (i-v):**
**5. Definition 5:**<br>Comparison of ALL participants as randomized for which investigators have recorded the outcome of interest, regardless of protocol deviations and participant compliance: **It is UNCLEAR whether the authors believe that you must have no LTFU to be equivalent to ITT (i.e., Definition #3) or whether they believe in imputation or excluding patients (i.e., there is unclear direction).**	

Domain 1: Main Point(s)

i. If applicable please copy/paste the paragraph(s) or statement(s) made by the author that you believed to be important, unique or useful regarding this domain:

ii. Please provide a brief summary that captures the author's main point(s) that you believe to be most important, unique or useful regarding this domain (if this information is already extracted leave this question blank and please move to the next domain):

*** The rest of the data abstraction is to be filled using the author's preferred definition.**

Domain #2: Definition of modified-ITT provided by the paper

Does the paper define **modified-ITT**? **(yes=1, no=0)**
***If no, go to Domain #3.**

If yes, answer the following question(s): The paper defined modified-ITT as one of the following (check all that apply 1 and/or 2):

Y/N: 1. Participants are analyzed as randomized but some participants are excluded (check all that apply i-iv):
Y/N: i) **Post-randomization exclusion**: participants randomized but **never received** the allocated therapy (if there are any additional details please specify and/or check all that apply):

Y/N: a. Appropriate if patients were blinded to allocation.
Y/N: b. Decision of excluding should be made on the basis of information NOT related to either the allocated intervention or outcomes.
Y/N: c. None specified.
Y/N:d. **Other** criteria related to participants who never received the allocated therapy (please specify):

Y/N: ii) **Post-randomization exclusion**: participants who **withdrew** their consent but are not LTFU (if there are any additional details please specify and/or check all that apply):

Y/N: a. Decision of excluding should be made on the basis of information NOT related to either the allocated intervention or outcomes.
Y/N: b. None specified.
Y/N: c. **Other** criteria related to participants who **withdrew** their consent but are not LTFU (please specify):

Y/N: iii) **Post-randomization exclusion**: Ineligible participants who are **mistakenly randomized** (if there are any additional details please specify and/or check all that apply):

Y/N: a. Individual that makes decision to exclude (i.e. adjudicator) is blinded to treatment.
Y/N: b. None specified.
Y/N: c. Other criteria related to ineligible participants who are **mistakenly randomized** (please specify):

Y/N: iv) **Post-randomization exclusion**: participants excluded because of **center exclusion** (if there are any additional details please specify and/or check all that apply):

Y/N: a. None specified.

Y/N: b. Other criteria related to participants excluded because of **center exclusion** (please specify):

Y/N: v) Participants LTFU.

Y/N: vi) Other than **never received, withdrew, mistakenly randomized or center exclusion**. (please specify):

Y/N: 2. Other definition of modified-ITT that doesn't involve exclusion of participants (e.g., analytic strategies). Please specify:

Domain 2: Main Point(s)

i. If applicable please copy/paste the paragraph(s) or statement(s) made by the author that you believed to be important, unique or useful regarding this domain:

ii. Please provide a brief summary that captures the author's main point(s) that you believe to be most important, unique or useful regarding this domain (if this information is already extracted leave this question blank and please move to the next domain):

Domain #3: Recommendation for dealing with LTFU provided by the paper

Does the paper make a recommendation for how to deal with LTFU?

(yes=1, no=0)

***If no, go to Domain #4.**

If yes, answer the following question(s): The paper recommends that one deals with LTFU assuming the following (check all that apply (a-k):

Y/N: a. **Complete case analysis**: exclude from the analysis.
Y/N: b. **Worst case scenario**: assume all participants in the treatment group had the event and none in the control had it.
Y/N: c. **Best case scenario**: assume all participants in treatment group did NOT have the event and all in the control had it.
Y/N: d. **All had outcome**: assume all those LTFU had suffered the outcome of interest.
Y/N: e. **All had no outcome**: assume none of those LTFU had suffered the outcome of interest.
Y/N: f. **Use of available data**: available information on participants LTFU is used to assign outcome.
Y/N: g. **Last outcome carried forward**: last observed response in participants LTFU is used to assign the outcome.
Y/N: h. **Multiple imputation techniques.**
Y/N: i. Minimize LTFU.
Y/N: j. Other (please specify):
Y/N: k. Unclear.

Domain 3: Main Point(s)

i. If applicable please copy/paste the paragraph(s) or statement(s) made by the author that you believed to be important, unique or useful regarding this domain:

ii. Please provide a brief summary that captures the author's main point(s) that you believe to be most important, unique or useful regarding this domain (if this information is already extracted leave this question blank and please move to the next domain):

Domain #4: Biases that arise if not ITT (according to the authors preferred definition of ITT)

Does the paper suggest biases that will result due to running analysis other than ITT? **(yes=1, no=0)**

***If no, go to Domain #5.**

If yes, answer the following question(s): The paper suggests the following biases will result due to running analysis other than ITT (check all that apply and answer yes or no to questions 1-6):

**1. Overestimate the effect:**	**Definitely yes**	**Probably yes**
a) Overestimates the benefit (risk of false positive).	☐	☐
b) Overestimate the harm.	☐	☐
c) Not specified whether harm.	☐	☐
d) Not specified whether benefit.	☐	☐
**2. Underestimate the effect:**	Definitely yes	Probably yes
a) Underestimate the benefit (risk of false negative).	☐	☐
b) Underestimate the harm.	☐	☐
c) Not specified whether harm.	☐	☐
d) Not specified whether benefit.	☐	☐

Y/N: 3. Bias, explicit statement that direction of bias can not be predicted.

Y/N: 4. Bias, no explicit direction specified.

Y/N: 5. Other terminology used (please specify, e.g. author does not use “bias” but uses “not sound” or “not valid”):

Domain 4: Main Point(s)

i. If applicable please copy/paste the paragraph(s) or statement(s) made by the author that you believed to be important, unique or useful regarding this domain:

ii. Please provide a brief summary that captures the author's main point(s) that you believe to be most important, unique or useful regarding this domain (if this information is already extracted leave this question blank and please move to the next domain):

Domain #5: When exclusion does not convey bias or when groups are omitted and are still consistent with ITT.

Does the paper comment on when exclusion does not convey bias?
(yes=1, no=0)
***If no, go to Domain #6.**

If yes, answer the following question(s): Decision to exclude patient conveys no bias if participants are analyzed as randomized but some participants are excluded (check all that apply i-iv):
Y/N: i) **Post-randomization exclusion**: participants randomized but **never received** the allocated therapy (if there are any additional details please specify and/or check all that apply):
Y/N: a. Appropriate if patients were blinded to allocation.
Y/N: b. Decision of excluding should be made on the basis of information NOT related to either the allocated intervention or outcomes.
Y/N: c. None specified.
Y/N: d. **Other** criteria related to participants who never received the allocated therapy (please specify):

Y/N: ii) **Post-randomization exclusion**: Ineligible participants who are **mistakenly randomized** (if there are any additional details please specify and/or check all that apply):
Y/N: a. If the individual that makes decision to exclude (i.e. adjudicator) is blinded to treatment.
Y/N: b. None specified.
Y/N: c. Other criteria related to ineligible participants who are **mistakenly randomized** (please specify):

Y/N: iii) **Post-randomization exclusion**: participants excluded because of study **center exclusion** (if there are any additional details please specify and/or check all that apply):
Y/N: a. None specified.
Y/N: b. Other criteria related to participants excluded because of **center exclusion** (please specify):

Y/N: iv) Other than **never received, withdrew, mistakenly randomized or center exclusion**. (please specify):

Domain 5: Main Point(s)

i. If applicable please copy/paste the paragraph(s) or statement(s) made by the author that you believed to be important, unique or useful regarding this domain:

ii. Please provide a brief summary that captures the author's main point(s) that you believe to be most important, unique or useful regarding this domain (if this information is already extracted leave this question blank and please move to the next domain):

Domain #6: Recommendation to achieve full follow-up [categories in progress]

Does the paper make recommendations to achieve full follow-up?

(yes=1, no=0)

***If no, go to Domain #7.**

If yes, answer the following question(s): the paper recommends the following strategies to achieve full follow-up (check all that apply):

Y/N: 1) Avoid eligibility errors.
Y/N: 2) Minimize 'dropouts' from treatment.
Y/N: 3) Minimize crossover of participants between groups.
Y/N: 4) Minimize the post-randomization exclusions.
Y/N: 5) Excluding participants that may be hard to follow-up (eg. No fixed addresses or are intellectually handicapped)
Y/N: 6) Having a 'Tracker' dedicated to finding the outcome of participants that lost contact.
Y/N: 7) Thorough consent process (eg. Be clear about tribulations of participating and obtain consent to track participant).
Y/N: 8) Request detailed contact information of participant and/or relatives
Y/N: 9) Using an active run- in phase.
Y/N: 10) Education of investigators.
Y/N: 11) Ongoing clinical support during the trial.
Y/N: 12) Using simple outcomes (e.g. death).
Y/N: 13) Getting detailed contact information of participant and relatives.
Y/N: 14) Other (please specify):

☐**Domain #7:** Did the authors discuss the possible differences or the implications or the appropriateness of the use of ITT in preventative versus therapy treatment trials?
Please Select Yes or No

***If no, go to Domain #8.**

If yes, answer the following question(s):
Main Point(s)

i. If applicable please copy/paste the paragraph(s) or statement(s) made by the author that you believed to be important, unique or useful regarding this domain:

ii. Please provide a brief summary that captures the author's main point(s) that you believe to be most important, unique or useful regarding this domain:

☐**Domain #8:** Did the authors discuss the possible differences or the implications or the appropriateness of the use of ITT in management (practical, pragmatic, effectiveness) versus explanatory (mechanistic, efficacy) treatment trials?

(yes= 1, no=0)

***If no, go to Domain #9.**

If yes, answer the following question(s):

Main Point(s)

i. If applicable please copy/paste the paragraph(s) or statement(s) made by the author that you believed to be important, unique or useful regarding this domain:

ii. Please provide a brief summary that captures the author's main point(s) that you believe to be most important, unique or useful regarding this domain:

☐**Domain #9:** Did the authors discuss the possible differences or the implications or the appropriateness of the use of ITT in superiority versus non-inferiority or equivalence treatment trials?
(yes=1, no=0)

***If no, go to Domain #10.**

**** If these comments were** already provided in domain #4, question 5, **go to Domain #10.**

If yes and these are additional comments that were not provided in domain #4, answer the following question(s):

Main Point(s)

i. If applicable please copy/paste the paragraph(s) or statement(s) made by the author that you believed to be important, unique or useful regarding this domain:

ii. Please provide a brief summary that captures the author's main point(s) that you believe to be most important, unique or useful regarding this domain:

☐**Domain #10: Limitations of ITT (using the papers preferred definition, if applicable)** **[new domain: criteria in progress]**

Does the paper provide limitations of the ITT principle?
(yes=1, no=0)
***If no, go to Domain #11.**

Y/N: 1. Bias depends on whether it is superiority vs. inferiority. If yes,
Check all that apply:

	Definitely yes	Probably yes
i. In the case of ITT, **Superiority bias toward underestimation of effect.**	☐	☐
ii. **Noninferiority bias toward showing noninferiority.**	☐	☐
**iii. Y/N:** c) Other (please specify):		

Main Point(s)

i. If applicable please copy/paste the paragraph(s) or statement(s) made by the author that you believed to be important, unique or useful regarding this domain:

ii. Please provide a brief summary that captures the author's main point(s) that you believe to be most important, unique or useful regarding this domain:

☐ **Domain #11: Withdrawal of consent [new domain: criteria in progress]**

(ii) **Post-randomization exclusion**: participants who **withdrew** consent but are not LTFU (if there are any additional details please specify and/or check all that apply):

a. Decision of excluding should be made on the basis of information NOT related to either the allocated intervention or outcomes.

b. None specified.

c. **Other** criteria related to participants who **withdrew** from the study but are not LTFU (please specify):

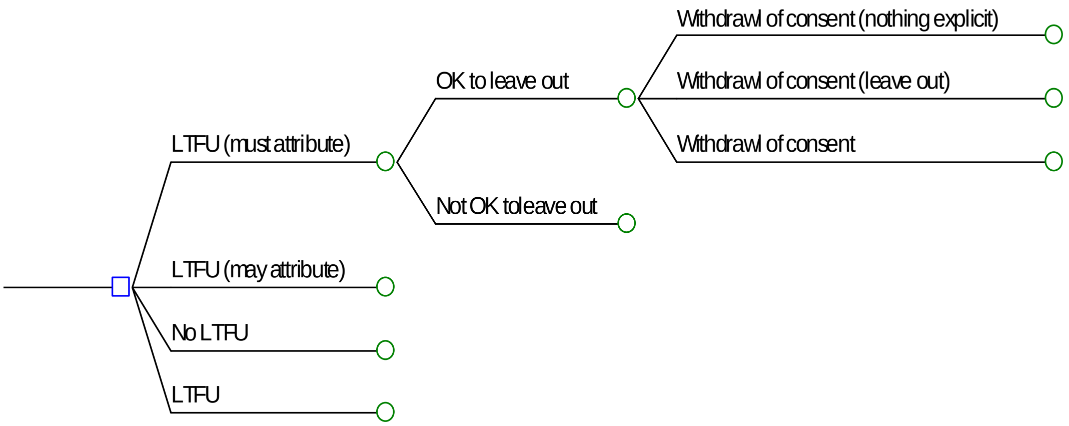

☐ Do the authors an ethical imperative to exclude patients that withdrew consent but despite data being available [i.e. not LTFU]?
Please Select Yes or No

1. **If yes, please specify:**

DOMAIN 11

It should be included, a comment on how to deal with withdrawal, i.e., do the authors explicitly state a method? If yes, do they mention one of the following:

- Ethically, do not use the data since consent was withdrawn
- One may use data even though consent was withdrawn

FIGURE 3 FINAL DATA EXTRACTION FORM

INTENTION TO TREAT (ITT) Study

Data Abstraction Form *(version 16.6)*

**Reviewer initials (e.g., M.A.)**	
**3-digit Study ID (e.g., 052)**	
**Author (Last name, first name)**	
**Year (e.g., 2003)**	
**Journal (e.g. JAMA)**	

Domain #1: Definition of ITT provided by the paper.

<table>
<tr><td colspan="2">1) The paper gives a preferred or possible definition of ITT (please select ‘Yes’ or ‘No’): Y/N?
* If ‘No’, go to next domain (i.e., you are FINISHED with this domain).
** If ‘Yes’, answer question 2a.</td></tr>
<tr><td>2a) The paper comments on the definition of ITT AND includes some comment about loss to follow-up (LTFU) in the definition of ITT: Y/N?

* If ‘No’ to ‘2a’, answer question 2b and then you are FINISHED with domain #1.
** If ‘Yes’ to ‘2a’, <u>select ALL that apply from definitions 1 to 4 in green (below):</u></td><td>2b) The paper defines ITT as the comparison of: Select one option (i-ii)

* If you answered ‘No’ to question ‘2a’, after you answer ‘2b’, you are FINISHED with domain #1. Please go to domain #2 “Definition of Modified ITT Provided by Paper” on pg 5.</td></tr>
<tr><td colspan="2">Important notes before filling out Definitions 1 to 4:
i. If you answered ‘yes’ to question #1 AND ‘yes’ to question #2a you MUST choose an answer for each definition below (ie. Definition 1 to 4).
ii. An author can ‘endorse’ more than one definition for ITT (e.g. Can endorse definition 1 and definition 2).
iii. You MUST answer the ‘CLARITY Clarification’ section at the bottom of Domain #1.</td></tr>
</table>

**Definition 1**: Comparison of ALL participants as randomized for which investigators have recorded the outcome of interest, regardless of protocol deviations and participant compliance: **For participants for whom outcome data is unavailable (i.e. LTFU) investigators MUST do the following (check all that apply):**	**Select one option (i-v)**<br>**VERY IMPORTANT NOTE for definition 1:**<br>***If you chose any of options i-iv for definition 1 you MUST answer all of 1a-1j below.**<br>***If you chose option v "Definition not mentioned by author" go to Definition 2 in domain #1.**
1a. **Complete case analysis**: exclude from the analysis.	**Select one option (i-vi)**<br>*if this is a mandatory option for ITT than fill out 'definition specifically excluded' for definition #3 below.

1b. **Worst case scenario**: assume all participants in the treatment group had the event and none in the control had it.	**Select one option (i-vi)**
1c. **Best case scenario**: assume all participants in treatment group did NOT have the event and all in the control had it.	**Select one option (i-vi)**
1d. **All had outcome**: assume all those LTFU had suffered the outcome of interest.	**Select one option (i-vi)**
1e. **All had no outcome**: assume none of those LTFU had suffered the outcome of interest.	**Select one option (i-vi)**
1f. **Use of available data to impute missing outcome**: available information on participants LTFU is used to assign outcome.	**Select one option (i-vi)**
1g. **Last outcome carried forward**: last observed response in participants LTFU is used to assign the outcome.	**Select one option (i-vi)**
1h. **Multiple imputation techniques**	**Select one option (i-vi)**
1i. **Sensitivity analysis**: testing more than one assumption and look at implications of alternative assumptions.	**Select one option (i-vi)**

1j. **Other** (please specify):	**Select one option (i-vi)**

**Definition 2:** Comparison of ALL participants as randomized for which investigators have recorded the outcome of interest, regardless of protocol deviations and participant compliance. **For participants for whom outcome data is unavailable (i.e. LTFU) investigators <u>MAY</u> do the following (check all that apply):**	**Select one option (i-v)** *If option v "Definition not mentioned by author" is selected go to definition 3 in domain #1. Otherwise answer a-j.
2a. **Complete case analysis**: exclude from the analysis.	**Select one option (i-vi)**
2b. **Worst case scenario**: assume all participants in the treatment group had the event and none in the control had it.	**Select one option (i-vi)**
2c. **Best case scenario**: assume all participants in treatment group did NOT have the event and all in the control had it.	**Select one option (i-vi)**
2d. **All had outcome**: assume all those LTFU had suffered the outcome of interest.	**Select one option (i-vi)**
2e. **All had no outcome**: assume none of those LTFU had suffered the outcome of interest.	**Select one option (i-vi)**
2f. **Use of available data to impute missing outcome**: available information on participants LTFU is used to assign outcome.	**Select one option (i-vi)**
2g. **Last outcome carried forward**: last observed response in participants LTFU is used to assign the outcome.	**Select one option (i-vi)**
2h. **Multiple imputation techniques**	**Select one option (i-vi)**
2i. **Sensitivity analysis**: testing more than one assumption and look at implications of alternative assumptions.	**Select one option (i-vi)**
2j. Other (please specify):	**Select one option (i-vi)**

**Definition 3:**<br>Comparison of ALL participants as randomized for which investigators have recorded the outcome of interest, regardless of protocol deviations and participant compliance: **ITT cannot be done if LTFU occurred (i.e. <u>FULL FOLLOW-UP IS REQUIRED</u>).**	**Select one option (i-v)**
**Definition 4:**<br>Comparison of ALL participants as randomized for which investigators have recorded the outcome of interest, regardless of protocol deviations and participant compliance: **ITT is a <u>SEPARATE ISSUE</u> from LTFU.**	**Select one option (i-v)**

**3) CLARITY explanation:**<br>Comparison of ALL participants as randomized for which investigators have recorded the outcome of interest, regardless of protocol deviations and participant compliance: **It is <u>UNCLEAR</u> whether the authors preferred to impute or exclude patients (i.e., Definition 1 or 2) for ITT, whether they prefer 'FULL FOLLOW-UP IS REQUIRED' to be equivalent to ITT (i.e., Definition 3), or whether they prefer ITT is a 'SEPARATE ISSUE from LTFU' (Definition 4).**	**Select one option (i-iv)**

**Domain #1: MAIN POINT(s)**
**i. If applicable please copy/paste the paragraph(s) or statement(s) made by the author that you believed to be important, unique or useful regarding this domain:**
**Page/paragraph number (e.g., pg. 10/para. 2):**
**ii. Please provide a brief summary that captures the author's main point(s) that you believe to be most important, unique or useful regarding this domain (if this information is already extracted leave this question blank and please move to the next domain):**
**Page/paragraph number (e.g., pg. 10/para. 2):**

*** The rest of the data abstraction is to be filled using the author's preferred definition in Domain #1.**

Domain #2: Definition of modified-ITT provided by the paper

**1.** Does the paper define **modified-ITT**?<br>***If 'No', you are FINISHED with this domain.**<br>****If 'Yes', answer ALL of the following question(s):**<br>The paper **defined modified-ITT** as one of the following (check all that apply, Definition 1 and/or Definition 2):	**Y/N?**
**Definition 1**: Participants are analyzed as randomized BUT some participants are EXCLUDED (check all that apply i-iv):	**Y/N?**
i) **Post-randomization exclusion**: participants randomized but **never received** the allocated therapy:	**Y/N?**
a. Appropriate if patients were blinded to allocation NOT related to either the allocated intervention or outcomes.	**Y/N?**
b. Decision of excluding should be made on the basis of information NOT related to either the allocated intervention or outcomes.	**Y/N?**
c. None specified.	**Y/N?**
d. **Other** criteria related to participants who never received the allocated therapy (please specify):	**Y/N?**
ii) **Post-randomization exclusion**: participants who **withdrew** their consent but are not LTFU:	**Y/N?**
a. Decision of excluding should be made on the basis of information NOT related to either the allocated intervention or outcomes.	**Y/N?**
b. None specified.	**Y/N?**
c. **Other** criteria related to participants who **withdrew** their consent but are not LTFU (please specify):	**Y/N?**
iii) **Post-randomization exclusion**: Ineligible participants who are **mistakenly randomized:**	**Y/N?**
a. Individual that makes decision to exclude (i.e. adjudicator) is blinded to treatment.	**Y/N?**
b. None specified.	**Y/N?**
c. Other criteria related to ineligible participants who are **mistakenly randomized** (please specify):	**Y/N?**
iv) **Post-randomization exclusion**: participants excluded because of **center exclusion**:	**Y/N?**
a. None specified.	**Y/N?**
b. Other criteria related to participants excluded because of **center exclusion. P**lease specify:	**Y/N?**
**v) Participants LTFU.**	**Y/N?**
vi) Other than **never received, withdrew, mistakenly randomized or center exclusion**. Please specify:	**Y/N?**
**Definition 2**: Other definition of modified-ITT that DOESN'T involve	**Y/N?**

exclusion of participants (e.g., analytic strategies). Please specify:

**Domain #2: MAIN POINT(s)**
**i. If applicable please copy/paste the paragraph(s) or statement(s) made by the author that you believed to be important, unique or useful regarding this domain:**
**Page/paragraph number (e.g., pg. 10/para. 2):**
**ii. Please provide a brief summary that captures the author's main point(s) that you believe to be most important, unique or useful regarding this domain (if this information is already extracted leave this question blank and please move to the next domain):**
**Page/paragraph number (e.g., pg. 10/para. 2):**

Domain #3: Recommendation for dealing with LTFU provided by the paper outside of the context of ITT

Does the paper make a recommendation for how to deal with LTFU outside of the context of ITT?<br><br>***If 'No', you are FINISHED with this domain.**<br>****If 'Yes', answer ALL of the following question(s):**	**Y/N?**
The paper recommends that one deals with LTFU assuming the following [check all that apply (a-k)]:	
a. **Complete case analysis**: exclude from the analysis.	**Y/N?**
b. **Worst case scenario**: assume all participants in the treatment group had the event and none in the control had it.	**Y/N?**
c. **Best case scenario**: assume all participants in treatment group did NOT have the event and all in the control had it.	**Y/N?**
d. **All had outcome**: assume all those LTFU had suffered the outcome of interest.	**Y/N?**
e. **All had no outcome**: assume none of those LTFU had suffered the outcome of interest.	**Y/N?**
f. **Use of available data**: available information on participants LTFU is used to assign outcome.	**Y/N?**
g. **Last outcome carried forward**: last observed response in participants LTFU is used to assign the outcome.	**Y/N?**
h. **Multiple imputation techniques.**	**Y/N?**
i. **Sensitivity analysis**: testing more than one assumption and look at implications of alternative assumptions.	**Y/N?**
**Were specific sensitivity analyses recommended?**<br><br>**If yes, list them:**	**Y/N?**
j. **Minimize LTFU.**	**Y/N?**
k. **Other** (please specify):	**Y/N?**
l. **Unclear**.	**Y/N?**

**Domain #3: MAIN POINT(s)**
**i. If applicable please copy/paste the paragraph(s) or statement(s) made by the author that you believed to be important, unique or useful regarding this domain:**
**Page/paragraph number (e.g., pg. 10/para. 2):**
**ii. Please provide a brief summary that captures the author's main point(s) that you believe to be most important, unique or useful regarding this domain (if this information is already extracted leave this question blank and please move to the next domain):**
**Page/paragraph number (e.g., pg. 10/para. 2):**

Domain #4: Limitations of ITT (using the papers preferred definition of ITT, if applicable)

Does the paper provide limitations of the ITT principle?<br><br>***If 'No', you are FINISHED with this domain.**<br>****If 'Yes', answer ALL of the following question(s):**		**Y/N?**
1. The paper suggests that the bias depends on whether it is superiority vs. inferiority/equivalence.<br><br>If yes, check all that apply:		**Y/N?**
	**Definitely yes**	**Probably yes**
i. In the case of ITT, superiority bias toward underestimation of effect.	☐	☐
ii. In the case of ITT, noninferiority/equivalence bias toward showing noninferiority/equivalence.	☐	☐
iii. Other, please specify (e.g. selection bias):		

**Domain #4: MAIN POINT(s)**
**i. If applicable please copy/paste the paragraph(s) or statement(s) made by the author that you believed to be important, unique or useful regarding this domain:**
**Page/paragraph number (e.g., pg. 10/para. 2):**
**ii. Please provide a brief summary that captures the author's main point(s) that you believe to be most important, unique or useful regarding this domain (if this information is already extracted leave this question blank and please move to the next domain):**

**Page/paragraph number (e.g., pg. 10/para. 2):**

Domain #5: When exclusion does not convey bias or when groups are omitted and are still consistent with ITT.

**1. Author addresses exclusion without bias.**<br>***If 'No', you are FINISHED with this domain.**<br>****If 'Yes', answer ALL of the following question(s):**	**Y/N?**
**2. Author mentions exclusion without bias and… choose i-iii … consistent with ITT.**	
**3. Decision to exclude patient conveys no bias if participants are analyzed as randomized but some participants are excluded**<br>***If 'No', you are FINISHED with this domain.**<br>****If 'Yes', check all that apply i-iv:**	**Y/N?**
i) **Post-randomization exclusion**: participants randomized but **never received** the allocated therapy (if there are any additional details please specify and/or check all that apply):	**Y/N?**
a. Appropriate if patients were blinded to allocation.	**Y/N?**
b. Decision of excluding should be made on the basis of information NOT related to either the allocated intervention or outcomes.	**Y/N?**
c. None specified.	**Y/N?**
d. **Other** criteria related to participants who never received the allocated therapy. Please specify:	**Y/N?**
ii) **Post-randomization exclusion**: Ineligible participants who are **mistakenly randomized** (if there are any additional details please specify and/or check all that apply):	**Y/N?**
a. If the individual that makes decision to exclude (i.e. adjudicator) is blinded to treatment.	**Y/N?**
b. None specified.	**Y/N?**
c. Other criteria related to ineligible participants who are **mistakenly randomized**. Please specify:	**Y/N?**
iii) **Post-randomization exclusion**: participants excluded because of study **center exclusion** (if there are any additional details please specify and/or check all that apply):	**Y/N?**
a. None specified.	**Y/N?**
b. Other criteria related to participants excluded because of **center exclusion** Please specify:	**Y/N?**

iv) Other than **never received, withdrew, mistakenly randomized or center exclusion**. Please specify:	**Y/N?**
4. Author states that ANY post-randomization exclusions may introduce bias.	**Y/N?**

**Domain #5: MAIN POINT(s)**
**i. If applicable please copy/paste the paragraph(s) or statement(s) made by the author that you believed to be important, unique or useful regarding this domain:**
**Page/paragraph number (e.g., pg. 10/para. 2):**
**ii. Please provide a brief summary that captures the author's main point(s) that you believe to be most important, unique or useful regarding this domain (if this information is already extracted leave this question blank and please move to the next domain):**
**Page/paragraph number (e.g., pg. 10/para. 2):**

Domain #6: Biases that arise if NOT ITT (using the papers preferred definition of ITT, if applicable)

Does the paper suggest biases that will result due to running analysis other than ITT? **Y/N?**

***If 'No', you are FINISHED with this domain.**
****If 'Yes', answer ALL of the following question(s):**

Y/N? 1. The paper suggests the following biases will result due to running analysis other than ITT (check all that apply and answer yes or no to questions 1-6):

**i. Overestimate the effect:**	**Definitely yes**	**Probably yes**
a) Overestimates the benefit (false positive).	☐	☐
b) Overestimate the harm.	☐	☐
c) Not specified whether harm or benefit.	☐	☐
**ii. Underestimate the effect:**	**Definitely yes**	**Probably yes**
a) Underestimate the benefit (false negative).	☐	☐
b) Underestimate the harm.	☐	☐
c) Not specified whether harm or benefit	☐	☐

Y/N? 2. Bias, explicit statement that direction of bias can not be predicted.

Y/N? 3. Bias, no explicit direction specified.

**Domain #6: MAIN POINT(s)**
**i. If applicable please copy/paste the paragraph(s) or statement(s) made by the author that you believed to be important, unique or useful regarding this domain:**
**Page/paragraph number (e.g., pg. 10/para. 2):**
**ii. Please provide a brief summary that captures the author's main point(s) that you believe to be most important, unique or useful regarding this domain (if this information is already extracted leave this question blank and please move to the next domain):**

**Page/paragraph number (e.g., pg. 10/para. 2):**

Domain #7: Withdrawal of consent to participate in trial.

**Do the authors discuss ethical issues of withdrawl of consent (i.e. the ethical imperative to exclude such patients despite data being available)?**<br><br>***If 'No', you are FINISHED with this domain.**<br>****If 'Yes', answer ALL of the following question(s):**	**Y/N?**
**Do the authors mention any of the following:**	**Y/N?**
a. There is an ethical imperative to exclude patients that withdraw consent despite data being available (i.e. one may not use data because consent was withdrawn).<br><br>If additional information is given please specify:	**Y/N?**
b. There is NO ethical imperative to exclude patients that withdraw consent despite data being available (i.e. one may use data even though consent was withdrawn).<br><br>If additional information is given, please specify:	**Y/N?**

**Domain #7: MAIN POINT(s)**
**i. If applicable please copy/paste the paragraph(s) or statement(s) made by the author that you believed to be important, unique or useful regarding this domain:**
**Page/paragraph number (e.g., pg. 10/para. 2):**
**ii. Please provide a brief summary that captures the author's main point(s) that you believe to be most important, unique or useful regarding this domain (if this information is already extracted leave this question blank and please move to the next domain):**

**Page/paragraph number (e.g., pg. 10/para. 2):**

Domain #8: Recommendation to achieve full follow-up regardless of definition of ITT

Does the paper make recommendations to achieve full follow-up?<br>***If 'No', you are FINISHED with this domain.**<br>****If 'Yes', answer ALL of the following question(s):**	**Y/N?**
The paper recommends the following strategies to achieve full follow-up (check all that apply):	**Y/N?**
1) Avoid eligibility errors.	**Y/N?**
**2**) Minimize 'dropouts' from treatment.	**Y/N?**
3) Minimize crossover of participants between groups.	**Y/N?**
4) Minimize the post-randomization exclusions.	**Y/N?**
5) Excluding participants that may be hard to follow-up:	**Y/N?**
a) No fixed addresses	**Y/N?**
b) are intellectually handicapped	**Y/N?**
b) Other (please specify):	**Y/N?**
6) Having a 'Tracker' dedicated to finding the outcome of participants that lost contact.	**Y/N?**
7) Thorough consent process (eg. Be clear about tribulations of participating and obtain consent to track participant).	**Y/N?**
8) Request detailed contact information of participant and/or relatives	**Y/N?**
9) Education of investigators.	**Y/N?**
10) Ongoing clinical support during the trial.	**Y/N?**
11) Using simple outcomes (e.g. death).	**Y/N?**
12) Continue to follow-up even after 'dropout' from treatment.	**Y/N?**
13) Using an active run- in phase.	**Y/N?**
14) Other (please specify):	**Y/N?**

**Domain #8: MAIN POINT(s)**
**i. If applicable please copy/paste the paragraph(s) or statement(s) made by the author that you believed to be important, unique or useful regarding this domain:**
**Page/paragraph number (e.g., pg. 10/para. 2):**
**ii. Please provide a brief summary that captures the author's main point(s) that you believe to be most important, unique or useful regarding this domain (if this information is already extracted leave this question blank and please move to the next domain):**

**Page/paragraph number (e.g., pg. 10/para. 2):**

Domain #9: Preventative Versus Therapy Trials

Did the authors discuss the possible differences or the implications or the appropriateness of the use of ITT in preventative versus therapy treatment trials?	**Y/N?**

**Domain #9: MAIN POINT(s)**
**i. If applicable please copy/paste the paragraph(s) or statement(s) made by the author that you believed to be important, unique or useful regarding this domain:**
**Page/paragraph number (e.g., pg. 10/para. 2):**
**ii. Please provide a brief summary that captures the author's main point(s) that you believe to be most important, unique or useful regarding this domain (if this information is already extracted leave this question blank and please move to the next domain):**
**Page/paragraph number (e.g., pg. 10/para. 2):**

Domain #10: Management Versus Explanatory Treatment Trials

Did the authors discuss the possible differences or the implications or the appropriateness of the use of ITT in management (practical, pragmatic, effectiveness) versus explanatory (mechanistic, efficacy) treatment trials?	**Y/N?**

**Domain #10: MAIN POINT(s)**
**i. If applicable please copy/paste the paragraph(s) or statement(s) made by the author that you believed to be important, unique or useful regarding this domain:**
**Page/paragraph number (e.g., pg. 10/para. 2):**
**ii. Please provide a brief summary that captures the author's main point(s) that you believe to be most important, unique or useful regarding this domain (if this information is already extracted leave this question blank and please move to the next domain):**
**Page/paragraph number (e.g., pg. 10/para. 2):**

Domain #11: Superiority Versus Non-inferiority or Equivalence Trials

Did the authors discuss the possible differences or the implications or the appropriateness of the use of ITT in superiority versus non-inferiority or equivalence treatment trials?<br><br>***If 'No', you are FINISHED with this domain.**<br>**** If 'Yes', and these comments were already provided in domain #4, question 1, you are FINISHED with this domain.**<br>***** If 'Yes', and these comments were NOT provided in domain #4, question 1, you please fill out the following "Main Point(s)" section:**	**Y/N?**

**Domain #11: MAIN POINT(s)**
**i. If applicable please copy/paste the paragraph(s) or statement(s) made by the author that you believed to be important, unique or useful regarding this domain:**
**Page/paragraph number (e.g., pg. 10/para. 2):**
**ii. Please provide a brief summary that captures the author's main point(s) that you believe to be most important, unique or useful regarding this domain (if this information is already extracted leave this question blank and please move to the next domain):**
**Page/paragraph number (e.g., pg. 10/para. 2):**

Domain #12: Missing Completely at Random Versus Missing at Random vs. Missing Nonrandomly

Did the authors discuss the possible differences or the implications or the appropriateness of the use of ITT for data missing completely at random versus missing at random versus missing nonrandomly?<br><br>***If 'No', you are FINISHED with this domain.**<br>**** If 'Yes', and these comments were already provided in domain #4, question 1, you are FINISHED with this domain.**<br>***** If 'Yes', and these comments were NOT provided in domain #4, question 1, you please fill out the following "Main Point(s)" section:**	**Y/N?**

**Domain #12: MAIN POINT(s)**
**i. If applicable please copy/paste the paragraph(s) or statement(s) made by the author that you believed to be important, unique or useful regarding this domain:**
**Page/paragraph number (e.g., pg. 10/para. 2):**
**ii. Please provide a brief summary that captures the author's main point(s) that you believe to be most important, unique or useful regarding this domain (if this information is already extracted leave this question blank and please move to the next domain):**

**Page/paragraph number (e.g., pg. 10/para. 2):**

Domain #13: Proportion of paper devoted to ITT definition?

Approximately how much of the paper is devoted to the discussion of ITT (in pages, e.g. 3 pg)?

Approximately how long is the entire document excluding the references (in pages, e.g. 3 pg)?

APPENDIX C - INCLUDED ARTICLES

Table 1 Included Studies (N=66) with reference number, author, year of publication, journal name and type of journal

Ref. No.	Author	Year	Journal	Type of Journal
22	Akobeng A. K.	2005	Archives of Disease in Childhood	Specialty
23	Yusuf S. et al.	1991	Pacing & Clinical Electrophysiology	Specialty
24	Armitage P.	1998	Statistics in Medicine	Statistics
25	Bailey A. et al.	1994	Blood Review	Specialty
26	Bentzen S. et al.	1998	Radiotherapy and Oncology	Specialty
27	Wright C.C. , Sim J.	2003	Journal of Clinical Epidemiology	Methods
28	Witte S. , Victor N.	2004	Methods of Information Medicine	Methods
29	Winnock M. et al.	2001	Hepatology	Specialty
30	Blackwelder WC	2004	Journal of Dental Research	Specialty
31	Wiens B.L. , Zhao W.	2007	Clinical Trials	Methods
32	Blume J. , Peipert JF.	2004	Journal of the American Association of Gynecologic Laparoscopists	Specialty
33	Borm G.F. et al.	2006	Journal of Clinical Epidemiology	Methods
34	Branson M. , Whitehead J.	2003	Statistics in Medicine	Statistics
35	Bubbar V.K. , Kreder H.J.	2006	Journal of Bone & Joint Surgery	Specialty
36	Whittaker K, et al.	2006	Journal of Epidemiology & Community Health	Specialy
37	White Ian	2005	Statistical Methods in Medical	Statistics

			Research	
38	Weinstein GS , Levin B,	1989	Annals of Thorac Surgery	Specialty
39	Chene G et al.	1998	Controlled clinical trials	Methods
40	D'Agostino RB , Massaro JM	2004	Journal of Dental Research	Specialty
41	Walter SD et al.	2006	Journal of Clinical Epidemiology	Methods
42	Viscoli C et al.	1995	European Journal of Cancer	Specialty
43	Daya Salim	2006	Best Practice & Research in Clinical Obstetrics & Gynaecology	Specialty
44	Irvine EJ et al.	2006	Gastroenterology	Specialty
45	Unnebrink K , Windeler J.	2001	Statistics in Medicine	Statistics
46	Farrington CP	1993	International Journal of Epidemiology	Methods
47	Tillmann H.C et al.	2001	International Journal of Clinical Pharmacology and Therapeutics	Specialty
48	Tierney JF , Stewart LA	2005	International Journal of Epidemiology	Methods
49	Fergusson D et al.	2002	BMJ	General Medicine
50	Fleiss JL	1992	Journal of Periodontal Research	Specialty
51	Freedman DA	2006	Evaluation Review	Methods
52	Furukawa TA et al.	2005	International Clinical Psychopharmacology	Specialty
53	Gibaldi M , Sullivan S	1997	Issues in Clinical Pharmacology	Specialty
54	Glasziou PP	1992	Journal of Clinical Epidemiology	Methods
55	Thoma A.	2005	Clinics in Plastic Surgery	Specialty
56	Streiner D , Geddes J	2001	Evidence Based Mental Health	Specialty

57	Goetghebeur E , Loeys T	2002	Epidemiologic Reviews	Methods
58	Gravel J et al.	2007	Clinical Trials	Methods
59	Green SB	2000	Oncology Clinics of North America	Specialty
60	Sorensen HT et al.	2006	Hepatology	Specialty
61	Soares I , Carneiro AV	2002	Cardiologia Baseada Na Evidencia	Specialty
62	Newell	1992	International Journal of Epidemiology	Methods
63	Sheiner LB , Rubin DB	1995	Clinical Pharmacology & Therapeutics	Specialty
64	Shao J, Zhong B.	2003	Statistics in Medicine	Statistics
65	Hollis S.	2002	Statistics in Medicine	Statistics
66	Hollis S , Campbell F	1999	BMJ	General Medicine
67	Schoenfeld PS	2005	American Journal of Gastroenterology	Specialty
68	Sato Tosiya	2001	Statistics Medicine	Statistics
69	Jones B et al.	1996	BMJ	General Medicine
70	Sabin CA et al.	2000	HIV Clinical Trials	Specialty
71	Ruiz-Canela M et al.	2000	BMJ	General Medicine
72	Kleinman KP et al.	1998	Biometrics	Statistics
73	Korhonen PA	1999	Statistics Medicine	Statistics
74	Kruse RL et al.	2002	Journal of Family Practice.	Specialty
75	Porta N. et al.	2007	Journal of Clinical Epidemiology	Methods
76	Peace Karl E , Carter	1993	Journal of Biopharmaceutical	Statistics

	Hans Jr		Statistics	
77	Nich C , Carroll KM	2002	Drug and Alcohol Dependence	Specialty
78	Law MG , Kaldor JM	1996	Statistics In Medicine	Statistics
79	Lewis	1993	Br J Cancer	Specialty
80	Newcombe RG	1988	Statistics In Medicine	Statistics
81	Loeys T , Goetghebeur E	2003	Biometrics	Statistics
82	Nagelkerke N, Fidler V, Bernsen R, Borgdorff M.	2000	Statistics In Medicine	Statistics
83	Montori VM , Guyatt GH	2001	Canadian Medical Association Journal	General Medicine
84	Lui KJ	2007	Statistics In Medicine	Statistics
85	Mahaniah KJ , Rao G	2004	The Journal of Family Practice	Specialty
86	Mittlbock M , Whitehead J	1998	Lifetime Data Analysis	Statistics
87	Altman,Douglas G et al.	2001	Annals of Internal Medicine	General Medicine

APPENDIX D - AUTHOR CONTRIBUTIONS

Study concept and design:

Akl, Alshurafa, Haines, Guyatt, Walter

Acquisition of data:

Akl, Alshurafa, Haines, Gentles, Guyatt, Walter, Rios, Tran

Analysis and interpretation of data:

Alshurafa, Haines, Gentles, Guyatt, Walter, Rios, Tran

Drafting of the manuscript:

Alshurafa

Critical revision of the manuscript for important intellectual content:

Alshurafa, Gentles, Guyatt, Walter

Statistical expertise:

Alshurafa, Guyatt, Walter

Administrative, technical, or material support:

Alshurafa, Guyatt, Haines

Study supervision:

Guyatt, Haines, Walter, Moayyedi

APPENDIX E - BASIC DEFINITION OF ITT

Figure 1 Articles that provide a basic definition of ITT and give no comment on ITT in relation to LTFU. About half the articles use 'All' in their definition of ITT.

**Author**	**Basic definition of ITT provided**	**Comments on LTFU**
Peace[46]	*"ITT analyses are interpreted here as analyses which include **all** patients who are randomized but which do not factor in or adjust for differential exposure to study medications." – pg 130*	Provided no comment on LTFU in relation to ITT
Akobeng[27]	*"**All** patients allocated to either the treatment or control groups are analyzed together as representing that treatment arm"*- pg 842	Provided no comment on LTFU in relation to ITT
Bentzen[29]	*"**All** randomized patients should be included in the primary analysis of a RCT". – pg 5*	Provided no comment on LTFU in relation to ITT

APPENDIX F – AUTHORITIES ON THE ITT DEFINITION

Authors of methodology papers have identified a variety of 'authorities' when it comes to the verification of the definition of the ITT principle. These 'authorities' were often referenced or quoted when defining ITT. Unfortunately, it appears that the authorities themselves have differing definitions of ITT. This may have added to the discrepancies in the definition of the term. Here the positions of the various authorities on the definition of ITT in relation to LTFU are summarized:

Authority #1 - The grandfather 'authority' – Austin Bradford Hill:

As previously mentioned, Hill first used the term in the 7th edition of his 1961 textbook entitled "Principles of Medical Statistics" (See **Figure 1**). Hill's intention was to prevent to bias resulting from analysing participants in the treatment they actually took (as opposed to the treatment they were originally randomized to). He called for maintaining the "*careful balance, originally secured by randomisation"* by avoiding bias through exclusion after randomization according to non-receipt of treatment. Nowhere does Hill state that LTFU violates ITT nor does he specify a certain method of analysis for handling those with LTFU under ITT. Thus, Hill failed to comment on the relation of LTFU with ITT and this could be the historical reason for the present ambiguity in the term.[1]

In our review, CONSORT was one of the most frequently cited authorities on the definition of ITT by authors published in the last decade. The 2001 CONSORT strongly advocated for the use of ITT in RCTs[2]. Thus, extraction of this article was of particular importance. Our review of the 2001 CONSORT Statement concluded that it was completely ambiguous with regards to its definition of ITT. More specifically, it mentioned all three definitions for ITT and provided no preference towards any one definition over the other.

Since then, CONSORT has recently updated its statement. It no longer advocates for the use ITT. The 2010 CONSORT statement partly clarified its position on what constitutes ITT, but some ambiguity remains in their definition[3]. Some changes include the removal of the following sentence in pg 681 which seems to imply a complete-case analysis is possible under ITT:

> *"Although those participants cannot be included in the analysis, it is customary still to refer to analysis of all available participants as an intention-to-treat analysis"*[4]

In the 2010 version CONSORT makes clear that a 'strict' ITT is only achieved with 100% follow-up but also allows for imputation.

> *"Strict intention-to-treat analysis is often hard to achieve for two main reasons—missing outcomes for some participants and non-adherence to the trial protocol… Many trialists exclude patients without an observed outcome. Often this is reasonable, but once any randomised participants are excluded the analysis is not strictly an intention-to-treat analysis... Participants with missing outcomes can be included in the analysis only if their outcomes are*

imputed (that is, their outcomes are estimated from other information that was collected). Imputation of the missing data allows the analysis to conform to intention-to-treat analysis but requires strong assumptions, which may be hard to justify”

This position that imputation of missing data is acceptable under ITT but not 'strictly' ITT is confusing. However, it clarifies that a complete-case analysis and any post-randomization exclusions completely violate ITT.

CONSORT decided not to request the use of the term ITT in the 2010 CONSORT Statement. CONSORT does not request the use of the term ITT anymore because it is 'widely misused' and not 'reliable'.

The 2010 CONSORT Statement Explanation and Elaboration explains:

“Like "intention-to-treat," none of these other labels reliably clarifies exactly which patients were included. Thus, in the CONSORT checklist we have dropped the specific request for intention-to-treat analysis in favour of a clear description of exactly who was included in each analysis…
We replaced mention of “Intention to treat” analysis, a widely misused term, by a more explicit request for information about retaining participants in their original assigned groups.[5][1] *“*

This paper agrees that ITT is not a reliable way to determine which patients were included. However, the issue is not so much that ITT is 'widely misused' – rather it has

[1] Schulz KF; Altman DG; Moher D,CONSORT 2010 **statement: updated guidelines for reporting parallel group randomized trials.** *Ann Intern Med* - 1-JUN-2010; 152(11): 726-32.

been poorly defined as there was never a consensus on the definition to begin with. Similar to CONSORT, the Cochrane handbook, a major authority on statistical methodology, has seen a recent change in direction with regards to their definition of ITT. Early Cochrane views indicated full follow-up was required and imputation was possible within the definition[6]. Later, in the 2008 Cochrane handbook, it seemed to allow for the possibility of all three definitions and less strict on us of a complete case analysis.[7,8]

Figure 1 The first mention of the term intention to treat by Bradford Hill (1961)

Differential Exclusions

Before analysing the results of a trial there is another vital question to consider – have any patients *after admission* to the treated or control group been excluded from further observation ? Such exclusions may affect the validity of the comparisons that it is sought to make; for they may *differentially* affect the two groups. For instance, suppose that certain patients cannot be retained on a drug – perhaps through toxic side-effects. No such exclusions will occur on the placebo and the careful balance, originally secured by randomisation, may thereby be disturbed. Another specific example might lie in a trial of pneumonectomy versus radiation in the treatment of cancer of the lung (supposing such a trial to be ethically possible). At operation there is no doubt that pneumonectomy would sometimes be found impossible to perform and it would seem only sensible to exclude these patients. But we must observe that no such exclusions can take place in the group treated by radiation. If we exclude such patients on the one side and inevitably retain them on the other, can we any longer be sure that we have two comparable groups differentiated only by treatment ? Unless the losses are very few and therefore unimportant, we may inevitably have to keep such patients in the comparison and thus measure the *intention to treat* in a given way rather than the actual treatment. The question of the introduction of bias through exclusions for any reason (including lost sight of) must, therefore, always be carefully studied, *not only at the end of a trial but throughout its progress.* This continuous care is essential in order that we may immediately consider the nature of the exclusions and whether they must be retained in inquiry for follow-up, measurement, etc. It will be too late to decide about that at the end of the trial. – pg 258-259

- A. Bradford Hill, Principles of Medical Statistics, 7th edition, Oxford University Press, 1961.

References

[1] Bradford Hill, A., Principles of Medical Statistics, 7th edition, Oxford University Press, 1961, page 258.

[2] Altman,DG, Schulz, KF, Moher, D., Egger, M., Davidoff, F. et al; "The Revised CONSORT Statement for Reporting Randomized Trials: Explanation and Elaboration", Annals of Internal Medicine, 2001, 134:663-694.

[3] Moher D, Hopewell S, Schulz KF, Montori V, Gøtzsche PC, Devereaux PJ, Elbourne D, Egger M, Altman DG; Consolidated Standards of Reporting Trials Group. "CONSORT 2010 Explanation and Elaboration: Updated guidelines for reporting parallel group randomised trials." J Clin Epidemiol. 2010 Aug;63(8):e1-37. Epub 2010 Mar 25.

[4] Moher, D., Hopewell, S., Schulz, KF., Montori, V., et al. "CONSORT 2010 Explanation and Elaboration: Updated guidelines for reporting parallel group randomized trials." BMK, 2010: 340:c869, doi: 10.1136/bmj.c869

[5] Schulz KF; Altman DG; Moher D,CONSORT 2010 statement: updated guidelines for reporting parallel group randomized trials. Ann Intern Med - 1-JUN-2010; 152(11): 726-32.

[6] Personal Communication, G.G., 02/05/2008 email

[7] http://books.google.ca/books?id=s11gCx4LS_EC&pg=PT510&lpg=PT510&dq=cochrane+intention+to+treat&source=bl&ots=FeOvt0ru-#v=onepage&q=cochrane%20intention%20to%20treat&f=false (Accessed 9/2010)

[8] http://www.cochrane-net.org/openlearning/HTML/mod14-4.htm (Accessed 9/2010)

APPENDIX G - DEFINITION OF ITT

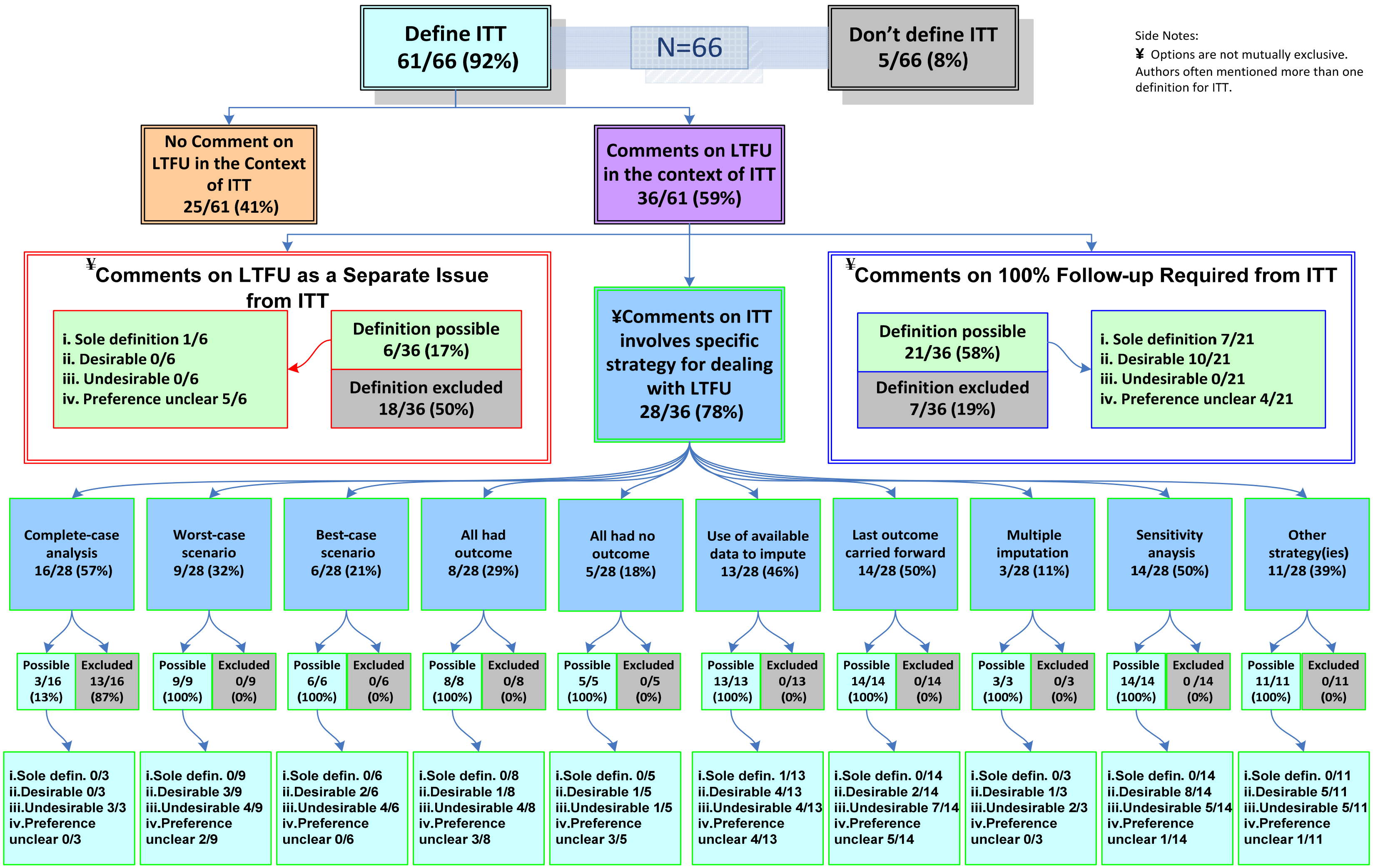

Supplement: Appendix S1 — MSc thesis of MA including study protocol. (PDF) [file pone.0049163.s001.pdf]
