# Supplementary material for: Inconsistent Definitions for Intention-To-Treat in Relation to Missing Outcome Data: Systematic Review of the Methods Literature
Source: PLoS One. 2012 Nov 15;7(11):e49163. doi: 10.1371/journal.pone.0049163 (PMC3499557; doi:10.1371/journal.pone.0049163)
Supplement: Appendix S2 — MEDLINE search strategy. (DOC) [file pone.0049163.s002.doc]

**Appendix S2 – Search Strategy**

**Search Strategy Used in Review**

**OVID MEDLINE(R):**

1 intention to treat.ti.

2 intention to treat.mp.

3 intent to treat.mp.

4 intent to treat.ti.

5 1 or 4

6 2 or 3

7 exp Randomized Controlled Trials as Topic/

8 exp clinical trials as topic/

9 research design/

10 exp Data Interpretation, Statistical/

11 sensitivity analysis.mp.

12 complete case analysis.mp.

13 as treated analysis.mp.

14 per protocol analysis.mp.

15 worst case scenario.mp.

16 worse case scenario.mp.

17 locf.mp.

18 best case scenario.mp.

19 multiple imputation.mp.

20 last observation carried.mp.

21 (explanatory analysis or efficacy analysis or analysis by treatment).mp.

22 or/7-21

23 6 and 22

24 23 or 5
