# Supplementary material for: Inconsistent Definitions for Intention-To-Treat in Relation to Missing Outcome Data: Systematic Review of the Methods Literature
Source: PLoS One. 2012 Nov 15;7(11):e49163. doi: 10.1371/journal.pone.0049163 (PMC3499557; doi:10.1371/journal.pone.0049163)
Supplement: Appendix S3 — Data extraction forms. (DOC) [file pone.0049163.s003.doc]

**INTENTION TO TREAT (ITT) Study**

***Data Abstraction Form*** *(version 16.6)*

| **R****eviewer initials (e.g., M.A.)** |  |
| --- | --- |
| **3-digit Study ID (e.g., 052)** |  |
| **Author (Last name, first name)** |  |
| **Year (e.g., 2003)** |  |
| **Journal (e.g. JAMA)** |  |

**Domain #1: Definition of ITT provided by the paper.**

| **1) The paper gives a preferred or possible definition of ITT (please select ‘Yes’ or ‘No’):**  *** If ‘No’, go to next domain (i.e., you are FINISHED with this domain).**  **** If ‘Yes’, answer question 2a.** | | | |
| --- | --- | --- | --- |
| **2a) The paper comments on the definition of ITT AND includes some comment about loss to follow-up (LTFU) in the definition of ITT:**  *** If ‘No’ to ‘2a’, answer question 2b and then you are FINISHED with domain #1.**  **** If ‘Yes’ to ‘2a’, select ALL that apply from definitions 1 to 4 in green (below):** | | **2b) The paper defines ITT as the comparison of:**  *** If you answered ‘No’ to question ‘2a’, after you answer ‘2b’, you are FINISHED with domain #1. Please go to domain #2 “Definition of Modified ITT Provided by Paper” on pg 5.** | |
| **Important notes before filling out Definitions 1 to 4:**  **i. If you answered ‘yes’ to question #1 AND ‘yes’ to question #2a you MUST choose an answer for each definition below (ie. Definition 1 to 4).**  **ii**. An author can ‘endorse’ more than one definition for ITT (e.g. Can endorse definition 1 and definition 2).  **iii**. You MUST answer the ‘CLARITY Clarification’ section at the bottom of Domain #1. | | | |
| **Definition 1**: Comparison of ALL participants as randomized for which investigators have recorded the outcome of interest, regardless of protocol deviations and participant compliance: **For participants for whom outcome data is unavailable (i.e. LTFU) investigators MUST do the following (check all that apply):** | | | VERY IMPORTANT NOTE for definition 1:  *If you chose any of options i-iv for definition 1 you MUST answer all of 1a-1j below.  *If you chose option v “Definition not mentioned by author” go to Definition 2 in domain #1. |
|  | 1a. **Complete case analysis**: exclude from the analysis. | | *if this is a mandatory option for ITT than fill out ‘definition specifically excluded’ for definition #3 below. |
|  | 1b. **Worst case scenario**: assume all participants in the treatment group had the event and none in the control had it. | |  |
|  | 1c. **Best case scenario**: assume all participants in treatment group did NOT have the event and all in the control had it. | |  |
|  | 1d. **All had outcome**: assume all those LTFU had suffered the outcome of interest. | |  |
|  | 1e. **All had no outcome**: assume none of those LTFU had suffered the outcome of interest. | |  |
|  | 1f. **Use of available data to impute missing outcome**: available information on participants LTFU is used to assign outcome. | |  |
|  | 1g. **Last outcome carried forward**: last observed response in participants LTFU is used to assign the outcome. | |  |
|  | 1h. **Multiple imputation techniques** | |  |
|  | 1i. **Sensitivity analysis**: testing more than one assumption and look at implications of alternative assumptions. | |  |
|  | 1j. **Other** (please specify): | |  |
| **Definition 2:** Comparison of ALL participants as randomized for which investigators have recorded the outcome of interest, regardless of protocol deviations and participant compliance. **For participants for whom outcome data is unavailable (i.e. LTFU) investigators MAY do the following (check all that apply):** | | | *If option v “Definition not mentioned by author” is selected go to definition 3 in domain #1. Otherwise answer a-j. |
|  | 2a. **Complete case analysis**: exclude from the analysis. | |  |
|  | 2b. **Worst case scenario**: assume all participants in the treatment group had the event and none in the control had it. | |  |
|  | 2c. **Best case scenario**: assume all participants in treatment group did NOT have the event and all in the control had it. | |  |
|  | 2d. **All had outcome**: assume all those LTFU had suffered the outcome of interest. | |  |
|  | 2e. **All had no outcome**: assume none of those LTFU had suffered the outcome of interest. | |  |
|  | 2f. **Use of available data to impute missing outcome**: available information on participants LTFU is used to assign outcome. | |  |
|  | 2g. **Last outcome carried forward**: last observed response in participants LTFU is used to assign the outcome. | |  |
|  | 2h. **Multiple imputation techniques** | |  |
|  | 2i. **Sensitivity analysis**: testing more than one assumption and look at implications of alternative assumptions. | |  |
|  | 2j. Other (please specify): | |  |
| **Definition 3:** Comparison of ALL participants as randomized for which investigators have recorded the outcome of interest, regardless of protocol deviations and participant compliance: **ITT cannot be done if LTFU occurred (i.e. FULL FOLLOW-UP IS REQUIRED).** | | |  |
| **Definition 4:** Comparison of ALL participants as randomized for which investigators have recorded the outcome of interest, regardless of protocol deviations and participant compliance: **ITT is a SEPARATE ISSUE from LTFU.** | | |  |

| **3) CLARITY Clarification:**  Comparison of ALL participants as randomized for which investigators have recorded the outcome of interest, regardless of protocol deviations and participant compliance: **It is UNCLEAR whether the authors preferred to impute or exclude patients (i.e., Definition 1 or 2) for ITT, whether they prefer ‘FULL FOLLOW-UP IS REQUIRED’ to be equivalent to ITT (i.e., Definition 3), or whether they prefer ITT is a ‘SEPARATE ISSUE from LTFU’ (Definition 4).** |  |
| --- | --- |

| **Domain #1: MAIN POINT(s)** |
| --- |
| **i. If applicable please copy/paste the paragraph(s) or statement(s) made by the author that you believed to be important, unique or useful regarding this domain:** |
| **Page/paragraph number (e.g., pg. 10/para. 2):** |
|  |
| **ii. Please provide a brief summary that captures the author’s main point(s) that you believe to be most important, unique or useful regarding this domain (if this information is already extracted leave this question blank and please move to the next domain):** |
| **Page/paragraph number (e.g., pg. 10/para. 2):** |

*** The rest of the data abstraction is to be filled using the author’s preferred definition in Domain #1.**

**Domain #2: Definition of modified-ITT provided by the paper**

| **1.** Does the paper define **modified-ITT**?  ***If ‘No’, you are FINISHED with this domain.**  ****If ‘Yes’, answer ALL of the following question(s):**  The paper **defined modified-ITT** as one of the following (check all that apply, Definition 1 and/or Definition 2): | | |  |
| --- | --- | --- | --- |
| **Definition 1**: Participants are analyzed as randomized BUT some participants are EXCLUDED (check all that apply i-iv): | | |  |
|  | i) **Post-randomization exclusion**: participants randomized but **never received** the allocated therapy: | |  |
|  | | a. Appropriate if patients were blinded to allocation NOT related to either the allocated intervention or outcomes. |  |
|  | | b. Decision of excluding should be made on the basis of information NOT related to either the allocated intervention or outcomes. |  |
|  | | c. None specified. |  |
|  | | d. **Other** criteria related to participants who never received the allocated therapy (please specify): |  |
|  | ii) **Post-randomization exclusion**: participants who **withdrew** their consent but are not LTFU: | |  |
|  | | a. Decision of excluding should be made on the basis of information NOT related to either the allocated intervention or outcomes. |  |
|  | | b. None specified. |  |
|  | | c. **Other** criteria related to participants who **withdrew** their consent but are not LTFU (please specify): |  |
|  | iii) **Post-randomization exclusion**: Ineligible participants who are **mistakenly randomized:** | |  |
|  | | a. Individual that makes decision to exclude (i.e. adjudicator) is blinded to treatment. |  |
|  | | b. None specified. |  |
|  | | c. Other criteria related to ineligible participants who are **mistakenly randomized** (please specify): |  |
|  | iv) **Post-randomization exclusion**: participants excluded because of **center exclusion**: | |  |
|  | | a. None specified. |  |
|  | | b. Other criteria related to participants excluded because of **center exclusion. P**lease specify: |  |
|  | v) Participants LTFU. | |  |
|  | vi) Other than **never received, withdrew, mistakenly randomized or center exclusion**. Please specify: | |  |
| **Definition 2**: Other definition of modified-ITT that DOESN’T involve exclusion of participants (e.g., analytic strategies). Please specify: | | |  |

| **Domain #2: MAIN POINT(s)** |
| --- |
| **i. If applicable please copy/paste the paragraph(s) or statement(s) made by the author that you believed to be important, unique or useful regarding this domain:** |
| **Page/paragraph number (e.g., pg. 10/para. 2):** |
|  |
| **ii. Please provide a brief summary that captures the author’s main point(s) that you believe to be most important, unique or useful regarding this domain (if this information is already extracted leave this question blank and please move to the next domain):** |
| **Page/paragraph number (e.g., pg. 10/para. 2):** |

**Domain #3: Recommendation for dealing with LTFU provided by the paper outside of the context of ITT**

| Does the paper make a recommendation for how to deal with LTFU outside of the context of ITT?  ***If ‘No’, you are FINISHED with this domain.**  ****If ‘Yes’, answer ALL of the following question(s):** | | |  |
| --- | --- | --- | --- |
| The paper recommends that one deals with LTFU assuming the following [check all that apply (a-k)]: | | | |
|  | a. **Complete case analysis**: exclude from the analysis. | |  |
|  | b. **Worst case scenario**: assume all participants in the treatment group had the event and none in the control had it. | |  |
|  | c. **Best case scenario**: assume all participants in treatment group did NOT have the event and all in the control had it. | |  |
|  | d. **All had outcome**: assume all those LTFU had suffered the outcome of interest. | |  |
|  | e. **All had no outcome**: assume none of those LTFU had suffered the outcome of interest. | |  |
|  | f. **Use of available data**: available information on participants LTFU is used to assign outcome. | |  |
|  | g. **Last outcome carried forward**: last observed response in participants LTFU is used to assign the outcome. | |  |
|  | h. **Multiple imputation techniques.** | |  |
|  | i. **Sensitivity analysis**: testing more than one assumption and look at implications of alternative assumptions. | |  |
|  | | **Were specific sensitivity analyses recommended?**  **If yes, list them:** |  |
|  | j. **Minimize LTFU.** | |  |
|  | k. **Other** (please specify): | |  |
|  | l. **Unclear**. | |  |

| **Domain #3: MAIN POINT(s)** |
| --- |
| **i. If applicable please copy/paste the paragraph(s) or statement(s) made by the author that you believed to be important, unique or useful regarding this domain:** |
| **Page/paragraph number (e.g., pg. 10/para. 2):** |
|  |
| **ii. Please provide a brief summary that captures the author’s main point(s) that you believe to be most important, unique or useful regarding this domain (if this information is already extracted leave this question blank and please move to the next domain):** |
| **Page/paragraph number (e.g., pg. 10/para. 2):** |

**Domain #4: Limitations of ITT (using the papers preferred definition of ITT, if applicable)**

| Does the paper provide limitations of the ITT principle?  ***If ‘No’, you are FINISHED with this domain.**  ****If ‘Yes’, answer ALL of the following question(s):** | | |  |
| --- | --- | --- | --- |
| 1. The paper suggests that the bias depends on whether it is superiority vs. inferiority/equivalence.  If yes, check all that apply: | | |  |
|  | **Definitely yes** | **Probably yes** | |
| i. In the case of ITT, superiority bias toward underestimation of effect. |  |  | |
| ii. In the case of ITT, noninferiority/equivalence bias toward showing noninferiority/equivalence. |  |  | |
| iii. Other, please specify (e.g. selection bias): |  | | |

| **Domain #4: MAIN POINT(s)** |
| --- |
| **i. If applicable please copy/paste the paragraph(s) or statement(s) made by the author that you believed to be important, unique or useful regarding this domain:** |
| **Page/paragraph number (e.g., pg. 10/para. 2):** |
|  |
| **ii. Please provide a brief summary that captures the author’s main point(s) that you believe to be most important, unique or useful regarding this domain (if this information is already extracted leave this question blank and please move to the next domain):** |
| **Page/paragraph number (e.g., pg. 10/para. 2):** |

**Domain #5: When exclusion does not convey bias or when groups are omitted and are still consistent with ITT.**

| **1. Author addresses exclusion without bias.**  ***If ‘No’, you are FINISHED with this domain.**  ****If ‘Yes’, answer ALL of the following question(s):** | | |  |
| --- | --- | --- | --- |
| **2. Author mentions exclusion without bias and…**  **… consistent with ITT.** | | | |
| **3. Decision to exclude patient conveys no bias if participants are analyzed as randomized but some participants are excluded**  ***If ‘No’, you are FINISHED with this domain.**  ****If ‘Yes’, check all that apply i-iv:** | | |  |
|  | i) **Post-randomization exclusion**: participants randomized but **never received** the allocated therapy (if there are any additional details please specify and/or check all that apply): | |  |
|  | | a. Appropriate if patients were blinded to allocation. |  |
|  | | b. Decision of excluding should be made on the basis of information NOT related to either the allocated intervention or outcomes. |  |
|  | | c. None specified. |  |
|  | | d. **Other** criteria related to participants who never received the allocated therapy (please specify): |  |
|  | ii) **Post-randomization exclusion**: Ineligible participants who are **mistakenly randomized** (if there are any additional details please specify and/or check all that apply): | |  |
|  | | a. If the individual that makes decision to exclude (i.e. adjudicator) is blinded to treatment. |  |
|  | | b. None specified. |  |
|  | | c. Other criteria related to ineligible participants who are **mistakenly randomized**. Please specify: |  |
|  | iii) **Post-randomization exclusion**: participants excluded because of study **center exclusion** (if there are any additional details please specify and/or check all that apply): | |  |
|  | | a. None specified. |  |
|  | | b. Other criteria related to participants excluded because of **center exclusion** Please specify: |  |
|  | iv) Other than **never received, withdrew, mistakenly randomized or center exclusion**. Please specify: | |  |
| 4. Author states that ANY post-randomization exclusions may introduce bias. | | |  |

| **Domain #5: MAIN POINT(s)** |
| --- |
| **i. If applicable please copy/paste the paragraph(s) or statement(s) made by the author that you believed to be important, unique or useful regarding this domain:** |
| **Page/paragraph number (e.g., pg. 10/para. 2):** |
|  |
| **ii. Please provide a brief summary that captures the author’s main point(s) that you believe to be most important, unique or useful regarding this domain (if this information is already extracted leave this question blank and please move to the next domain):** |
| **Page/paragraph number (e.g., pg. 10/para. 2):** |

**Domain #6: Biases that arise if NOT ITT (using the papers preferred definition of ITT, if applicable)**

Does the paper suggest biases that will result due to running analysis other than ITT?

***If ‘No’, you are FINISHED with this domain.**

****If ‘Yes’, answer ALL of the following question(s):**

1. The paper suggests the following biases will result due to running analysis other than ITT (check all that apply and answer yes or no to questions 1-6):

| **i. Overestimate the effect:** | **Definitely yes** | **Probably yes** |
| --- | --- | --- |
| a) Overestimates the benefit (false positive). |  |  |
| b) Overestimate the harm. |  |  |
| c) Not specified whether harm or benefit. |  |  |
|  |  |  |
| **ii. Underestimate the effect:** | **Definitely yes** | **Probably yes** |
| a) Underestimate the benefit (false negative). |  |  |
| b) Underestimate the harm. |  |  |
| c) Not specified whether harm or benefit |  |  |

2. Bias, explicit statement that direction of bias can not be predicted.

3. Bias, no explicit direction specified.

| **Domain #6: MAIN POINT(s)** |
| --- |
| **i. If applicable please copy/paste the paragraph(s) or statement(s) made by the author that you believed to be important, unique or useful regarding this domain:** |
| **Page/paragraph number (e.g., pg. 10/para. 2):** |
|  |
| **ii. Please provide a brief summary that captures the author’s main point(s) that you believe to be most important, unique or useful regarding this domain (if this information is already extracted leave this question blank and please move to the next domain):** |
| **Page/paragraph number (e.g., pg. 10/para. 2):** |

**Domain #7: Withdrawl of consent to participate in trial.**

| **Do the authors discuss ethical issues of withdrawl of consent (i.e. the ethical imperative to exclude such patients despite data being available)?**  ***If ‘No’, you are FINISHED with this domain.**  ****If ‘Yes’, answer ALL of the following question(s):** | |  |
| --- | --- | --- |
| **Do the authors mention any of the following:** | |  |
|  | a. There is an ethical imperative to exclude patients that withdraw consent despite data being available (i.e. one may not use data because consent was withdrawn).  If additional information is given please specify: |  |
|  | b. There is NO ethical imperative to exclude patients that withdraw consent despite data being available (i.e. one may use data even though consent was withdrawn).  If additional information is given please specify: |  |

| **Domain #7: MAIN POINT(s)** |
| --- |
| **i. If applicable please copy/paste the paragraph(s) or statement(s) made by the author that you believed to be important, unique or useful regarding this domain:** |
| **Page/paragraph number (e.g., pg. 10/para. 2):** |
|  |
| **ii. Please provide a brief summary that captures the author’s main point(s) that you believe to be most important, unique or useful regarding this domain (if this information is already extracted leave this question blank and please move to the next domain):** |
| **Page/paragraph number (e.g., pg. 10/para. 2):** |

**Domain #8: Recommendation to achieve full follow-up regardless of definition of ITT**

| Does the paper make recommendations to achieve full follow-up?  ***If ‘No’, you are FINISHED with this domain.**  ****If ‘Yes’, answer ALL of the following question(s):** | | |  |
| --- | --- | --- | --- |
| The paper recommends the following strategies to achieve full follow-up (check all that apply): | | |  |
|  | 1) Avoid eligibility errors. | |  |
|  | **2**) Minimize ‘dropouts’ from treatment. | |  |
|  | 3) Minimize crossover of participants between groups. | |  |
|  | 4) Minimize the post-randomization exclusions. | |  |
|  | 5) Excluding participants that may be hard to follow-up: | |  |
|  | | a) No fixed addresses |  |
|  | | b) are intellectually handicapped |  |
|  | | b) Other (please specify): |  |
|  | 6) Having a ‘Tracker’ dedicated to finding the outcome of participants that lost contact. | |  |
|  | 7) Thorough consent process (eg. Be clear about tribulations of participating and obtain consent to track participant). | |  |
|  | 8) Request detailed contact information of participant and/or relatives | |  |
|  | 9) Education of investigators. | |  |
|  | 10) Ongoing clinical support during the trial. | |  |
|  | 11) Using simple outcomes (e.g. death). | |  |
|  | 12) Continue to follow-up even after ‘dropout’ from treatment. | |  |
|  | 13) Using an active run- in phase. | |  |
|  | 14) Other (please specify): | |  |

| **Domain #8: MAIN POINT(s)** |
| --- |
| **i. If applicable please copy/paste the paragraph(s) or statement(s) made by the author that you believed to be important, unique or useful regarding this domain:** |
| **Page/paragraph number (e.g., pg. 10/para. 2):** |
|  |
| **ii. Please provide a brief summary that captures the author’s main point(s) that you believe to be most important, unique or useful regarding this domain (if this information is already extracted leave this question blank and please move to the next domain):** |
| **Page/paragraph number (e.g., pg. 10/para. 2):** |

**Domain #9: Preventative Versus Therapy Trials**

| Did the authors discuss the possible differences or the implications or the appropriateness of the use of ITT in preventative versus therapy treatment trials? |  |
| --- | --- |

| **Domain #9: MAIN POINT(s)** |
| --- |
| **i. If applicable please copy/paste the paragraph(s) or statement(s) made by the author that you believed to be important, unique or useful regarding this domain:** |
| **Page/paragraph number (e.g., pg. 10/para. 2):** |
|  |
| **ii. Please provide a brief summary that captures the author’s main point(s) that you believe to be most important, unique or useful regarding this domain (if this information is already extracted leave this question blank and please move to the next domain):** |
| **Page/paragraph number (e.g., pg. 10/para. 2):** |

**Domain #10: Management Versus Explanatory Treatment Trials**

| Did the authors discuss the possible differences or the implications or the appropriateness of the use of ITT in management (practical, pragmatic, effectiveness) versus explanatory (mechanistic, efficacy) treatment trials? |  |
| --- | --- |

| **Domain #10: MAIN POINT(s)** |
| --- |
| **i. If applicable please copy/paste the paragraph(s) or statement(s) made by the author that you believed to be important, unique or useful regarding this domain:** |
| **Page/paragraph number (e.g., pg. 10/para. 2):** |
|  |
| **ii. Please provide a brief summary that captures the author’s main point(s) that you believe to be most important, unique or useful regarding this domain (if this information is already extracted leave this question blank and please move to the next domain):** |
| **Page/paragraph number (e.g., pg. 10/para. 2):** |

**Domain #11: Superiority Versus Non-inferiority or Equivalence Trials**

| Did the authors discuss the possible differences or the implications or the appropriateness of the use of ITT in superiority versus non-inferiority or equivalence treatment trials?  ***If ‘No’, you are FINISHED with this domain.**  **** If ‘Yes’, and these comments were already provided in domain #4, question 1, you are FINISHED with this domain.**  ***** If ‘Yes’, and these comments were NOT provided in domain #4, question 1, you please fill out the following “Main Point(s)” section:** |  |
| --- | --- |

| **Domain #11: MAIN POINT(s)** |
| --- |
| **i. If applicable please copy/paste the paragraph(s) or statement(s) made by the author that you believed to be important, unique or useful regarding this domain:** |
| **Page/paragraph number (e.g., pg. 10/para. 2):** |
|  |
| **ii. Please provide a brief summary that captures the author’s main point(s) that you believe to be most important, unique or useful regarding this domain (if this information is already extracted leave this question blank and please move to the next domain):** |
| **Page/paragraph number (e.g., pg. 10/para. 2):** |

**Domain #12: Missing Completely at Random Versus Missing at Random vs. Missing Nonrandomly**

| Did the authors discuss the possible differences or the implications or the appropriateness of the use of ITT for data missing completely at random versus missing at random versus missing nonrandomly?  ***If ‘No’, you are FINISHED with this domain.**  **** If ‘Yes’, and these comments were already provided in domain #4, question 1, you are FINISHED with this domain.**  ***** If ‘Yes’, and these comments were NOT provided in domain #4, question 1, you please fill out the following “Main Point(s)” section:** |  |
| --- | --- |

| **Domain #11: MAIN POINT(s)** |
| --- |
| **i. If applicable please copy/paste the paragraph(s) or statement(s) made by the author that you believed to be important, unique or useful regarding this domain:** |
| **Page/paragraph number (e.g., pg. 10/para. 2):** |
|  |
| **ii. Please provide a brief summary that captures the author’s main point(s) that you believe to be most important, unique or useful regarding this domain (if this information is already extracted leave this question blank and please move to the next domain):** |
| **Page/paragraph number (e.g., pg. 10/para. 2):** |

**Domain #13: Proportion of paper devoted to ITT definition?**

Approximately how much of the paper is devoted to the discussion of ITT (in pages, e.g. 3 pg)?

**Approximately how long is the entire document excluding the references (in pages, e.g. 3 pg)?**
