# Supplementary material for: Inconsistent Definitions for Intention-To-Treat in Relation to Missing Outcome Data: Systematic Review of the Methods Literature
Source: PLoS One. 2012 Nov 15;7(11):e49163. doi: 10.1371/journal.pone.0049163 (PMC3499557; doi:10.1371/journal.pone.0049163)
Supplement: Appendix S4 — Detailed results and list of included studies (DOC) [file pone.0049163.s004.doc]

**Appendix S4 – List of included articles and details of results**

| **Ref. No.** | **Author** | **Year** | **Journal** | **Type of Journal** |
| --- | --- | --- | --- | --- |
| 1 | Akobeng AK | 2005 | Archives of Disease in Childhood | Specialty |
| 2 | Yusuf S. et al. | 1991 | Pacing & Clinical Electrophysiology | Specialty |
| 3 | Armitage P | 1998 | Statistics in Medicine | Statistics |
| 4 | Bailey A et al. | 1994 | Blood Review | Specialty |
| 5 | Bentzen SM | 1998 | Radiotherapy and Oncology | Specialty |
| 6 | Wright CC et al. | 2003 | Journal of Clinical Epidemiology | Methods |
| 7 | Witte S et al. | 2004 | Methods of Information Medicine | Methods |
| 8 | Winnock M et al. | 2001 | Hepatology | Specialty |
| 9 | Blackwelder WC | 2004 | Journal of Dental Research | Specialty |
| 10 | Wiens BL et al. | 2007 | Clinical Trials | Methods |
| 11 | Blume J et al. | 2004 | Journal of the American Association of Gynecologic Laparoscopists | Specialty |
| 12 | Borm GF et al. | 2006 | Journal of Clinical Epidemiology | Methods |
| 13 | Branson M et al. | 2003 | Statistics in Medicine | Statistics |
| 14 | Bubbar VK et al. | 2006 | Journal of Bone & Joint Surgery | Specialty |
| 15 | Whittaker K, et al. | 2006 | Journal of Epidemiology & Community Health | Specialy |
| 16 | White IR | 2005 | Statistical Methods in Medical Research | Statistics |
| 17 | Weinstein GS et al. | 1989 | Annals of Thorac Surgery | Specialty |
| 18 | Chene G et al. | 1998 | Controlled clinical trials | Methods |
| 19 | D'Agostino RB et al. | 2004 | Journal of Dental Research | Specialty |
| 20 | Walter SD et al. | 2006 | Journal of Clinical Epidemiology | Methods |
| 21 | Viscoli C et al. | 1995 | European Journal of Cancer | Specialty |
| 22 | Daya S | 2006 | Best Practice & Research in Clinical Obstetrics & Gynaecology | Specialty |
| 23 | Irvine EJ et al. | 2006 | Gastroenterology | Specialty |
| 24 | Unnebrink K et al. | 2001 | Statistics in Medicine | Statistics |
| 25 | Farrington CP | 1993 | International Journal of Epidemiology | Methods |
| 26 | Tillmann HC et al. | 2001 | International Journal of Clinical Pharmacology and Therapeutics | Specialty |
| 27 | Tierney JF et al. | 2005 | International Journal of Epidemiology | Methods |
| 28 | Fergusson D et al. | 2002 | BMJ | General Medicine |
| 29 | Fleiss JL | 1992 | Journal of Periodontal Research | Specialty |
| 30 | Freedman DA | 2006 | Evaluation Review | Methods |
| 31 | Furukawa TA et al. | 2005 | International Clinical Psychopharmacology | Specialty |
| 32 | Gibaldi M et al. | 1997 | Issues in Clinical Pharmacology | Specialty |
| 33 | Glasziou PP | 1992 | Journal of Clinical Epidemiology | Methods |
| 34 | Thoma A | 2005 | Clinics in Plastic Surgery | Specialty |
| 35 | Streiner D et al. | 2001 | Evidence Based Mental Health | Specialty |
| 36 | Goetghebeur E et al. | 2002 | Epidemiologic Reviews | Methods |
| 37 | Gravel J et al. | 2007 | Clinical Trials | Methods |
| 38 | Green SB | 2000 | Oncology Clinics of North America | Specialty |
| 39 | Sorensen HT et al. | 2006 | Hepatology | Specialty |
| 40 | Soares I et al. | 2002 | Cardiologia Baseada Na Evidencia | Specialty |
| 41 | Newell | 1992 | International Journal of Epidemiology | Methods |
| 42 | Sheiner LB et al. | 1995 | Clinical Pharmacology & Therapeutics | Specialty |
| 43 | Shao J et al. | 2003 | Statistics in Medicine | Statistics |
| 44 | Hollis S | 2002 | Statistics in Medicine | Statistics |
| 45 | Hollis S et al. | 1999 | BMJ | General Medicine |
| 46 | Schoenfeld PS | 2005 | American Journal of Gastroenterology | Specialty |
| 47 | Sato T | 2001 | Statistics Medicine | Statistics |
| 48 | Jones B et al. | 1996 | BMJ | General Medicine |
| 49 | Sabin CA et al. | 2000 | HIV Clinical Trials | Specialty |
| 50 | Ruiz-Canela M et al. | 2000 | BMJ | General Medicine |
| 51 | Kleinman KP et al. | 1998 | Biometrics | Statistics |
| 52 | Korhonen PA et al. | 1999 | Statistics Medicine | Statistics |
| 53 | Kruse RL et al. | 2002 | Journal of Family Practice. | Specialty |
| 54 | Porta N et al. | 2007 | Journal of Clinical Epidemiology | Methods |
| 55 | Peace KE et al. | 1993 | Journal of Biopharmaceutical Statistics | Statistics |
| 56 | Nich C et al. | 2002 | Drug and Alcohol Dependence | Specialty |
| 57 | Law MG et al. | 1996 | Statistics In Medicine | Statistics |
| 58 | Lewis JA et al. | 1993 | Br J Cancer | Specialty |
| 59 | Newcombe RG | 1988 | Statistics In Medicine | Statistics |
| 60 | Loeys T et al. | 2003 | Biometrics | Statistics |
| 61 | Nagelkerke N et al. | 2000 | Statistics In Medicine | Statistics |
| 62 | Montori VM et al. | 2001 | Canadian Medical Association Journal | General Medicine |
| 63 | Lui KJ | 2007 | Statistics In Medicine | Statistics |
| 64 | Mahaniah KJ et al. | 2004 | The Journal of Family Practice | Specialty |
| 65 | Mittlbock M et al. | 1998 | Lifetime Data Analysis | Statistics |
| 66 | Altman DG et al. | 2001 | Annals of Internal Medicine | General Medicine |

**Appendix S4, Table 1** Articles (n=17) that held more than one possible definition of intention to treat (ITT) in relation to missing outcome data (MOD)

| **Author, year** | **‘Full follow up required under ITT’** | **‘ITT and MOD are separate issues’** | **‘ITT involves specific strategy for MOD’** |
| --- | --- | --- | --- |
| ^66^Altman, 2001 | **X** | **X** | **X** |
| ^4^Bailey, 1994 | **X** |  | **X** |
| ^14^Bubbar, 2006 |  | **X** | **X** |
| ^19^D'Agostino, 2004 |  | **X** | **X** |
| ^37^Gravel, 2007 | **X** |  | **X** |
| ^38^Green, 2000 | **X** |  | **X** |
| ^44^Hollis, 2002 | **X** |  | **X** |
| ^45^Hollis, 1999 | **X** |  | **X** |
| ^53^Kruse, 2002 | **X** |  | **X** |
| ^58^Lewis, 1993 | **X** | **X** | **X** |
| ^56^Nich, 2002 | **X** |  | **X** |
| ^47^Sato, 2001 | **X** |  | **X** |
| ^40^Soares, 2002 | **X** |  | **X** |
| ^10^Wiens, 2007 | **X** |  | **X** |
| ^16^White, 2005 |  | **X** | **X** |
| ^15^Whittaker, 2006 | **X** |  | **X** |
| ^6^Wright, 2003 | **X** |  | **X** |

**Appendix S4, Table 2** Articles (n=28) that argued ‘Intention to treat involves a specific strategy for missing outcome data’

| **Author** | **Specific Strategy for Dealing with Missing Outcome Data** | | | | | | | | | |
| --- | --- | --- | --- | --- | --- | --- | --- | --- | --- | --- |
|  | **CCA** | **WC** | **BC** | **All Had Outcome** | **All No Outcome** | **Using Available Data** | **LOCF** | **MI** | **SA** | **Other †** |
| ^66^Altman | X |  |  |  |  |  |  |  |  |  |
| ^4^Bailey* |  |  |  |  |  |  |  |  |  |  |
| ^12^Borm |  |  |  |  |  | X |  |  |  |  |
| ^14^Bubbar |  | X | X |  |  |  |  |  | X |  |
| ^19^D'Agostino |  |  |  |  |  |  | X | X | X |  |
| ^31^Furukawa |  |  |  | X | X |  |  |  | X |  |
| ^32^Gibaldi* |  |  |  |  |  |  |  |  |  |  |
| ^37^Gravel |  | X | X | X | X | X | X | X | X | X |
| ^38^Green* |  |  |  |  |  |  |  |  |  |  |
| ^44^Hollis |  | X | X |  |  |  | X |  | X | X |
| ^45^Hollis |  | X | X | X | X | X | X |  | X |  |
| ^23^Irvine |  |  |  |  | X |  | X |  | X |  |
| ^51^Kleinman | X |  |  |  |  | X | X |  |  | X |
| ^53^Kruse | X | X | X |  |  | X |  |  | X |  |
| ^58^Lewis |  |  |  |  |  | X |  |  |  | X |
| ^56^Nich |  |  |  | X |  | X | X |  |  |  |
| ^54^Porta |  |  |  |  |  |  |  |  | X |  |
| ^49^Sabin |  |  |  | X |  | X | X |  | X |  |
| ^47^Sato |  |  |  |  |  |  |  |  | X | X |
| ^43^Shao |  |  |  |  |  |  | X |  |  | X |
| ^40^Soares |  | X |  | X |  | X | X |  |  | X |
| ^35^Streiner |  | X |  |  |  |  | X |  |  | X |
| ^24^Unnebrink |  | X | X | X | X | X | X |  | X | X |
| ^16^White* |  |  |  |  |  |  |  |  |  |  |
| ^15^Whittaker |  |  |  |  |  |  | X |  | X | X |
| ^10^Wiens |  |  |  |  |  | X | X |  |  |  |
| ^8^Winnock |  | X |  | X |  |  |  |  |  |  |
| ^6^Wright |  |  |  |  |  | X |  | X | X | X |

**Abbreviations:** CCA = complete case analysis, WC = worst case scenario, BC = best case scenario, LOCF = last outcome carried forward, MI = multiple imputation strategy, and SA = sensitivity analysis; "X" indicates that the author(s) explicitly indicated that the particular strategy for dealing with loss to follow-up was desirable or at least acceptable.

* Four articles explicitly endorsed the “ITT involves a specific strategy for dealing with missing outcome data” definition but did not mention a particular imputation

**†** Authors could suggest more than 1 strategy under the ‘Other’ strategies category

Appendix S4, Table 3 Summary of the ‘other strategies’ mentioned by authors for handling missing outcome data (MOD) under intention to treat (ITT)

| **Author, year** | **‘Other strategies’ for dealing with MOD under ITT** |
| --- | --- |
| ^37^Gravel, 2007 | i. Regression models |
| ^44^Hollis, 2002 | i. Pattern mixture approach: special case of stratified analysis which stratifies the data by pattern of missing data is performed. |
| ^51^Kleinman, 1998 | i. Bayesian Framework to fit linear model |
| ^58^Lewis, 1993 | i. Define a new outcome measure not dependant on partially missing values. |
| ^47^Sato, 2001 | i. Pattern mixture approach |
| ^43^Shao, 2003 | i. Last observation analysis (different from LOCF) |
| ^40^Soares, 2002 | i. Implicit assumptions of good or poor outcome |
| ^35^Streiner, 2001 | i. Growth curve |
| ^15^Whittaker, 2006 | i. Group mean |
| ^6^Wright, 2003 | i. Mean Imputation  ii. Likelihood based strategies  iii. Regression analysis  iv. Hot-deck imputation  v. Applying standard statistics tests to rank data |
| ^24^Unnebrink, 2001 | i. Imputation of the mean of own group  ii. Imputation of mean of other group  iii. Regression based on observed patients of placebo group  iv. Regression based on observed patients of own group  v. Regression based on observed patients of other group  vi. Minimax-regression  vii. Ranking strategy according to Gould  viii. Ranking strategy according to Senn |

**References of included articles**

1. Akobeng AK. Understanding randomised controlled trials. Archives of Disease in Childhood 2005;90(8):840-4

2 Yusuf S, Garg R, Zucker D. Analyses by the intention-to-treat principle in randomized trials and databases. Pacing & Clinical Electrophysiology 1991;14(12):2078-82.

3 Armitage P. Attitudes in Clinical Trials. Statistics in Medicine 1998;17:2675-2683.

4 Bailey A, Crook A, Machin D. Statistical methods for clinical trials. Blood Reviews 1994;8(2):105-12

5 Bentzen SM. Towards evidence based radiation oncology: improving the design, analysis, and reporting of clinical outcome studies in radiotherapy 1998;46:5-18.

6 Wright CC, Sim J. Intention-to-treat approach to data from randomized controlled trials: a sensitivity analysis. Journal of Clinical Epidemiology 2003;56(9):833-42.

7 Witte S., Victor N., Some Problems with the Investigation of Noninferiority in Meta-analysis, Methods Information in Statistics, (2004),43:470-4.

8 Winnock M. Rancinan C. De Ledinghen V. Couzigou P. Chene G. What hides behind an intention-to-treat analysis?[comment]. [Comment. Letter] Hepatology. 33(4):1014-5, 2001 Apr.

9 Blackwelder WC. Current issues in clinical equivalence trials. Journal of Dental Research 2004;83 Spec No C:C113-5

10 Wiens BL, Zhao W. The role of intention to treat in analysis of noninferiority studies. Clinical Trials 2007;4(3):286-91.

11 Blume J, Peipert JF. Randomization in controlled clinical trials: why the flip of a coin is so important. Journal of the American Association of Gynecologic Laparoscopists 2004;11(3):320-5

12 Borm GF, Houben RMGJ, Welsing PMJ, Zielhuis GA. An investigation of clinical studies suggests those with multiple objectives should have at least 90% power for each endpoint. Journal of Clinical Epidemiology 2006;59(1):1-6.

13 Branson  M, Whitehead J. “A score test for binary data with patient non-compliance.” Statistics in Medicine (2003);22(20):3115-32.

14 Bubbar VK, Kreder HJ. The intention-to-treat principle: a primer for the orthopaedic surgeon. Journal of Bone & Joint Surgery - American Volume 2006;88(9):2097-9

15 Whittaker K, Sutton C, Burton C. Pragmatic randomised controlled trials in parenting research: the issue of intention to treat. Journal of Epidemiology & Community Health 2006;60(10):858-64

16 White IR. Uses and limitations of randomization-based efficacy estimators. Statistical Methods in Medical Research 2005;14(4):327-47

17 Weinstein, Gerald S., Levin, Bruce, Effect of Crossover on the Statistical Power of randomized studies, Annals of Thorac Surgery, 1998 48, 190-3.

18 Chene G, Morlat P, Leport C, Hafner R, Dequae L, Charreau I, et al. Intention-to-treat vs. on-treatment analyses of clinical trial data: experience from a study of pyrimethamine in the primary prophylaxis of toxoplasmosis in HIV-infected patients. ANRS 005/ACTG 154 Trial Group. Controlled Clinical Trials 1998;19(3):233-48

19 D'Agostino RB, Sr., Massaro JM. New developments in medical clinical trials. Journal of Dental Research 2004;83 Spec No C:C18-24.

20 Walter SD, Guyatt G, Montori VM, Cook R, Prasad K. A new preference-based analysis for randomized trials can estimate treatment acceptability and effect in compliant patients.[erratum appears in J Clin Epidemiol. 2007 Nov;60(11):1203]. Journal of Clinical Epidemiology 2006;59(7):685-96

21 Viscoli C, Bruzzi P, Glauser M. An approach to the design and implementation of clinical trials of empirical antibiotic therapy in febrile and neutropenic cancer patients. European Journal of Cancer 1995;31A(12):2013-22.

22 Daya S. Methodological issues in infertility research. Best Practice & Research in Clinical Obstetrics & Gynaecology 2006;20(6):779-97.

23 Irvine EJ, Whitehead WE, Chey WD, Matsueda K, Shaw M, et al. Design of treatment trials for functional gastrointestinal disorders. Gastroenterology 2006;130(5):1538-51

24 Unnebrink K, Windeler J. Intention-to-treat: methods for dealing with missing values in clinical trials of progressively deteriorating diseases. Statistics in Medicine 2001;20(24):3931-46

25 Farrington CP. “Intention-to-treat analyses in clinical trials and cohort studies.”[comment]. International Journal of Epidemiology (1993);22(3):566

26 Tillmann H.C., Sharpe N., Sponer G. and Wehling M., Does Intention-to-treat analysis answer all questions in long-term mortality trials? Considerations on the basis of the ANZ trial, International Journal of Clinical Pharmacology and Therapeutics, 2001, 39, 205-212.

27 Tierney JF, Stewart LA. Investigating patient exclusion bias in meta-analysis.[see comment]. International Journal of Epidemiology 2005;34(1):79-87

28 Fergusson D, Aaron SD, Guyatt G, Hebert P. Post-randomisation exclusions: the intention to treat principle and excluding patients from analysis. BMJ 2002;325(7365):652-4.

29 Fleiss JL. General design issues in efficacy, equivalency and superiority trials.[see comment]. Journal of Periodontal Research 1992;27(4 Pt 2):306-13; discussion 323-7.

30 Freedman DA. Statistical models for causation: what inferential leverage do they provide? Evaluation Review 2006;30(6):691-713

31 Furukawa TA, Cipriani A, Barbui C, Brambilla P, Watanabe N. Imputing response rates from means and standard deviations in meta-analyses. International Clinical Psychopharmacology 2005;20(1):49-52.

32 Gibaldi Milo, Sullivan Sean, Intention-to-Treat Analysis in Randomized Trials: Who Gets Counted?, Issues in Clinical Pharmacology, 1997;37:667-672

33 Glasziou PP. Meta-analysis adjusting for compliance: the example of screening for breast cancer. Journal of Clinical Epidemiology 1992;45(11):1251-6

34 Thoma A. Challenges in creating a good randomized controlled trial in hand surgery. Clinics in Plastic Surgery 2005;32(4):563-73

35 Streiner D, Geddes J. Intention to treat analysis in clinical trials when there are missing data. Evidence-Based Mental Health 2001;4(3):70-1

36 Goetghebeur E, Loeys T. Beyond intention to treat. Epidemiologic Reviews 2002;24(1):85-90.

37 Gravel J, Opatrny L, Shapiro S. The intention-to-treat approach in randomized controlled trials: are authors saying what they do and doing what they say? Clinical Trials 2007;4(4):350-6.

38 Green SB. Hypothesis testing in clinical trials. Hematology - Oncology Clinics of North America 2000;14(4):785-95.

39 Sorensen HT, Lash TL, Rothman KJ. Beyond randomized controlled trials: a critical comparison of trials with nonrandomized studies. Hepatology 2006;44(5):1075-82.

40 Soares I. Carneiro AV. Intention-to-treat analysis in clinical trials: principles and practical importance. [Review] [14 refs] [Journal Article. Review] Revista Portuguesa de Cardiologia. 21(10):1191-8, 2002 Oct.

41 Newell DJ. Intention-to-treat analysis: implications for quantitative and qualitative research.[see comment]. International Journal of Epidemiology 1992;21(5):837-41

42 Sheiner LB, Rubin DB. Intention-to-treat analysis and the goals of clinical trials. Clinical Pharmacology & Therapeutics 1995;57(1):6-15

43 Shao J, Zhong B. Last observation carry-forward and last observation analysis.[see comment]. Statistics in Medicine 2003;22(15):2429-41

44 Hollis S. A graphical sensitivity analysis for clinical trials with non-ignorable missing binary outcome. Statistics in Medicine 2002;21(24):3823-34.

45 Hollis S. Campbell F. What is meant by intention to treat analysis? Survey of published randomised controlled trials.[see comment]. [Review] [39 refs] [Journal Article. Review] BMJ. 319(7211):670-4, 1999 Sep 11.

46 Schoenfeld PS. Evidence-based medicine in practice: applying intention-to-treat analysis and perprotocol analysis. [Case Reports. Journal Article] American Journal of Gastroenterology. 100(1):3-4, 2005 Jan

47 Sato T. A method for the analysis of repeated binary outcomes in randomized clinical trials with non-compliance. Statistics in Medicine 2001;20(17-18):2761-74.

48 Jones B, Jarvis P, Lewis JA, Ebbutt AF. Trials to assess equivalence: the importance of rigorous methods.[see comment][erratum appears in BMJ 1996 Aug 31;313(7056):550]. BMJ 1996;313(7048):36-9

49 Sabin CA, Lepri AC, Phillips AN. A practical guide to applying the intention-to-treat principle to clinical trials in HIV infection. HIV Clinical Trials 2000;1(2):31-8.

50 Ruiz-Canela M. Martinez-Gonzalez MA. de Irala-Estevez J. “Intention to treat analysis is related to methodological quality”.[comment]. [Comment. Letter] BMJ(2000). 320(7240):1007-8.

51 Kleinman KP, Ibrahim JG, Laird NM. A Bayesian framework for intent-to-treat analysis with missing data. Biometrics 1998;54(1):265-78

52 Korhonen PA, Laird NM, Palmgren J. Correcting for non-compliance in randomized trials: an application to the ATBC Study. Statistics in Medicine 1999;18(21):2879-97

53 Kruse RL. Alper BS. Reust C. Stevermer JJ. Shannon S. Williams RH. Intention-to-treat analysis: who is in? Who is out?[erratum appears in J Fam Pract. 2002 Dec;51(12):1079.]. [Review] [12 refs] [Comparative Study. Journal Article. Review] Journal of Family Practice. 51(11):969-71, 2002 Nov.

54 Porta N. Bonet C. Cobo E. Discordance between reported intention-to-treat and per protocol analyses. [Review] [37 refs] [Comparative Study. Journal Article. Research Support, Non-U.S. Gov't. Review] Journal of Clinical Epidemiology. 60(7):663-9, 2007 Jul.

55 Peace Karl E., Carter Hans Jr., Exposure Analysis of Dichotomous Response Measures in Long-Term Studies, Journal of Biopharmaceutical Statistics, (1993), 3(1), 129-140

56 Nich C. Carroll KM. Intention-to-treat meets missing data: implications of alternate strategies for analyzing clinical trials data. [Comparative Study. Journal Article. Research Support, U.S. Gov't, P.H.S.] Drug & Alcohol Dependence. 68(2):121-30, 2002 Oct 1.

57 Law Matthew G., Kaldor John M., Survival Analyses of Randomized Clinical Trials Adjusted For Patients Who Switch Treatments, Statistics In Medicine, (1996),15, 2069-2076.

58 Lewis J.A. Machin D. Intention to treat - who should use ITT?. British Journal of Cancer. 68:647-650, 1993.

59 Newcombe R.G., Explanatory and Pragmatic Estimates of the Treatment Effect When Deviations From Allocated Treatment Occur, Statistics In Medicine, (1988), 7. 1179-1186

60 Loeys T, Goetghebeur E. A causal proportional hazards estimator for the effect of treatment actually received in a randomized trial with all-or-nothing compliance. Biometrics 2003;59(1):100-5

61 Nagelkerke N, Fidler V, Bernsen R, Borgdorff M. Estimating treatment effects in randomized clinical trials in the presence of non-compliance.[erratum appears in Stat Med 2001 Mar 30;20(6):982]. Statistics in Medicine 2000;19(14):1849-64.

62 Montori VM. Guyatt GH. Intention-to-treat principle. [Review] [8 refs] [Journal Article. Research Support, Non-U.S. Gov't. Review] CMAJ Canadian Medical Association Journal. 165(10):1339-41, 2001 Nov 13.

63 Lui K-J. Interval estimation of the risk difference in non-compliance randomized trials with repeated binary measurements. Statistics in Medicine 2007;26(16):3140-56.

64 Mahaniah KJ. Rao G. Intention-to-treat analysis: protecting the integrity of randomization. [Journal Article] Journal of Family Practice. 53(8):644, 2004 Aug.

65 Mittlbock M, Whitehead J. “The interpretation of clinical trials of immediate versus delayed therapy. Lifetime Data Analysis “(1998);4(3):253-63.

66 Altman DG., DSc; Kenneth F. Schulz, PhD; David Moher, MSc; Matthias Egger, MD; Frank Davidoff, MD; Diana Elbourne, PhD; Peter C. Gøtzsche, MD; and Thomas Lang, MA, The Revised CONSORT Statement for Reporting Randomized Trials: Explanation and Elaboration, Annals of Internal Medicine, 2001, 134:663-694.
